# Global prioritised indicators for measuring WHO’s quality-of-care standards for small and/or sick newborns in health facilities: development, global consultation and expert consensus

**DOI:** 10.1136/bmjopen-2025-100338

**Published:** 2025-10-20

**Authors:** Louise T Day, Lara M E Vaz, Katherine E A Semrau, Sarah Moxon, Susan Niermeyer, Neena Khadka, Tamar Chitashvili, Gregory C Valentine, Mary Drake, Danielle E Y Ehret, Ashley Sheffel, Emma Sacks, Leah Greenspan, Theresa R Shaver, Lily Kak, Tedbabe Degefie Hailegebriel, Gagan Gupta, Kathleen Hill, Debra Jackson

**Affiliations:** 1 Department of Infectious Disease Epidemiology and International Health, London School of Hygiene & Tropical Medicine, London, UK; 2 Department of Health Behavior, University of North Carolina at Chapel Hill, Chapel Hill, North Carolina, USA; 3 Ariadne Labs, Brigham and Women’s Hospital and Harvard TH Chan School of Public Health, Boston, Massachusetts, USA; 4 Division of Global Health Equity and Department of Medicine, Brigham and Women’s Hospital, Boston, Massachusetts, USA; 5 Department of Pediatrics, Section of Neonatology, University of Colorado School of Medicine and Colorado School of Public Health, Aurora, Colorado, USA; 6 Global Health, Save the Children Federation, Washington, District of Columbia, USA; 7 John Snow International, Arlington, Virginia, USA; 8 University of Washington, Seattle, Washington, USA; 9 Jhpiego Corporation, Baltimore, Maryland, USA; 10 Vermont Oxford Network, Burlington, Vermont, USA; 11 University of Vermont Larner College of Medicine, Burlington, Vermont, USA; 12 Department of International Health, Johns Hopkins Bloomberg Sch Publ Hlth, Baltimore, Maryland, USA; 13 Public Health Institute contractor with the United States Agency for International Development, Washington, Virginia, USA; 14 Health Section, UNICEF, New York, New York, USA; 15 University of the Western Cape School of Public Health, Bellville, South Africa

**Keywords:** Quality in health care, Patient Reported Outcome Measures, NEONATOLOGY, Health Services

## Abstract

**Abstract:**

**Objectives:**

The aim of this study was to prioritise a set of indicators to measure World Health Organization (WHO) quality-of-care standards for small and/or sick newborns (SSNB) in health facilities. The hypothesis is that monitoring prioritised indicators can support accountability mechanisms, assess and drive progress, and compare performance in quality-of-care (QoC) at subnational levels.

**Design:**

Prospective, iterative, deductive, stepwise process to prioritise a list of QoC indicators organised around the WHO *Standards for improving the QoC for small and sick newborns in health facilities*. A technical working group (TWG) used an iterative four-step deductive process: (1) articulation of conceptual framework and method for indicator development; (2) comprehensive review of existing global SSNB-relevant indicators; (3) development of indicator selection criteria; and (4) selection of indicators through consultations with a wide range of stakeholders at country, regional and global levels.

**Setting:**

The indicators are prioritised for inpatient newborn care (typically called level 2 and 3 care) in high mortality/morbidity settings, where most preventable poor neonatal outcomes occur.

**Participants:**

The TWG included 24 technical experts and leaders in SSNB QoC programming selected by WHO. Global perspectives were synthesised from an online survey of 172 respondents who represented different countries and levels of the health system, and a wide range of perspectives, including ministries of health, research institutions, technical and implementing partners, health workers and independent experts.

**Results:**

The 30 prioritised SSNB QoC indicators include 27 with metadata and 3 requiring further development; together, they cover all eight standard domains of the WHO quality framework. Among the established indicators, 10 were adopted from existing indicators and 17 adapted. The list contains a balance of indicators measuring inputs (n=6), processes (n=12) and outcome/impact (n=9).

**Conclusions:**

The prioritised SSNB QoC indicators can be used at health facility, subnational and national levels, depending on the maturity of a country’s health information system. Their use in implementation, research and evaluation across diverse contexts has the potential to help drive action to improve quality of SSNB care. WHO and others could use this list for further prioritisation of a core set.

**STRENGTHS AND LIMITATIONS OF THIS STUDY**

- Used recommended stepwise process for global health quality indicator development, adopting and adapting existing indicators to align and strengthen routine data measurement efforts.
- Consulted a wide range of country, regional and global stakeholders, including 172 respondents to our online consultation in three languages, although parent voice was limited.
- The list of prioritised indicators balances (1) representation of each WHO small and/or sick newborn (SSNB) Quality Standard;^9^ (2) linkage to the survive and thrive elements of Global Strategy^4^ and (3) coverage of the entire 28-day neonatal period (days 0–28).
- Consensus regarding indicator prioritisation was complicated due to the large number of already prioritised SSNB indicators, which limited our ability to prioritise further and constrain the list.
- The prioritised indicators need implementation experience to learn how they can contribute to improvement in quality-of-care and guide further iteration.

## Introduction

 To accelerate progress toward global targets, focus is needed to transform care for the most vulnerable.^1^ Small and/or sick newborns (SSNBs) represent one in every four births worldwide and account for over half of the estimated 2.3 million neonatal deaths annually.^2^ Currently, more than 60 countries will not meet either the 2030 Sustainable Development Goal (SDG) target for reducing neonatal mortality or the global target for reducing stillbirths in the Every Woman Every Newborn Everywhere (EWENE) (previously the Every Newborn Action Plan) and the Global Strategy for Women, Children’s and Adolescents’ Health.^3^,^6^

Small newborns, defined as low birthweight (LBW) if weighing <2500 g at birth, include both preterm and growth-restricted term neonates.^1^ Sick newborns are those who experience medical or surgical conditions during their first month of life. To survive and thrive, most SSNBs require specialised care and often multiple interventions during their hospital stay. A model for scaling up SSNB care was published in 2023.^7^

**Figure 1:**
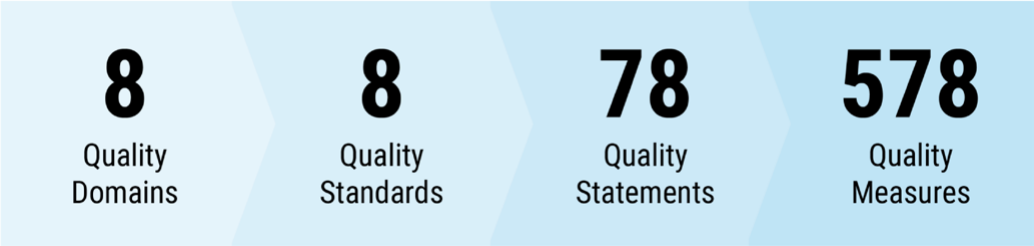
Overview of number of quality domains, standards, statements and measures in the WHO standards for improving the quality-of-care for small and sick newborns in health facilities.

Quality-of-care (QoC) encompasses not only the content of care but also the positive experience of care for newborns and their caregivers.^8^ In 2020, the WHO published *Standards for improving the QoC for small and sick newborns in health facilities* (hereafter *WHO SSNB Quality Standards*); these are part of a WHO publication series covering the continuum of care across the life course.^9^ The standards encompass eight interacting domains of quality recommended to be assessed, improved and monitored across health system levels. These standards link to 78 quality statements that define the requirements for QoC for SSNBs, which in turn link to 578 quality measures that define the criteria for assessing, monitoring and evaluating QoC for SSNBs (figure 1).^9^

We evaluated these SSNB measures and previously recommended newborn health global indicators to develop a list of prioritised SSNB QoC indicators. This aligns with the WHO ambition to provide guidance on global indicators for measuring and monitoring QoC across life stages.^10^ These SSNB QoC indicators are meant to be collected primarily at health facilities providing care to SSNBs but can be used by stakeholders at different levels of the health system, especially subnational levels but also relevant for national and global levels.^9^ Monitoring these indicators can support accountability mechanisms, assess and drive progress and compare performance in QoC.

Here, we aim to describe the methods and approach used to develop, prioritise and define the indicators and to delineate the recommendations emerging from this effort. This work follows previous WHO publications on sets of prioritised indicators for measuring QoC for maternal and newborn health and for children and young adolescents in health facilities.^10^,^13^

## Methods

**Figure 2:**
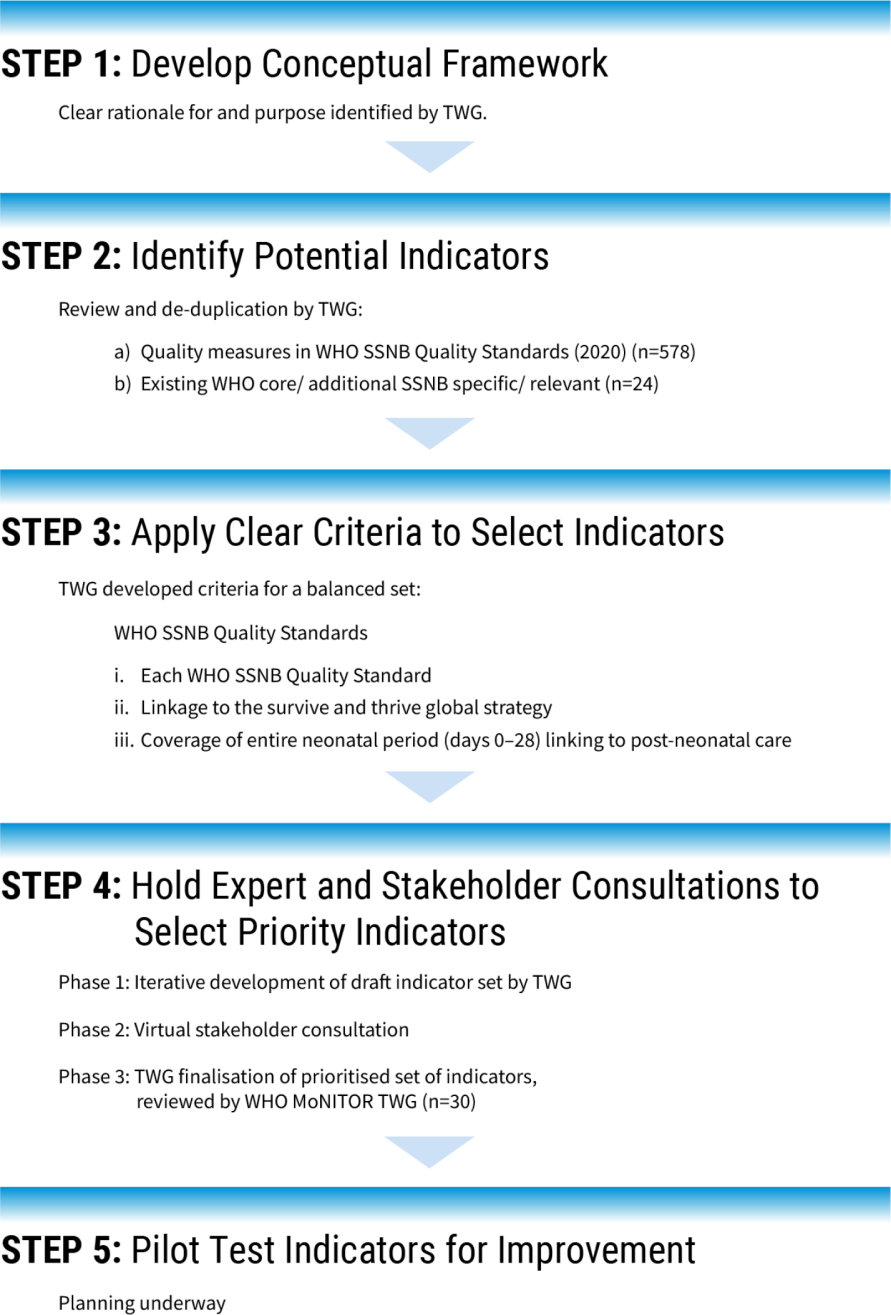
Overview of process to identify prioritised set of indicators for quality-of-care for small and sick newborns. MoNITOR, Mother and Newborn Information for Tracking Outcomes and Results; SSNB, small and/or sick newborns; TWG, technical working group.

The process to prioritise SSNB QoC indicators involved an approach agreed on by a WHO-led time-limited technical working group (TWG). The TWG implemented an iterative deductive, stepwise process of indicator development (figure 2) as described in a recent scoping review of methodological practices for developing QoC indicators.^14^ The SSNB QoC TWG members represented interprofessional expertise in neonatal health, quality improvement, measurement and experience supporting newborn programmes in high and low mortality/morbidity settings (online supplemental material 1).

### Step 1: developing a conceptual framework

The primary organising framework used to prioritise indicators for measuring SSNB QoC was the *WHO SSNB Quality Standards*.^9^ We prioritised indicators with global relevance, particularly targeting settings with high neonatal mortality and morbidity. We intended the primary users to be managers of national and subnational newborn programmes and health facility clinicians with a goal that the indicators could be feasibly incorporated and used by national programmes within the next decade.

**Figure 3:**
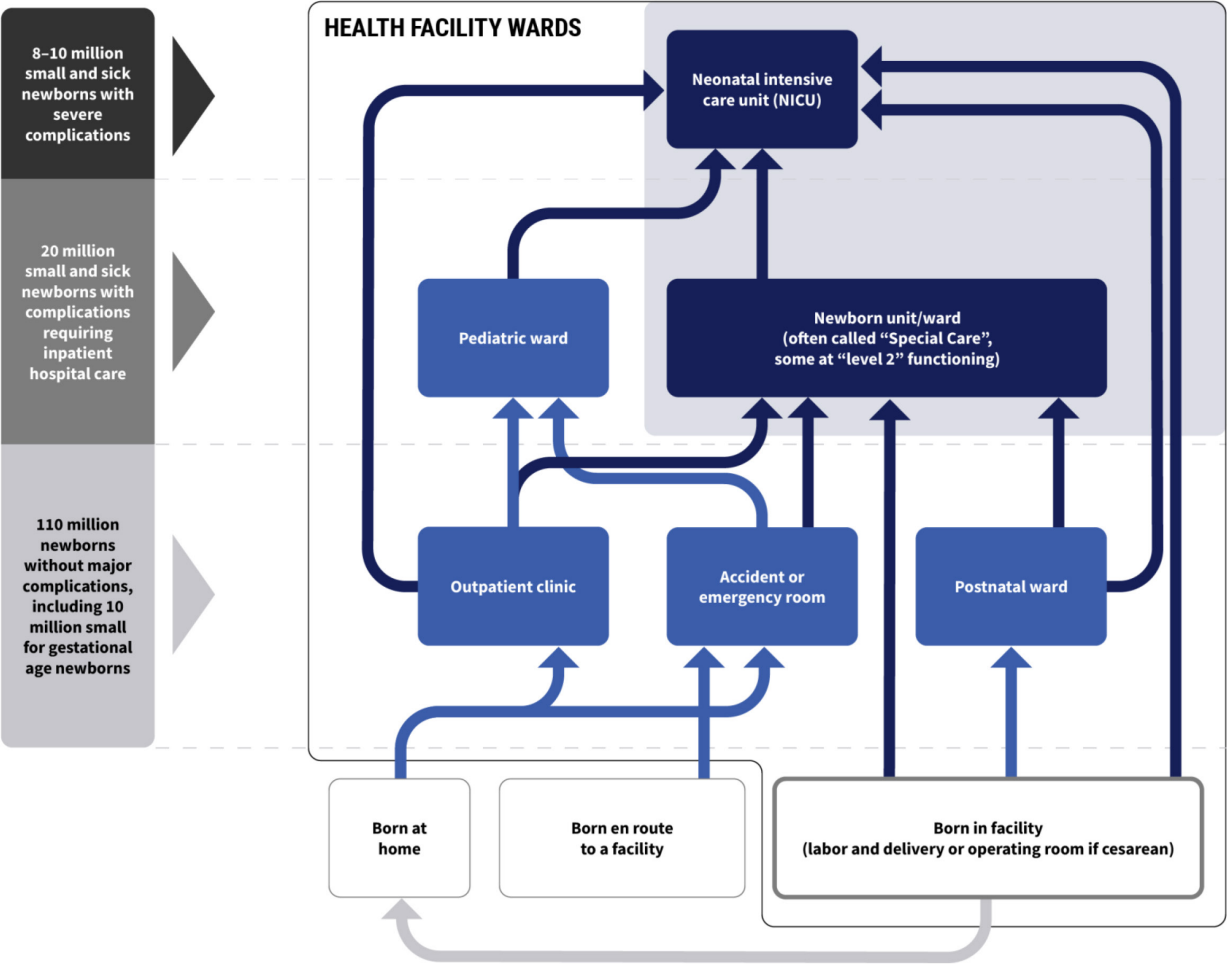
Global estimated number of newborns categorised by care needs each year, with place of birth and pathway to recommended health system location of care.

Because SSNBs commonly receive care spanning multiple wards in health facilities, we delineated denominators that allow accurate interpretation and use of measurements. The TWG focused on two groups to represent the full SSNB population, cared for in multiple ward locations in health facilities, while facilitating efficient indicator measurement and use (figure 3):

**Group A)** SSNBs in all health facility wards (labour and delivery ward, postnatal ward, Kangaroo Mother Care (KMC) ward, neonatal wards/units, paediatric wards). **Group B)** SSNBs admitted to inpatient neonatal units/wards level 2 or above, aligning with the EWENE coverage targets.^1 6^

### Step 2: identification of potential indicators

The TWG identified potential indicators by listing, cross-linking and deduplicating potential SSNB indicators. First, the TWG reviewed the 578 quality measures in the *WHO SSNB Quality Standards*, which were previously prioritised by virtual Delphi method.^9^ Second, the TWG reviewed indicators specific to or relevant for SSNB from other WHO-approved measurement frameworks. The TWG aimed to identify indicators that potentially could be *adopted* or *adapted* as relevant for SSNB QoC measurement based on previous prioritisation initiatives (online supplemental material 2). Indicators pertaining to care and outcomes for well and/or term newborns were excluded.

### Step 3: defining the criteria for selection of indicators

The TWG agreed on criteria for a balanced and coherent group of indicators for SSNB QoC.^15^ Criteria included: (1) representation of each WHO SSNB Quality Standard^9^; (2) linkage to the survive and thrive elements of Global Strategy^4^ and (3) coverage of the entire 28-day neonatal period (days 0–28) with connection to postneonatal care (online supplemental material 3).^1^ Indicators were ranked and evaluated based on ‘good indicators’ criteria (action-focused, important, simple and valued, operational, feasible) and the degree of content validity to a variety of global contexts (online supplemental materials 4-6).^16 17^ We prioritised process measures above structures and outcomes, as process measures are more amenable to timely change and therefore useful to assess if system changes are leading to intended improvements.^18^ Feasibility of indicator measurement using available data sources, methodologies and/or tools was an independent criterion, given its importance for implementation. Existing data sources, methodologies and tools deemed feasible to operationalise these indicators included routine health information system (RHIS) data, civil registration and vital statistics (CRVS), health facility assessment or district supervision visits and parent and caregiver health worker interviews. The step included consultation with the WHO Mother and Newborn Information for Tracking Outcomes and Results (MoNITOR) Technical Advisory Group.^19^

### Step 4: expert and stakeholder consultations

Using a three-phase iterative consultation process, we used the scoring criteria to gather the perspectives of the TWG and country, regional and global stakeholders on the potential indicators.

#### Phase 1: TWG-led development of draft indicators

Two researchers with complementary interprofessional skills (LTD and SM) independently scored the *WHO SSNB Quality Standards* measures. The highest scoring measures were then compiled into a list of potential QoC indicators that was independently scored by the TWG members. Mean scores were then grouped into tertiles: highest (7.5–10), middle (5–7.4) and lowest (0–4.9). The list was refined based on highest scores and feasible data sources. Another round of scoring by the TWG was completed with a virtual meeting to reach consensus on potential indicators. Indicators already listed in other global monitoring initiatives (identified in Step 2) were added to the draft list for wider consultation (online supplemental material 7).

#### Phase 2: stakeholder consultation survey on draft indicators

In May 2022, the TWG conducted a consultation in English, French and Spanish using an online survey and document review. We sought input from stakeholders, including individuals representing parents, ministries of health, health workers/professional organisations, implementing partners, TWGs, researchers, independent experts and WHO and UNICEF regional and country offices. Email survey invitations were sent purposively to WHO and UNICEF regional and country offices for circulation to ministries of health, health workers/professional organisations, implementing partners and linked WHO technical advisory groups. The TWG also circulated the survey to researchers and independent experts. All recipients were requested to forward the link to any partners they considered would be interested in providing feedback (online supplemental material 8).

Survey respondents chose one country context they knew well and answered questions regarding potential SSNB QoC indicators in that specific setting. For each proposed indicator, respondents used a 4-point Likert scale to score the indicators on usefulness and measurement feasibility and their recommendation for inclusion as prioritised versus additional indicator; respondents could provide optional open-ended comments for each question. Two additional questions explored the respondents’ opinions on whether the group or set of indicators was balanced to capture information (1) across the 28-day neonatal period; and (2) linked to the Survive and Thrive aspects of the Global Strategy.^4^ Full details of the stakeholder consultation are reported in online supplemental material 8.

**Figure 4:**
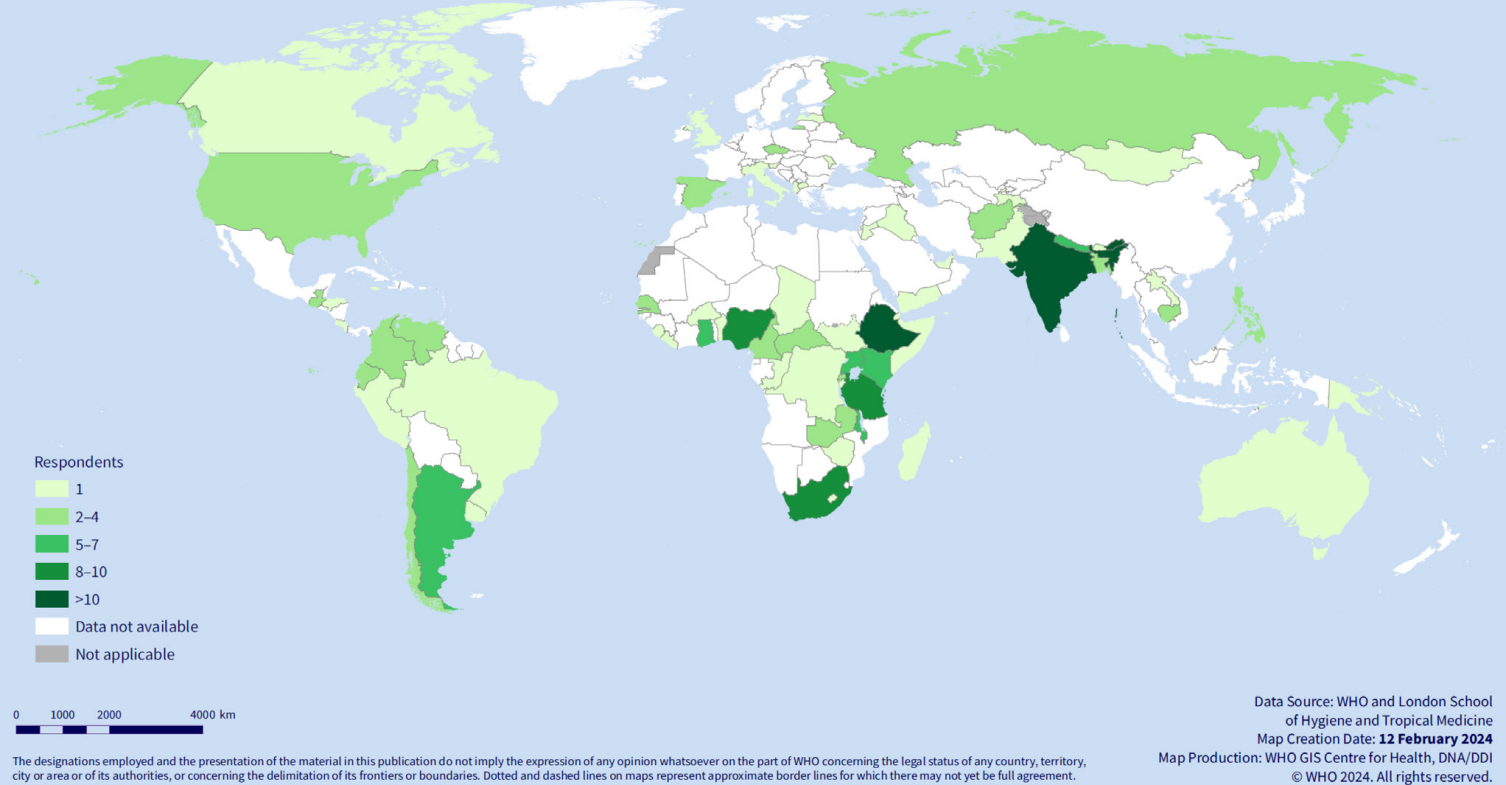
Distribution of stakeholder consultation responses on draft indicator set across WHO member states. DDI, Division of Data, Analytics, and Delivery for Impact; DNA, Department of Data and Analytics; GIS, geographic information system.

Responses to the online survey (n=172) were submitted either on behalf of an organisation or individually. The respondents’ background characteristics are shown in online supplemental material 9. Respondents contributed information based on their country-level experience (n=71 individual countries, with the following respondents’ country-level income category: 74 (43%) lower-middle income, 45 (26%) low-income, 34 (20%) upper-middle income and 19 (11%) high-income countries (figure 4, online supplemental material 10).

#### Phase 3: TWG-led revision of indicator list and development of metadata

The TWG reviewed the stakeholders’ inputs to identify indicators scoring highly (online supplemental materials 11-13). Several virtual TWG meetings were convened to revise and balance the final prioritised indicators using the agreed criteria through iterative discussion (Step 3). The indicator metadata (definition, classification, numerator, denominator, data source and disaggregation strata etc) were developed by the TWG; where possible, indicators were reworded to the positive or desired outcome of interest (eg, survival rather than death, normothermia rather than hypothermia). The TWG rationale for indicator development and selection is detailed in online supplemental material 13. The revised indicator list was reviewed by MoNITOR.^19^

### Data management and analysis

Phase 1 indicator mapping and scoring was conducted using Microsoft Excel workbooks; data were imported into R and mean scores calculated.^20 21^ Phase 2 online consultation was managed using Jisc Online surveys software and analysed in R.^21 22^ For the SSNB QoC indicator output, we used the reporting standards for guideline-based performance measure development and re-evaluation (online supplemental material 14).^23^

### Role of the funding source

The study funders did not contribute to study design or conduct. All authors have full access to study data with final responsibility for publication submission decision.

### Patient and public involvement

None.

## Results

The final 30 prioritised indicators to measure QoC are split across the two agreed denominators: **Groups A)** 10 indicators adopted from previous indicator lists apply to SSNB cared for in all health facility wards which were adopted from previous indicator lists; **Group B)** 17 defined indicators and 3 requiring further definition apply to the subset of SSNB admitted to neonatal wards/units only (table 1, online supplemental material 13).

**Table 1:** Prioritised indicators to measure quality-of-care (QoC) for small and/or sick newborns (SSNB)

Context/quality domain Standard # /framework outcome	Indicator name	Indicator definition	Indicator type (WHO)—Paed QoC, otherwise MoNITOR (Donabedian)	Prioritised indicator list alignment and definition*
**Group A) All SSNB cared for in all health facility wards (labour and delivery ward, postnatal ward, KMC ward, neonatal wards/units) (n=10 indicators**)				
**Context**	Inpatient admissions among newborns	# or % of inpatient admissions among newborns.	Output/process (*Donabedian=process*)	MoNITOR,^41^ MNCAH,^31^ COVID-19 routine data^42^
Quality domain 1	Neonatal resuscitation	% of newborns who received positive-pressure ventilation at birth	Process/output (*Donabedian=process*)	ENAP coverage,^43 44^ MNCAH^31^
Quality domain 1	Kangaroo Mother Care (KMC)† initiated	% of admitted neonates with a birthweight <2500 g who were initiated on KMC† anywhere in the health facility	Process/output (*Donabedian=process*)	ENAP coverage,^43 44^ MNCAH^31^
Quality domain 1	Antibiotic treatment for neonatal infection	% of neonates (0–28 days) identified as clinically suspected sepsis (serious bacterial infection)‡ in inpatient settings, who received at least two days of appropriate injectable antibiotics.	Process/output (*Donabedian=process*)	ENAP coverage,^43 44^ MNCAH^31^
Quality domain 1	Antenatal corticosteroids	% of women who delivered between 24 and 34 weeks gestational age who received at least one dose of antenatal corticosteroids.	Process/output (*Donabedian=process*)	ENAP coverage,^43 44^ MNCAH^31^
**Facility/individual level outcome**	In-facility§ late gestation stillbirth‡‡‡	% of in-facility§ stillbirths among late gestation‡‡‡ total births	Impact (*Donabedian=outcome*)	UNICEF,^45^ ICD 11^46^
	Low birthweight (<2500 g)	Proportion of live births in facilities with birthweight <2500 g.	Outcome (*Donabedian=outcome*)	MNCAH^31^ MoNITOR,^41^
	Preterm birth	% of births in facilities that are preterm (<37 completed weeks gestation).	Outcome (*Donabedian=outcome*)	MoNITOR,^41^ MNCAH^31^
	Small for gestational age	% of neonates with birthweight below the 10th percentile of the expected weight for gestational age of the reference population.	Outcome (*Donabedian=outcome*)	MoNITOR^41^
	Institutional neonatal mortality	% of inpatient neonatal deaths 0–27 days in health facilities.	Impact (*Donabedian=outcome*)	MNCAH,^31^ Health Facility Indicator,^32^ QoC MNH,^33^ QoC Child^10^
	Neonatal cause of death in health facilities	% of neonatal deaths (days 0–27 of life) of a specified cause.	Impact (*Donabedian=outcome*)	MoNITOR,^41^ Health Facility Indicator,^32^ QoC MNH^33^
**Group B) Subset of SSNB admitted to neonatal wards/ units only: defined (n=17 indicators), needing definitional work (n=3 indicators**)				
**Context**	Level 2 inpatient unit for small and/or sick newborns	% of districts/subnational areas with one or more level 2 neonatal units.	Input (*Donabedian=structure*)	ENAP coverage target^34^
Quality domain 1	Assessment of gestational age	% of neonates less than 96 hours of age who are admitted to a neonatal unit with unknown gestational age and are assessed with an appropriate gestational age scoring tool.	Process/output (patient level) (*Donabedian=process*)	New from WHO SSNB Quality Standards^9^
Quality domain 1	Screening and treatment of retinopathy of prematurity (ROP)	% of neonatal units providing regular** retinopathy of prematurity (ROP) screening†† and treatment.‡‡	Input (*Donabedian=structure*)	New from WHO SSNB Quality Standards,^9^ Vermont Oxford Network^47 48^ general discussion with ROP experts LSHTM
Quality domain 2	Completion of standardised individual small and/or sick neonate case notes	% of neonatal case notes with complete key clinical information at admission§§ and discharge.¶¶	Process/output/(facility level) (*Donabedian=process*)	Adapted from QoC Child^10^
Quality domain 3	Normothermia on admission	% of neonates with an admission temperature 36.5–37.5°C on admission to the neonatal unit.	Outcome (*Donabedian=outcome*)	New from WHO SSNB Quality Standards^9^
Quality domain 4	Use of SSNB quality-of-care indicators—displayed on neonate unit	% of neonatal units that publicly display trends for ≥3 SSNB QoC indicators accessible to health facility staff and families.	Process/output (facility level) (*Donabedian=process*)	Adapted from QoC Child^10^ to align with WHO SSNB Quality Standards^9^
Quality domain 5	Death notification/registration in civil registration and vital statistics system	% of institutional neonatal deaths notified/registered*** to the appropriate civil authority.	Process/output (facility level) (*Donabedian=process*)	New from WHO SSNB Quality Standards^9^
Quality domain 6	Developmentally supportive care	% of neonates admitted to the neonatal unit who experience skin-to-skin care (called KMC only if low birthweight) for two or more hours daily during admission.	Process/output (patient level) (*Donabedian=process*)	New from WHO SSNB Quality Standards^9^ and Nurturing Care Framework^49^
Quality domain 6	Follow-up for growth and development	% of neonates discharged from the neonatal unit who received timely risk-appropriate††† follow-up as per discharge plan.	Process/output (patient level) (*Donabedian=process*)	New from WHO SSNB Quality Standards^9^
Quality domain 7	Nurse/baby ratio on neonate unit	% of neonatal units regularly reporting nurse/baby ratios for every shift by type of ward.	Input (*Donabedian=structure*)	New from WHO SSNB Quality Standards^9^
Quality domain 7	Hand hygiene at facility unit entrance	% of neonatal units which have functioning (water and soap) hand hygiene station at the entrance.	Input (*Donabedian=structure*)	Adapted MoNITOR,^41^ QoC MNH^33^
Quality domain 8	Stockout of safe oxygen delivery systems	% of neonatal units with no stockout of safe oxygen delivery systems¶¶¶ in a specified period.	Input (*Donabedian=structure*)	Adapted Health Facility Indicator^32^
Quality domain 8	Stockouts of three essential neonate tracer medicines§§§	% of neonatal units reporting no stockout of three essential tracer medicines in correct formulations§§§ in a specified period.	Input (*Donabedian=structure*)	Adapted QoC Child^10^ and Health Facility Indicator^32^
Quality domain 8	Stockouts of three essential neonatal tracer devices, equipment and supplies¶	% of neonatal units reporting no stockout of three functioning essential neonatal tracer devices, equipment and supplies¶ in a specified period.	Input (*Donabedian=structure*)	Adapted Health Facility Indicator^32^
**Facility/individual level outcome**	Exclusive breast milk feeding at time of discharge	% of neonates exclusively breast milk fed (sucking, cup/tube fed) at discharge from the neonate unit.	Outcome (*Donabedian=outcome*)	Adapted from 100 prioritised health indicators,^50^ WHO SSNB Quality Standards,^9^ Child Health and well-being^51^
	Neonatal survival rate after inpatient care	% of neonatal survivors by birthweight category at facility discharge after admission to the neonatal unit.	Impact (*Donabedian=outcome*)	New from WHO SSNB Quality Standards^9^
	Infant and family-centred neonatal care	*Indicator to be defined—proposed topic content, see online supplemental material 16.*	Outcome (*Donabedian=outcome*)	New from WHO SSNB Quality Standards^9^
	Person-centred provider care	*Indicator to be defined—proposed topic content, see* *online supplemental material 17**.*	Outcome (*Donabedian=outcome*)	New from WHO SSNB Quality Standards^9^
	Neonatal thrive rate by birthweight group category	*Indicator to be defined.*	Impact (*Donabedian=outcome*)	New from WHO SSNB Quality Standards^9^

Standard=WHO SSNB Quality Standards.^5^

#=number.

* Indicators in table 1A are adopted from previously prioritised global indicator lists, referenced in the table, further information in online supplemental material 13.

† Initiated on Kangaroo mother care (KMC) means placed in the ‘kangaroo position’, defined as baby placed in an upright position, in direct skin-to-skin contact on the mother’s chest, secured in place using a wrap and/or binder.^52^

‡ Clinically suspected sepsis (serious bacterial infection). Danger signs include: not feeding well, convulsions, drowsy or unconscious, movement only when stimulated or no movement at all, fast breathing (60 breaths per min), grunting, severe chest in-drawing, raised temperature: >38°C, hypothermia: <35.5°C. Localising signs of infection are: signs of pneumonia, many or severe skin pustules, umbilical redness extending to the periumbilical skin, umbilicus draining pus, bulging fontanelle, painful joints, joint swelling, reduced movement and irritability if these parts are handled.^53^

§ Known to be alive on admission to the facility.^45^

¶ Devices: 1.digital weight scales 2.phototherapy 3.point-of-care glucose testing. For level 2 plus units and level 3 units, continuous positive airways pressure (CPAP) as a fourth tracer device can be added for tracking.

** Weekly visits or contact as needed for smaller units.

†† Indirect ophthalmological examination or with imaging and remote grading by trained ophthalmologists/technicians/neonatal nurses, as appropriate.

‡‡ Laser by indirect delivery or intravitreal injection by an ophthalmologist.

§§ Temperature, respiratory rate, weight, feeding status.

¶¶ Classification/diagnosis, treatment, counselling, care outcomes (weight, feeding status and place/time of follow-up, link to follow-up plans).

*** Context specific depends on legal frameworks to register deaths.

††† Context specific risk management by neonatal condition.

‡‡‡ 196 or more days gestation (≥28+0 weeks).^45^

§§§ Medicines:1. first-line injectable antibiotics,2. phenobarbital 3. caffeine (or other methylxanthines)

¶¶¶ adjustable FiO2 using pulse oximetry.

ENAP, Every Newborn Action Plan; FiO2, fractional inspired oxygen; ICD 11, International Classification of Diseases 11th Revision; LSHTM, London School of Hygiene & Tropical Medicine; MNCAH, maternal, newborn, child and adolescent health; MNH, maternal and newborn health; MoNITOR, Mother and Newborn Information for Tracking Outcomes and Results WHO Technical Advisory Group; QoC, quality-of-care.

**Figure 5:**
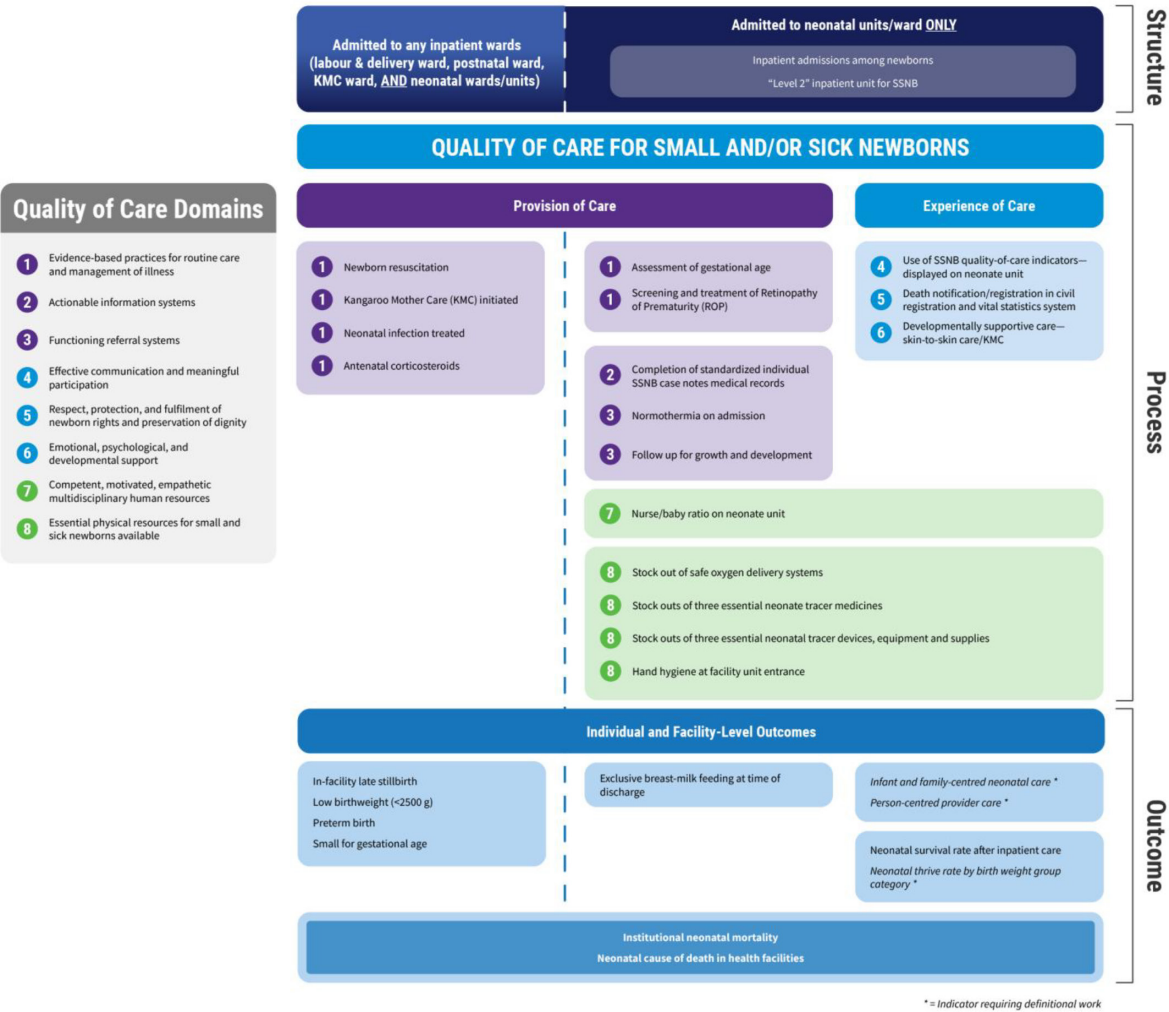
Prioritised list of global indicators for measuring WHO’s Small and Sick Newborn quality-of-care standards in health facilities (n=30), mapped to ward of care, quality-of-care framework and data source. SSNB, small and/or sick newborns.

Figure 5 shows the distribution of the indicator list by the selection criteria, including by place of care, and the eight quality domains. Two prioritised indicators relate specifically to the SSNB context: the subnational availability of neonatal units/wards by geography, and the number of SSNB admissions for inpatient care, disaggregated by location of care. Six of the indicators tied to care in neonatal units/wards (**Group B**) (35%) are input indicators, eight (47%) relate to processes at either the individual patient or facility level and three (18%) are outcome/impact indicators. Three complementary data sources were proposed to measure these 17 indicators: RHIS patient data (n=10), RHIS facility data (n=6), supervisory visit/Health Facility Assessment (n=2).

Three indicators require further definition. These describe important aspects of SSNB care: infant-centred and family-centred developmental care (IFCDC), person-centred provider care and the novel concept of a neonatal thrive rate; they require additional data sources, including parent and health provider interviews (online supplemental materials 15,16, respectively). Proposed additional QoC indicators emerging from our discussions for countries with higher levels of maturity of RHIS are described in online supplemental material 17.

The prioritised indicators are balanced across the eight WHO quality standards, type of indicator (input, process/output, outcome, impact) and the survive and thrive elements of the Global Strategy.^17^ The QoC indicators prioritised for SSNB admitted to newborn wards and units (Group B) support EWENE efforts to increase the availability of and access to recommended inpatient care for SSNB at district level (EWENE coverage target 4).^6^

This work completes the first four of five recommended steps for developing QoC indicators for global health.^14^ The prioritised indicators are ready for use and experiential learning within countries’ contextualised models of SSNB care.^7^

## Discussion

### Alignment with the scale-up model of SSNB care

The proposed indicators align with the 10 components for scale-up of the model of SSNB care developed through a WHO/UNICEF expert and country consultation.^7^ This SSNB model focuses on health system components to achieve scale-up for level 2 SSNB facilities. Where possible, we selected indicators measured by RHIS to strengthen data use and data quality through feedback cycles, thereby supporting prioritised component 6 of the scale-up model: robust data systems and use of data.^7^ Since data documented by frontline health workers in registers and clinical case notes are used for RHIS aggregate patient data, we prioritised indicators using patient-level clinical data for efficient dual use of data for real-time decision-making for care and programme management across health system levels.^24^ This aligns with previous research in high mortality settings.^25^

High-quality intrapartum care (model component 7) has benefits for survival and well-being of both mother and infants, underlying the adoption of indicators, including antenatal corticosteroids and neonatal resuscitation after birth.^7 26^ The inclusion of intrapartum stillbirth in our indicator list echoes the dual ambition of EWENE to end both preventable stillbirth and neonatal mortality.^6^

An IFCDC approach is central to high-quality SSNB care.^1^ Family involvement and developmentally supportive care, including responsive caregiving (model components 8 and 9), are represented in the list by an ambitious indicator to capture the proportion of admitted SSNB who receive skin-to-skin care for 2 hours or more each day.^1 7^ Two other IFCDC measures are included among the suggested items for a family-centred scale. Such indicators require further development, perhaps using a similar methodology to the person-centred maternity care score^27^ and the emerging topic of newborn experience of care.^28^

The prioritised SSNB QoC indicators capture tracer measures throughout the neonatal period to support the provision of consistent, actionable, high-quality care. For example, assessment of the proportion of newborns normothermic on admission is actionable to improve thermal care practices to avoid negative physiological cascades, such as poor feeding, hypoglycaemia and apnoea which mediate hypothermia as a predictor of mortality.^29 30^ Most indicators capture care during inpatient stays; the inclusion of one postdischarge care (model component 10) indicator in the prioritised list (follow-up of growth and development) reflects the current challenges in longitudinal data collection.^7^

### Measurement feasibility

Indicators will only benefit quality improvement processes if users have access to credible data. The feasibility of high-quality data collection, especially in high-mortality settings, was therefore central to our indicator selection process. We aimed to strike a balance between prioritising indicators essential to improve SSNB QoC and those that are feasible to collect. We began by prioritising indicators that could be adopted or adapted from existing lists to strengthen measurement efforts and minimise burden. The indicators adopted from existing guidance were, as expected, the highest ranked for measurement feasibility by the global online survey respondents. Among these, three indicators (KMC initiation, neonatal resuscitation and neonatal sepsis) recently had refined definitions by WHO.^31^

Overall, online survey respondents, representing wide implementation and measurement experience, indicated QoC measurement in most settings will require additional health system investment to be feasible within the SDG era. This aligned with our decision to focus on one clear group of SSNBs admitted to newborn wards as the denominator for most proposed indicators. These are the smallest, sickest and most vulnerable to adverse outcomes resulting from poor QoC. Moreover, this newborn inpatient group is the focus of country and global implementation efforts to end preventable mortality aligning with EWENE coverage target 4.^1 7^ The data elements needed for this proposed list of SSNB QoC indicators are mostly captured daily by individual clinical case notes, thus a priority to be captured by health workers. Supporting efficient use of clinical information for indicator measurement is a priority to improve indicator measurement feasibility.^6^ WHO is leading multi-phase work to standardise medical records.

Many of the new indicators resonate with work in other priority areas (eg, notification/registration in CRVS after neonatal death, cause of death for maternal and perinatal death surveillance and response), which reinforces and complements existing efforts to generate and use high quality data for decision-making.^31^,^36^

Countries are encouraged to use this list of QoC indicators as they scale up SSNB care, beginning with indicators that are more easily measurable and with gradual expansion to the complete list when appropriate.^7^ As these SSNB quality indicators are implemented, shared learning on the experience and use of these indicators from all stakeholders at facility, subnational and national levels in diverse settings has the potential to rapidly improve uptake and utility of this prioritised indicator list. For settings with more mature RHIS, we provide additional indicators that can contribute to further strengthening of SSNB QoC (online supplemental material 17).

### The path ahead

**Figure 6:**
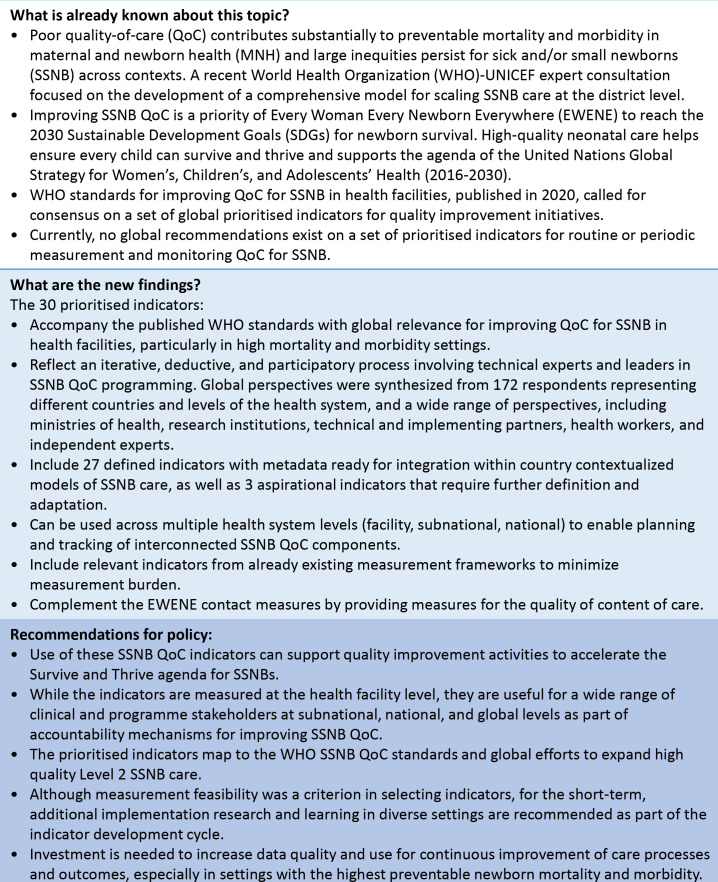
Key messages for global prioritised indicators for measuring WHO’s quality-of-care standards for small and/or sick newborns in health facilities

Recommendations for policy based on the findings of our work, linked to existing knowledge on this topic, are shown in figure 6. Based on Donabedian QoC theory, we included indicators of structure, process and outcome.^18^ Quality indicator measurement should not be limited by current knowledge regarding individual indicators or RHIS performance; thus, the set is ambitious to measure the high-quality care SSNBs are entitled to, balanced with the practicality of needing data for action now. Most indicators (n=27) in the list have metadata ready for immediate use. The development of an implementation guide for the primary audience (subnational and facility level programme managers) will be important; it should include suggestions to overcome barriers to enable high quality data and using the indicators to improve SSNB QoC.

The indicators within this list support the measurement of facility-level effective coverage for SSNB or preterm/LBW newborns by contributing to direct (outcome-adjusted) and indirect (eg, quality-adjusted) coverage measures as illustrated in online supplemental material 18.^37^

Three aspirational indicator topics still require development; these include well-being scores for parent/family and health providers and the thrive rate by neonatal birthweight group. These topics remain in the list to illustrate the multidimensional nature of QoC and avoid the tendency to drop the topics that are most difficult to implement and measure. Similarly, the need for longitudinal or interconnected systems that allow for monitoring of loss-to-follow-up and survival is challenging but important to assess health systems functioning. Finally, our proposed output indicators specific to target group provision of care but measured at unit/ward level monthly include provision of ‘baby-days’ for phototherapy, CPAP and ‘togetherness’ are novel and designated as requiring additional research to explore measurability and usability (online supplemental material 17). Parsimony of indicators prioritised by global bodies including the WHO is highly desirable for indicator adoption.^10^,^13^ Lessons learnt emerging from their use can inform further prioritisation efforts.

### Strengths and limitations

A strength of our study was the stepwise process as recommended for global health quality indicator development.^14^ Wherever possible, we adapted existing indicators in global documents to align and strengthen measurement efforts. Our structured, iterative consultation process included a wide range of country, regional and global stakeholders, including an online consultation in three languages with 172 respondents, most of whom selected a high-burden mortality setting as their context of greatest experience. We achieved a balanced list of indicators and ensured that indicators contributing to survival (eg, oxygen) also have implications for reducing morbidity or thriving (eg, prevention of retinopathy of prematurity). Another strength is that among 27 indicators with metadata, 19 (70%) are measured at the individual patient level and 25 (92%) are recommended for collection using RHIS. This supports the availability and use of real-time patient-level data to improve patient outcomes and guide and monitor the effect of quality improvement interventions.

Our work also has limitations. Because 24 indicators related to SSNB already exist in other global indicator guidance, the prioritised list could only be constrained so far. It was challenging to create a comprehensive short list from the diverse 78 domains of care and 578 measures of quality.^9^ However, we believe that being able to put forth an initial list that includes 27 indicators that touch on 51 domains of care is an important first step. The three additional measurement areas requiring indicator development reflect the shifts towards patient-centred care and linkages between levels of care and follow-up postdischarge.

Despite efforts to involve parents of SSNB in prioritising quality indicators, only 2.3% of the online survey respondents selected ‘parent’ as their primary role. For implementation, it will be important to incorporate caregiver perspectives into the indicators to be developed for infant-centred and family-centred care. Further, we need to consider alternate, more local ways of gathering input on what measurement should be prioritised. Given the wide range of stakeholders and experts consulted, there were challenges in reaching a consensus for the final list. We acknowledge this complexity and the importance of testing and experience in use to understand enablers of data collection and use. This information will contribute to revising these prioritised indicators as new information becomes available.

## Conclusion

We have developed these prioritised indicators to support the continued improvement of QoC for SSNB to ‘indicate’ critical gaps in care across the WHO QoC framework to accelerate progress towards globally agreed targets. The usefulness of these measures, however, relies on the ability to consistently collect accurate data for their calculations. Prioritised indicators may become increasingly valuable as the data are collected and used, improving data quality to address limits in data utility while improving care processes and outcomes. While data validity currently limits robust data-informed decision-making in many settings, data use may improve data quality.^38^ Systems and populations are dynamic; the indicators prioritised for measurement will have to reflect the contexts and needs in diverse settings. This list will benefit from field testing and implementation experience to improve on prioritisation, definitions, information sources and how well they drive improvement in QoC.

In many settings, investment is urgently needed to improve routine data, including standardisation and improvement of paper-based systems (patient notes and registers) as the foundation of digital systems, to avoid digitisation creating ‘too much poor-quality data too soon’.^39^

Identifying these prioritised indicators illustrates the ongoing commitment to comparable measurement to contribute to improving QoC across diverse settings from the first month of life and linking to the continuum of care and life course quality measurement. ‘What gets measured better gets done better’ encourages efforts to standardise and prioritise indicator measurement for quality improvement.^40^ Data is not an end in itself, and the data from these proposed QoC indicators need to be used to support health system strengthening, bringing an increased visibility for political commitment, vision and national plans to realise the right of every SSNB to survive and thrive.

## Supplementary material

10.1136/bmjopen-2025-100338 online supplemental file 1

## Data Availability

Data are available from the corresponding author upon reasonable request.
