# Supplementary material for: Global prioritised indicators for measuring WHO’s quality-of-care standards for small and/or sick newborns in health facilities: development, global consultation and expert consensus
Source: BMJ Open. 2025 Oct 20;15(10):e100338. doi: 10.1136/bmjopen-2025-100338 (PMC12542562; doi:10.1136/bmjopen-2025-100338)
Supplement: online supplemental file 1 [file bmjopen-15-10-s001.pdf]

BMJ Open – Original Research

Supplementary material for:

Global prioritised indicators for measuring WHO's quality-of-care standards for small and/or sick newborns in health facilities: development, global consultation, and expert consensus

Louise T Day, Lara M E Vaz, Katherine E A Semrau, Sarah Moxon, Susan Niermeyer, Neena Khadka, Tamar Chitashvili, Gregory C Valentine, Mary Drake, Danielle E Y Ehret, Ashley Sheffel, Emma Sacks, Leah Greenspan, Theresa R Shaver, Lily Kak, Tedbabe Degefie Hailegebriel, Gagan Gupta, Kathleen Hill, Debra Jackson

Contents

Supplemental Material 1: Composition of the SSNB QoC Indicators at establishment of the Technical Working Group 5

Supplemental Material 2: Mapping existing indicator lists for small and sick newborns 8

Supplemental Material 3: SSNBB core quality indicator set – general and SSNBB care related criteria 9

Supplemental Material 4: SSNB core quality indicator scoring tool 11

Supplemental Material 5: SSNB core quality indicator scoring tool – general indicator criteria 12

Supplemental Material 6: SSNB core quality indicator scoring tool - SSNB care related criteria 14

Supplemental Material 7: Linking existing indicators with quality measures not found in standards document 17

Supplemental Material 8: Proposed SSNB core quality indicator set online consultation (English) 18

Recruitment Email 18

Online Survey Page 1 – Instructions [in survey] 19

Online Survey Page 3 – Background information Document [Downloadable] 21

Online Survey Page 4 – Care for small or sick newborns [in survey] 26

Online Survey Page 5 – Small or sick newborns in the data pyramid [in survey] 27

Online Survey Page 6 – Purpose and audience of core indicator set [in survey] 28

Online Survey Page 7 - Consent - Participant Information Sheet [in survey] 29

Online Survey Page 7 - Consent Form 32

Online Survey Section 2: Page 8 - Respondent Background 33

Online Survey Section 3: Proposed set of small or sick (SSNB) quality indicators 35

Section 3.1 - Proposed SSNB CORE quality Indicators - already prioritized for SSNB in existing global CORE indicator lists 35

Section 3.2 - Proposed SSNB CORE quality indicators - based on adaptation of existing prioritized global maternal, newborn and paediatric CORE health indicator recommendations 36

Section 3.3 - Proposed SSNB CORE quality indicators – based on selected priority SSNB quality standards that are not represented in existing global indicator lists/ recommendations 37

Section 3.4 - Proposed SSNB OPTIONAL quality indicators – based on selected priority SSNB quality standards/statements that are not represented in existing global newborn indicator lists/recommendations. 38

Section 3.5 – Summary questions 39

Online Survey Section 4: More Information 40

Online Survey - Develop Core Indicator set for quality of care for Small or Sick Newborns Online Global Consultation 2022 - References for Section 3.1 and 3.2 ... 41

Supplemental Material 9: Online consultation – respondent characteristics ... 42

Supplemental Material 10: Online consultation – frequency of selected country ... 43

Supplemental Material 11: Online consultation – results ... 45

SSNB Indicator perceived usefulness ... 45

Core/Optional SSNB quality indicator selection ... 46

SSNB Indicator perceived feasibility to measure ... 48

Supplemental Material 12: Combined Ranking Useful/ Core/ Measurable ... 50

Supplemental Material 13: Prioritized indicators to measure quality of care for small and/or sick newborns (SSNB), metadata, online survey ranking and rationale for inclusion ... 51

Linked to manuscript Table 1 ... 51

Supplemental Material 14: Reporting standards for guideline-based performance measure development and re-evaluation[20] ... 68

Supplemental Material 15: Proposed parent survey indicators to include in an infant and family-centred newborn care score for neonatal unit, covering standards 1 to 8. Score requires defining and context validation (n=16) ... 71

Supplemental Material 16: Proposed provider survey indicators to include in a person-centred provider care score for neonatal unit, covering standards 1 to 8. Score requires defining and context validation (n=9) ... 72

Supplemental Material 17: Proposed Additional SSNB quality Indicators – covering standards 1 to 8 (n=8) ... 73

Supplemental Material 18: Proposed facility effective coverage cascade for SSNB ... 74

References ... 75

Table of figures

Supplementary Figure 1: SSNB indicators identified in existing WHO lists: SSNB specific (S1-S12) potentially adoptable (light pink) and SSNB relevant (R1-R12) potentially adaptable (dark pink). 8

Supplementary figure 2: Inpatient small and sick newborn care within the continuum of care 10

Supplementary figure 3: Health system responses for SSNB by level of care.. 16

Supplementary figure 4:Existing recommended indicators not identified in SSNB standards document 17

Supplementary figure 5: Potential 52 SSNB core quality indicators ranked by usefullness, online survey consultation, N =172 45

Supplementary figure 6: Potential 52 Core Quality indicators ranked by selection as core/ optional/ neither, online survey consultation, N=172.. 46

Supplementary figure 7: Potential 52 SSNB Core Quality indicators ranked by selection as neither/ optional/ core, online survey consultation N=172... 47

Supplementary figure 8: Potential 52 SSN Core Quality indicators ranked by estimation of number of years to possible measurement "2 years" to longer, online survey consultation, N=172 48

Supplementary figure 9: SSN Core Quality indicators ranked by estimation of number of years to possible measurement ranking from "don't know" to longer, online survey consultation, N=172....................................... 49

Table of tables

Supplementary Table 1: Combined scoring criteria for general indicator and SSNB quality standards ....**Error! Bookmark not defined.**

Supplementary Table 2: Supplementary Table 2: SSNB Core Indicator Online Survey respondent background characteristics 42

Supplementary Table 3: Prioritized indicators to measure quality-of-care for small and/or sick newborns (SSNB) categorized by WHO quality domain standards 1-8, indicator type (input, process/ output, outcome), context of care and online survey ranking A) All SSNB cared for in all health facility wards (labour & delivery ward, postnatal ward, KMC ward, neonatal wards/units) (n=10 indicators) B) Subset of SSNB admitted to neonatal wards/units only: defined (n=17 indicators), needing definitional work (n=3 indicators)... 51

Supplemental Material 1: Composition of the SSNB QoC Indicators at establishment of the Technical Working Group

Name	Organization	Job Title	Training, area of expertise, technical advisory group membership
Tamar Chitashvili	JSI (John Snow Inc)	Technical Director, Maternal and Newborn Health	Global Health, Paediatrics, Obstetrics & Gynaecology, Non-communicable diseases, Integrated Primary Care, Quality Improvement and Measurement.<br>Selected memberships on measurement and QoC space<br>• WHO's Life Course Quality of Care Measurement TWG<br>• WHO's Technical Advisory Group of the Mother and Newborn Information for Tracking Outcomes and Results (MoNITOR)<br>• WHO B1 Working Group on Life course Measurement; WHO Life course Network<br>• The Global Action for Measurement of Adolescent health (GAMA)<br>• Global Neonatal Advisory Committee, American Academy of Pediatrics<br>• Quality and Safety TWG at International Paediatric Association
Louise Tina Day,	LSHTM	Assistant Professor	Paediatrics, Obstetrics & Gynaecology, service delivery and management information systems design and implementation, quality improvement.
Ayesha De Costa	WHO, HQ	Scientist	Medical Doctor. Global Public Health
Theresa Diaz	WHO, HQ	Unit Head Epidemiology, Monitoring and Evaluation	Medical Doctor. Global Public Health
Mary Drake	Jhpiego	Senior Measurement, Evaluation and Learning Advisor	Nursing, global public health, monitoring & evaluation, quality-of-care measurement, quality improvement, maternal newborn health.
Danielle E Y Ehret	Vermont Oxford Network, University of Vermont Larner College of Medicine	Chief Medical Officer, Director of Global Health, Asfaw Yemiru Green and Gold Professor of Global Health, Professor of Pediatrics	Neonatology, quality improvement, neonatal network development across resource settings
Leah Greenspan	USAID	Senior Newborn Advisor Neonatologist	Neonatology, Public health

Name	Organization	Job Title	Training, area of expertise, technical advisory group membership
Gagan Gupta	UNICEF, HQ	Senior Advisor and Team Lead – Maternal and Newborn Health UNICEF Health Section	Paediatrics, Public health
Tedbabe Degefie Hailegebriel	UNICEF, HQ	Senior Advisor and Team Lead – Maternal and Newborn Health UNICEF Health Section	Paediatrics, Public health
Kathleen Hill	Jhpiego	Senior Technical Advisor for Quality. MOMENTUM Country and Global Leadership	Family Medicine, Global Health, Quality of care
Debra Jackson	LSHTM & UWC	Professor	MNCH Metrics, neonatal nursing, quality of care *and* MONITOR member
Lily Kak	USAID	Neonatal Team Lead	Global Public Health
Neena Khadka	Save the Children	Newborn Health Focal Point, MOMENTUM Country and Global Leadership	Neonatology, Global Public Health
Susan Niermeyer	University of Colorado	Professor emerita of paediatrics<br><br>Consultant, Newborn health	Paediatrics, neonatal-perinatal medicine, neonatal resuscitation, perinatal epidemiology, professional education
Allisyn Carol Moran	WHO, HQ	Unit Head Maternal Health	Global Public Health
Sarah Moxon	LSHTM	Assistant Professor Department of Infectious Disease Epidemiology	Nursing, child health, newborn health, health systems
Melinda Munos	JHSPH	Assistant Professor, Department of International Health	program evaluation, measurement methods, maternal, newborn, and child health
Moise Muzigaba	WHO, HQ	Technical Officer	Monitoring and Evaluation. Global Public Health.
Emma Sacks	JHSPH	Associate Faculty, Department of International Health	Global public health, neonatal health, respectful maternal and newborn care, perinatal epidemiology, measurement methods, quality improvement. WHO Advisory group memberships: Life course quality metrics; Alliance for Health Policy and Systems Research
Katherine Semrau	Ariadne Labs & Harvard Medical School	Director, BetterBirth Program and Associate Professor, Department of Medicine	Maternal Newborn epidemiology, intervention design, & quality improvement *and* MONITOR member
Theresa R Shaver	USAID	Nurse midwife, Senior Maternal and Newborn Health Advisor	Maternal and newborn health, policy, public health

Name	Organization	Job Title	Training, area of expertise, technical advisory group membership
Ashley Sheffel	JHSPH	Assistant Scientist, Department of International Health	quality-of-care, maternal and newborn health, monitoring and evaluation, measurement methods *and* MONITOR member
Gregory C Valentine	University of Washington	Department of Pediatrics, Division of Neonatology	Neonatal-Perinatal medicine, database development, maternal & child health, improving quality-of-care in newborn units.
Lara M. E. Vaz	MOMENTUM Knowledge Accelerator	Senior Program Director and Technical Director	Behaviour change, implementation research, improving measurement, maternal and newborn health *and* MONITOR member

Supplemental Material 2: Mapping existing indicator lists for small and sick newborns

Supplementary Figure 1: SSNB indicators identified in existing WHO lists: SSNB specific (S1-S12) potentially adoptable (light pink) and SSNB relevant (R1-R12) potentially adaptable (dark pink).

Currently Recommended WHO Indicator lists by Year

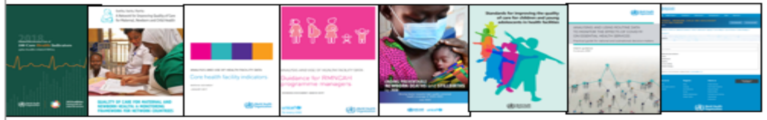

Indicator classification (impact, outcome, output, input)	MoNITOR indicator domain	Category	Indicator name short	2018<br>100 Core Health indicators	2018<br>Quality of Care network MNCH	2019<br>Core Health Facility Indicators	2019<br>Guidance for RMNCAH programme managers	2020<br>ENAP targets 2020-2025	2020<br>Quality of Care network Child	2021<br>WHO Covid-19 routine data	ongoing<br>Monitor indicator portal	Specific for SSN	Relevant for SSN
Impact	Mortality	Cause of death	Neonatal cause of death (%)								Core	S1	
			Neonatal cause of death in health facilities (%)								Core		
			Pre-discharge neonatal deaths by cause (count)		Common 3								
			Distribution of causes of death in health facilities (proportionate mortality)			Core							
			Case fatality rate (CFR) for major causes (%)			Core							
	Other health status	LBW	Low birth weight among livebirths (%)	ore						Indicator	Core	S2	
			Institutional low birth weight rate (%)				Core				Core		
		SGA	Prevalence of small for gestational age (%)								Core	S3	
		Preterm	Preterm birth rate among livebirths (%)				Additional				Optional	S4	
	Risk factors & behaviours		Institutional preterm birth rate (%)								Optional		
Outcome	Service coverage	KMC	Kangaroo Mother Care (%)		Common 13		Additional		Common 5	Indicator	Core	S5	
		Bag-mask-ventilation	Neonatal resuscitation Bag and Mask (%)				Additional				Core	S6	
		Sepsis treatment	Treatment for neonatal sepsis (%)				Additional				Core	S7	
		Sepsis treatment	Treatment for PSBI (%)						Common 4				
		Antenatal corticosteroids	Antenatal corticosteroid (%)								Optional	S8	
		Danger signs/illness feed	Counselling on danger signs and feeding during illness (Include 0-2m)						Common 19				R1
		Communication	Child/caregiver's understanding of the condition and treatment						Common 17				R2
		Communication	Participation and shared decision-making during care						Common 18				R3
		Respect	Awareness of childcare rights (Include 0-2m)						Common 20				R4
Output/ process	Service coverage	Newborn admission	Newborns admitted for inpatient care (number)							Indicator		S9	
	Service quality and safety	Death review	Death and QoC indicator data review (include neonate, stillbirth)						Common 16			S10	
	Service access & availability	Medical documentation	Completion of medical documentation						Common 15				R5
Input	Service access & availability	Medications	Stockouts of essential child health medications						Common 25				R6
			Availability of essential medicines/ commodities (UHC)			Core							
		Hygiene	Basic Hygiene Provision		Common 14						Optional		R7
		Sanitation	Basic sanitation available to women and families		Common 15						Optional		R8
		Level 2 inpatient unit	Level 2 inpatient unit for small or sick newborns					Target				S11	
		Resp - CPAP	Respiratory support small or sick newborns - CPAP					Target				S12	
		Health worker	Health worker density and distribution (UHC)/ 1000 population			Core							R9
		Health worker	Clinical mentorship and training						Common 24				R10
		Hospital	Hospital admission rate (inpatient utilization/ 100 population)			Core							R11
		Hospital	Service-specific availabiltiy (facilities/10,000 population)			Core							R12
Summary			Currently recommended SSN Indicators specific for SSN (potentially adoptable)	1	2	0	5	2	3	3	11		
			Currently recommended SSN Indicators relevant for SSN (potentially adaptable)	0	2	6	0	0	7	0	2		

Supplemental Material 3: SSNBB core quality indicator set – general and SSNBB care related criteria

General indicator set criteria:

An appropriate core set of indicators includes at least one indicator for each significant aspect of the program or project. In the context of this guideline-based indicator development, a balanced indicator set ideally would include:

- At least one indicator per quality standard.
- At least one indicator from structure (input), process and outcome (impact)

SSNBB quality standards related indicator set criteria:

- **Evidence based practices (Standard 1)**.
    - The continuum of care reflects the reality that SSNBB outcomes depends on pre-pregnancy and pregnancy care and beyond. The quality standards have excluded aspects of care (inputs/ coverage) that occur before birth (e.g. antenatal corticosteroids), during birth (e.g. preterm caesarean section rate). It is important to consider measuring indicators throughout the neonatal period day 0-28 and not only from day 1 of life because:
        - A **small** newborn is defined by birthweight, so specific care begins immediately after birth. Specific care for **sick** newborns starts when they develop a complication. A newborn may be **sick** immediately after birth or at any time during day 0-28 of life (e.g. normal birthweight newborn, well after birth develops sepsis on day 3)
        - With low coverage of high-quality care, neonatal mortality rates are highest on day 0 of life, high during days 1-7 and lower during days 8-28. As quality of increases, SSNB survive for longer and are exposed to other low quality care practices (e.g. hospital acquired infection or risk of retinopathy of prematurity due to treatment with high oxygen concentration).
    - "Survive" and "Thrive" are inevitably linked and SSNBB are at high risk especially for sub-optimal developmental and nutritional outcomes thus measurement considerations ideally should also consider long-term outcomes.

Supplementary figure 2: Inpatient small and sick newborn care within the continuum of care

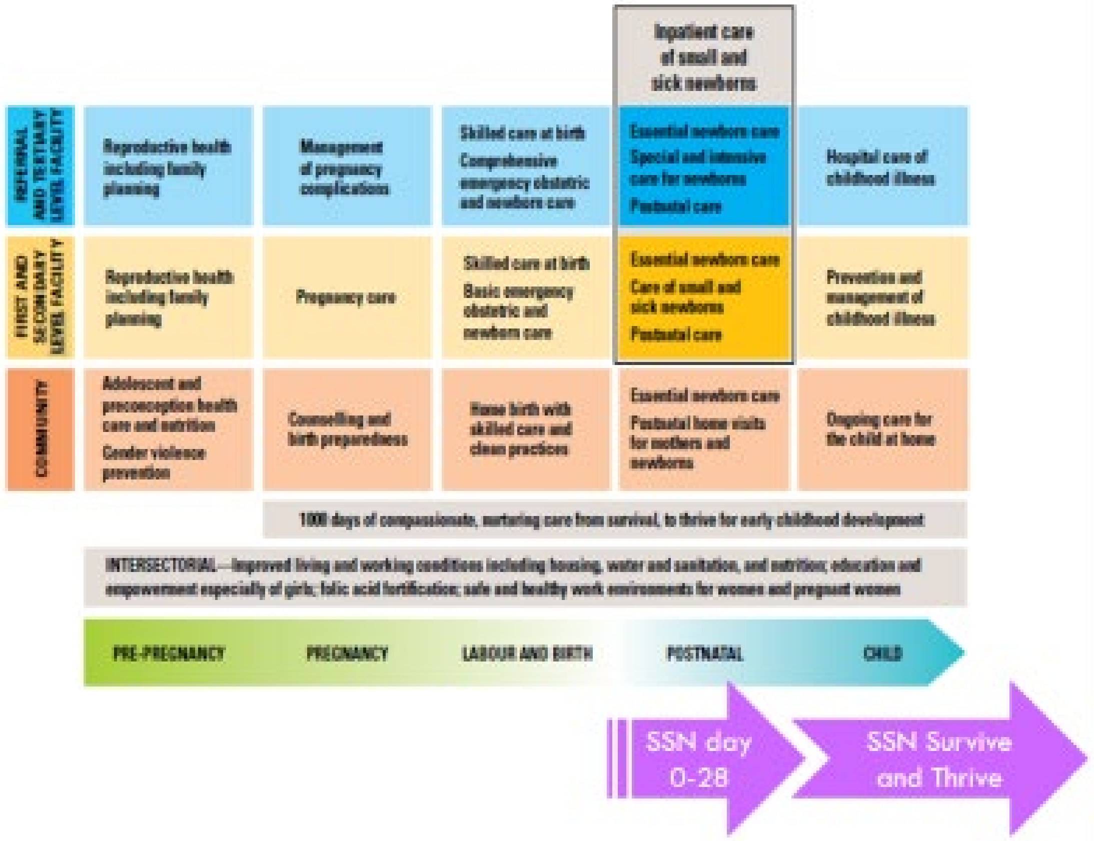

Supplemental Material 4: SSNB core quality indicator scoring tool

Supplementary Table 1: Combined scoring criteria for general indicator and SSNB quality standards

Criteria linking to:				My assessment is this SSN measure could be standardised into a SSN core quality indicator……	Score		
Facility measurement	Indicator Standard and assessment tool		Standards/ Monitoring Logic Framework		0	1	2
Action focused	1. Needed and useful			**…and it is clear what should be done to improve outcomes associated with this indicator**	No	Don't know	Yes
			Action focused	…and this indicator information would be clear to health facility manager what needs to be done to improve quality of care for SSN - the pathway is clear	No	Helpful	Essential
		1.6	Action focused	…and the indicator information would be used for decisions for SSN resource allocation at subnational/national level - the pathway is clear	No	Helpful	Essential
Important:				**…and the indicator and the data generated will make a relevant, significant contribution to determining how to respond to the problem effectively.**	No	Don't know	Yes
			Important	….and the indicator information would contribute to improvement in QoC with large impact in reducing in SSN mortality/morbidity	No	Helpful	Essential
			Important	….and the indicator information would contribute to improving QoC that is value for money	No	Don't know	Yes
		1.2	Important	…….and the indicator information would be important to quality of healthcare provided in the context of diverse cultural settings for SSN in LMIC	No	Don't know	Yes
			Important	…….and the indicator information would contribute to reducing country inequity for SSN/ families	No	Helpful	Essential
Simple and valued:	2. Technical Merit			**…and the people involved in the service can understand and value the indicator.**	No	Don't know	Yes
		2.2	Simple	…and the indicator would be a clear measure of progress in technical area	No	Don't know	Yes
		2.4	Info	…and the indicator would be sensitive to pick up change in performance	No	Don't know	Yes
		2.5	Info	…and the indicator would be reliable (consistency data collected repeatedly using same procedures same conditions)	No	Don't know	Yes
		2.6	Info	…and the indicator would be valid and specific (measures what intended to measure)	No	Don't know	Yes
			Info	…and the indicator would have low bias (effect during collection/interpretation leading to systematic error in one direction)	Yes	Don't know	No
			Simple	…and the indicator would be simple to interpret and use	composite	unclear	Simple
Operational	3. Fully defined			**…and the indicator is quantifiable; definitions precise, reference standards available and tested or could be developed.**	No	Don't know	Yes
		3.1-3.4	Measurable	…and the indicator could be fully defined within	>3 years	1-2 years	Now
			Measurable	…and the data elements for the indicator could be defined within	>3 years	1-2 years	Now
		3.5-2.6	Measurable	..and the data collection method for the indicator could be	special only	special / survey only	HMIS (special/survey)
		3.7-3.9	Measurable	…and the interpretation and use of the indicator could be set up within	>3 years	1-2 years	Now
Feasible:	4. Feasible to collect and analyse data			**…and it will be feasible to collect the data required for the indicator in the relevant setting.**	No	Don't know	Yes
		4.1	Measurable	…functioning systems and methods to collect could be set up within	>3 years	1-2 years	Now
		4.2	Measurable	…functioning systems and methods to interpret & use could be set up within	>3 years	1-2 years	Now
		4.3	Overlap	…this indicator would promotes interoperability in for other in M&E lists or systems eg. Logistics, HRH, CVRS, MPDSR	No	Don't know	Yes
		4.5	Measurable	…indicator is worth the cost and effort to measure	No	Don't know	Yes
			Limit burden	…the data needed to measure indicator is important for health worker clinical care who would be invested to collect high quality data with minimal burden	No	Helpful	Essential
			Sample size	…the prevalence for the indicator for SSN and their families would be	rarely	often	usually
			Sample Size	…and the indicator would be relevant for care given at level of care	Level 3 only	Level 1	Level 1 and 2
			Info	…the indicator would be a useful stratifier for inborn/outborns	No	Don't know	Yes
			Quality performance indicators	…and the type of indicator is	Input	Outcome	Process
			Referral	…and information from this indicator would identify intra-hospital transfer issues	No	Don't know	Yes
			Experience	…and the indicator measures infant and family-centred developmental care	No	Helpful	Essential
			Experience	…and information from this indicator would measure care across 0-28 days	No	Helpful	Essential
			Zero separation	…and information from this indicator would promote zero separation	No	Helpful	Essential
			Management	…and information from this indicator would identify health worker-patient care issues	No	Helpful	Essential
			Access	…and the indicator measure would have been affected by COVID collateral issues	No	Helpful	Essential

Supplemental Material 5: SSNB core quality indicator scoring tool – general indicator criteria

"Good" indicators are action-focused, important, measurable and simple.[9] The *Consultation on improving measurement of the quality of maternal, newborn and child care in health facilities*[44] scoring was based on these criteria with measurable divided into operational and feasible

**"Good indicator" criteria**	
Action focused	It is clear what should be done to improve outcomes associated with this indicator; Lead to action to improve quality of care
Important	The indicator and the data generated will make a relevant, significant contribution to determining how to respond to the problem effectively
(Measurable) Operational	The indicator is quantifiable; the definitions are precise, and reference standards are available and tested or could be developed
(Measurable) Feasible	It will be feasible to collect the data required for the indicator in the relevant setting
Simple and valued	The people involved in the service can understand and value the indicator

The Indicator Assessment tool (Indicator Standards: Operational guidelines for selecting indicators for the HIV response)[19] defines more detailed indicator assessment questions using 6 indicator standards: 1-5 for indicators and standard 6 for indicator sets

**Indicator Standards**	
Standard 1	The indicator is needed and useful
Standard 2	The indicator has technical merit
Standard 3	The indicator is fully defined
Standard 4	It is feasible to collect and analyse data for this indicator
Standard 5	The indicator has been field-tested or used in practice
Standard 6	The indicator set is coherent and balanced overall

An important principle of indicator development is to use existing indicators when at all possible. Indicators pertaining to SSNB are already represented to some extent in other newborn/ child indicator/target lists (Supplementary material 2) are the indicators specific for SSNBBs, either because they are included in the numerator (e.g. **small -** low birth weight) or received intervention/ care (e.g. **sick -** bag-mask-ventilation) and/ or were the true denominator or target group needing the intervention/ care e.g. **small –** 2000g for KMC). These will be cross-linked to *WHO SSNBB Standards document* to promote alignment.[8]

As the SSNB core indicators are intended for measurement in routine HMIS, consideration for frontline health-workers with dual responsibility to provide care and record/collect/aggregate data (e.g. in registers) will be used as a scoring criterion. The intention is to prioritize data that is also useful for real-time clinical decision making for investments in collection of high-quality data whilst limiting data collection burden.

To prioritize process indicators for change management, these will score higher than input and outcome indicators.

Indicator **measurement** is only valuable if the data are **valid, reliable,** and **non-biased**.[9] For SSNB core quality indicator measures that have not yet been assessed, "don't know" will be intentionally scored 1, between yes (2) and no (0).

**Indicator data credibility**	
Validity	The extent to which a measurement or test accurately measures what is intended to be.
Reliability	Consistency of the data when collected repeatedly using the same procedures and under the same circumstances.
Bias	Any effect during the collection or interpretation of information that leads to a systematic error in one direction.

The definition of indicator validity for global maternal and newborn health indicators has recently been advanced.[40]

**Indicator Concepts**	
Meaning	Conceptual clarity of intent and construct – what do we want the indicator to measure?
Meaningfulness	Usefulness and use of indicator – what can be achieved through its use?
Measurement	Method of obtaining an estimate – what measure is good enough?
Measurability	Feasibility, cost, acceptability – is it practical to measure this indicator?

Supplemental Material 6: SSNB core quality indicator scoring tool - SSNB care related criteria

The network monitoring logic model was applied to focus on SSNBB and to identify other relevant criteria for scoring and whether measures developed as indicators may act as tracers.[32]

Provision of care

- **Actionable information systems (Standard 2)**
    - Consider indicators that promote inter-operability between parallel information systems: health, logistics, human resources for health and Civil and Vital Registration systems, other surveillance, and response efforts (e.g. maternal and perinatal death surveillance and response (MPDSR)
    - Sample size adequacy – Criteria relating to the more common processes/ outcomes for SSNBB should be prioritized for indicator measurement to ensure data reflects performance level and not measurement "noise". ENAP coverage targets focus on level 2 care and core indicators for this subset can be considered. Admissions to level 2 unit could be a cohort representing a common denominator. Whether indicator measurement for level 2 units would be valid tracer measurement would require research.
    - SSNBB in facilities include both inborn (born in the facility) and outborns (transferred in from another facility or after homebirth). Outborn newborns are brought to the facility for two reasons – either they themselves are SSNB or their mother needs care. Ideally, stratifying indicator measurement by inborn/outborn is important to avoid inflating coverage of care using inborn delivery denominators. Outborn outcomes are important as a tracer measurement for access to care and quality of referral (e.g. acquired hypothermia during transfer)
    - Multiple births – twins and triplets can cause denominator count data confusion and ideally should be at baby level rather than women level. Multiple births will be over-represented among SSNB due to increased preterm birth rates.
- **Functioning referral systems (Standard 3). "**Referral" systems can be considered both between hospitals (inter-hospital) and within hospitals (intra-hospital).
    - In-utero transfers of women at high risk of delivering a small and sick newborn baby is the norm in high-income country (HIC) settings and much safer than transferring a SSNB baby after birth. During an inter-hospital referral, the "road" between the two hospitals is extremely risky either for a woman in early preterm labour or a SSNBB. It may cause separation between mother and baby if the mother is also unwell and cannot travel. It puts a financial burden on families to "live" far from home. When inter-hospital referral is needed after birth, HIC pathways are highly developed with designated transport teams accompanying the SSNBB and family after pre-referral treatment for stabilization. In low-income settings, inter-hospital referrals are very high risk and actually less common for logistical and financial reasons.

- Intra-hospital transfer between different wards in the hospital that provide targeted care for newborn and their families is experienced by almost all SSNB and their families, between labour and delivery, postnatal ward/ kangaroo mother care ward/ special care newborn ward (level 2)/ intensive care ward (level 3). The journey between these wards is also a risky time when SSNB can rapidly develop hypothermia and destabilize.

Experience of care

- **Effective Communication (Standard 4), Respect, protection and fulfilment of newborn rights and preservation of dignity (Standard 5), Emotional, psychological and developmental support (Standard 6)**
    - Critical to ensure infant- and family-centred developmental supportive care (positioning and interacting, optimizing nutrition, safeguarding sleep, minimizing stress and pain, protecting skin). Zero separation is highly valued. In facilities with level 2/3 newborn care, separation may be necessary immediately after birth to stabilize the baby (e.g. for (advanced) resuscitation, or early CPAP) while the mother receives early postnatal care (e.g. observation for post-partum haemorrhage, recovery after caesarean section anaesthesia), or if the baby's mother herself needs high dependency/ intensive care.
    - Continuity of care across the whole 28-day newborn period linked to the continuum of care

Management and organization

- **Competent, motivated, empathetic multidisciplinary SSNB skilled workforce (nurses and doctors) (Standard 7).**
    - Consider measurement that links the health worker-patient interface. Linkage to the recently published human resource strategies to improve newborn care in health facilities in LMIC.[6]
- **Essential physical resources for small and sick newborns available (Standard 8).**
    - Sub-national health managers need information from all levels of facilities caring for SSNBB. Measurement of coverage of care will vary according to recommended level 1/2/3 care practices/ interventions.

Access to care.

- Among all newborns, SSNB are a heterogenous and overlapping subset represented across all layers of the newborn pyramid. The sickest cohort of 30 million newborns need special or intensive care but ensuring all SSNBB receive the care they need to survive and thrive is vital and should be considered for indicator measurement (Supplementary figure 3).

Supplementary figure 3: Health system responses for SSNB by level of care

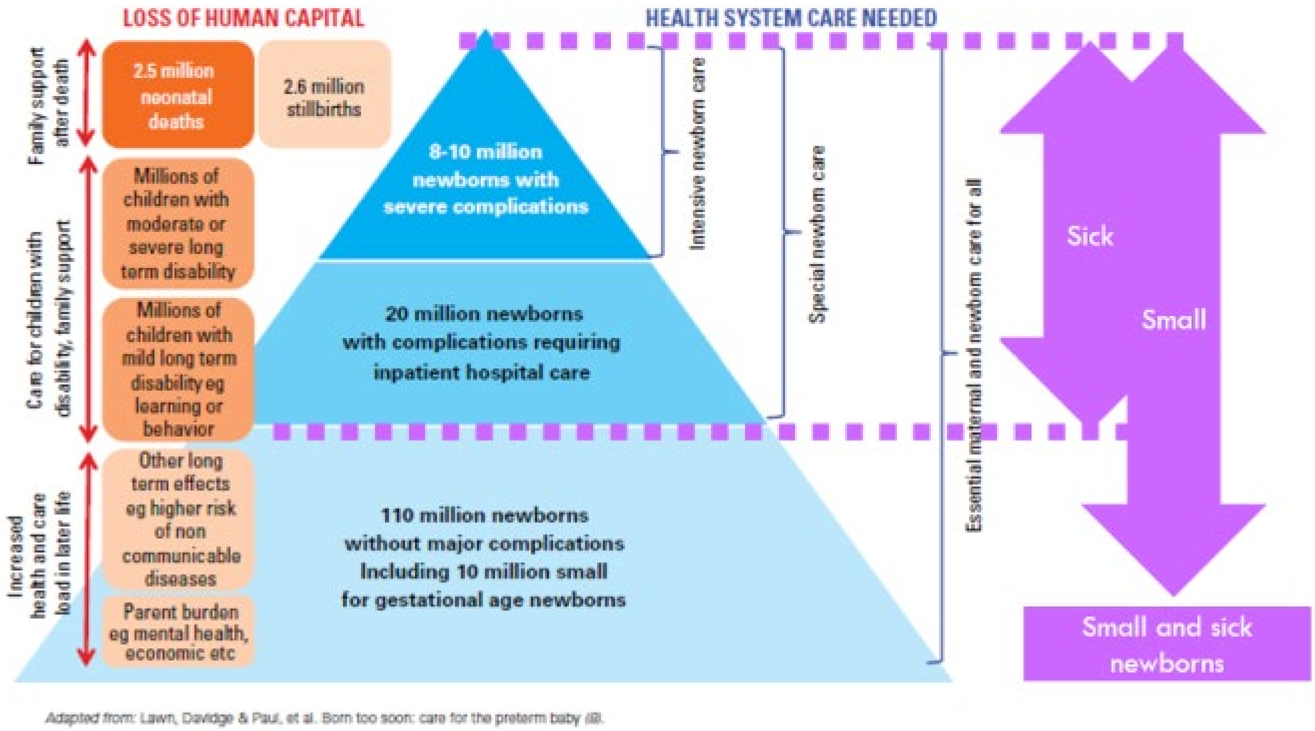

Supplemental Material 7: Linking existing indicators with quality measures not found in standards document

Supplementary figure 4:Existing recommended indicators not identified in SSNB standards document

MoNITOR Indicator type (input, output, outcome, impact)	MoNITOR indicator domain	Category	Overlap	Indicator name short
Impact	Other health status	LBW		Low birth weight among livebirths (%)
Impact	Other health status			Institutional low birth weight rate (%)
Impact	Other health status	SGA		Prevalence of small for gestational age (%)
Impact	Other health status	Preterm		Preterm birth rate among livebirths (%)
Impact	Risk factors & behaviours			Institutional preterm birth rate (%)
Outcome	Service coverage	Antenatal corticosteroids		Antenatal corticosteroid (%)
Input	Service access & availability	Level 2 inpatient unit		Level 2 inpatient unit for small or sick newborns
Input	Service access & availability	Resp - CPAP		Respiratory support small or sick newborns - CPAP
Input	Service access & availability	Health worker		Health worker density and distribution (UHC)/ 1000 population (+ newborn?)
Input	Service access & availability	Health worker		Clinical mentorship and training (+ newborn?)
Input	Service access & availability	Hospital		Hospital admission rate (inpatient utilization/ 100 population) (+ newborn?)
Input	Service access & availability	Hospital		Service-specific availabiltiy (facilities/10,000 population) - (+ newborn?)

Supplemental Material 8: Proposed SSNB core quality indicator set online consultation (English)

Recruitment Email

Dear Colleagues

Thank you for considering this invitation to take part in this online consultation:

Develop Core Indicator set for quality of care for Small or Sick Newborns

What is the purpose of the consultation?

The purpose is to gather feedback from partners and stakeholders like yourself on proposed core indicator sets for quality of care for small or sick newborns in high mortality and morbidity rate settings. The consultation is being coordinated by a technical working group organized by the World Health Organization (WHO) working with London School of Hygiene & Tropical Medicine (LSHTM).

Why have I been asked to take part?

You have been invited as a global partner and stakeholder for small or sick newborn indicator data collection and use. You can complete this consultation form either as an individual or on behalf of the organization for which you work.

What will I have to do?

When you access the online survey, you will be given access to a participant information sheet and a brief background document. You will be asked to review a set of proposed core quality indicators. We expect the survey to take 30 minutes of your time.

Further information and contact details

Thank you very much for taking time to read this request. We hope you will be able to contribute to this consultation.

Yours sincerely

Louise Tina Day, LSHTM

Moise Muzigaba, WHO Headquarters, Geneva

Online Survey Page 1 – Instructions [in survey]

The survey was created using Jisc Online surveys[22]

Please read the purpose of this consultation (page 2), background information (pages 3 to 6) and the participant information sheet. You will then need approximately 30 minutes to complete the survey. Thank you for taking the time to participate in this online consultation of core indicators for small or sick newborns.

Dr Moise Muzigaba, WHO Headquarters, Geneva
Dr Louise Tina Day, London School of Hygiene & Tropical Medicine

For further information, please contact Dr Louise Tina Day by email: Louise-Tina.Day@lshtm.ac.uk

Online Survey Section 1: Page 2 - Purpose of global Consultation

Standards for improving the quality of care for small and sick newborns in health facilities includes illustrative quality measures linked to quality statements. Using these quality standards as the organizing framework, WHO, in collaboration with other partners, seeks to prioritize and define a set of core small or sick newborn (SSNB) quality of care indicators. These will be added to the already-proposed set of core indicators for quality of care measurement in the *Quality of care for Maternal and Newborn Health: A monitoring framework.*

The primary purpose of the core SSNB quality of care measures is to support stakeholders across health system levels, and particularly at subnational level, to improve and monitor quality of care for SSNB in newborn care units (level 2 and level 3 units).

A working group led by WHO have selected a proposed set of quality indicators using agreed criteria (Figure 1)

Figure 1: Criteria for indicator selection

Action focused - it is clear what should be done to improve outcomes associated with this indicator
Important - the indicator and the data generated will make a relevant, significant contribution to determining how to respond to the problem effectively.
Simple and valued - the people involved in the service can understand and value the indicator.
Operational – the indicator is quantifiable, definitions and reference standards could be developed
Feasible – it will be feasible to collect data required for this indicator in the low- and middle-income setting

The purpose of this consultation is to gather feedback from partners and stakeholders on the proposed set of indicators. We are inviting the following partners and stakeholders of data collection and data use: WHO and UNICEF regional and country offices, parents, ministries of health, health workers/ professional organizations, implementing partners, technical working groups, researchers, independent experts. Thank you for forwarding the link to any partners you consider would be interested in providing feedback.

Online Survey Page 3 – Background information

A summary of background information is provided here, with more details in this background information document.

An estimated 2.4 million neonatal deaths occur globally each year and an additional 2 million babies are stillborn. If current trends persist, more than 60 countries will not meet the 2030 Sustainable Development Goal (SDG) for reducing neonatal mortality or the global targets for reducing stillbirths in the *Every Newborn Action Plan* (ENAP) and the *Global Strategy for Women, Children's and Adolescents' Health*.

Accelerating progress for small or sick newborns (SSNB) is critical as the subset of children with the highest morbidity and mortality. Global recognition merits establishing a more specific focused set of indicators to be used for action to improve quality of care.

Online Survey Page 3 – Background information Document [Downloadable]

Online stakeholder consultation on core indicator set for quality of care for small or sick newborns – 2022

This document provides more details for the summary given in Section 1 in the online survey.

Purpose of global consultation

Standards for improving the quality of care for small and sick newborns in health facilities[8] includes illustrative quality measures linked to quality statements. Using these quality standards as the organizing framework, WHO, in collaboration with other partners, seeks to prioritize and define a set of core small or sick newborn (SSNB) quality of care indicators to be added to the already-proposed set of core indicators for maternal, newborn and child (MNCH) quality-of-care measurement.[32] The primary purpose of the core SSNB quality--of-care measures is to support stakeholders across system levels, and particularly at subnational level, to improve and monitor quality of care for SSNB in newborn care units (level 2 and level 3 units).
A working group led by WHO have selected a proposed set of quality indicators using agreed criteria (Figure 1)

Figure 1: Criteria for indicator selection

- **Action focused** - it is clear what should be done to improve outcomes associated with this indicator.
- **Important** - the indicator and the data generated will make a relevant, significant contribution to determining how to respond to the problem effectively.
- **Simple and valued** - the people involved in the service can understand and value the indicator.
- **Operational** – the indicator is quantifiable, definitions and reference standards could be developed.
- **Feasible** – it will be feasible to collect data required for this indicator in the low- and middle-income setting.

The purpose of this consultation is to gather feedback from partners and stakeholders on the proposed set of indicators. We are inviting the following partners and stakeholders of data collection and data use: WHO and UNICEF regional and country offices, parents, ministries of health, health workers/ professional organizations, implementing partners, technical working groups, researchers, independent experts. Thank you for forwarding the link to any partners you consider would be interested in providing feedback.

Background information

An estimated 2.4 million neonatal deaths occur globally each year and an additional 2 million babies are stillborn. If current trends persist, more than 60 countries will not meet the 2030 Sustainable Development Goal (SDG) for reducing neonatal mortality or the global target for reducing stillbirths.[2]

A comprehensive, multi-partner initiative, the *Every Newborn Action Plan* (ENAP), calls on all stakeholders to take action to improve access to services and the quality-of-care for all pregnant women and newborns.[1]quality-of-care ENAP sets out recommendations for countries on how to reduce mortality and morbidity as well as how to close gaps in equity, accompanied by specific goals for reducing mortality, coverage targets and milestones by 2030, with reviews in 2020 and 2025. In adopting ENAP as resolution WHA 67.10 at the World Health Assembly in 2014, 194 countries committed themselves to transform the recommendations into action. Subsequently, the SDGs and the *Global Strategy for Women, Children's and Adolescents' Health* were endorsed.[3] Both include the target for reducing newborn mortality, and the Global Strategy includes a specific target for reducing stillbirths.

WHO, in collaboration with ENAP partners, is leading the work on care for small and sick newborns. *Survive and Thrive: Transforming care for every small and sick newborn* was published in 2019[5]. Policy and clinical guidance on management of small and sick newborns, including *Standards for improving the quality of care for small and sick newborns in health facilities*[8], a *Roadmap on human resources policy options for improving neonatal services* [6], and an *updated Charter on Respectful Maternity Care* that includes newborn rights, [7] were published in 2020.

Global recognition regarding the critical importance of accelerating progress for SSNB merits establishing a more specific small indicator set focused on SSNB as the subset of newborns and children with the highest morbidity and mortality. ENAP progress reports highlight more data are urgently needed to track progress to meet globally agreed targets.[45]

Care for small or sick newborns

Small or sick newborns (SSNB) are a group of high-risk babies: **small newborns** weigh <2500g at birth (low birth weight including preterm) and **sick newborns** have any medical or surgical condition. Nearly all newborns who die are small or sick and WHO Global Health Estimates show >40% of under-five mortality are newborns with SSNB a leading contributor to of disability-adjusted life-years (DALYS).[4] To Survive and Thrive, SSNB typically require inpatient hospital treatment including multiple care practices and interventions.[5]

The *Standards for improving the quality of care for small and sick newborns in health facilities* (hereafter "*WHO SSNB Standards document*" includes a framework describing eight domains [8] (Figure 2). Each domain has one linked standard describing what is expected to ensure high-quality care.[8].

Figure 2: Framework for improving the quality of care for small and sick newborns

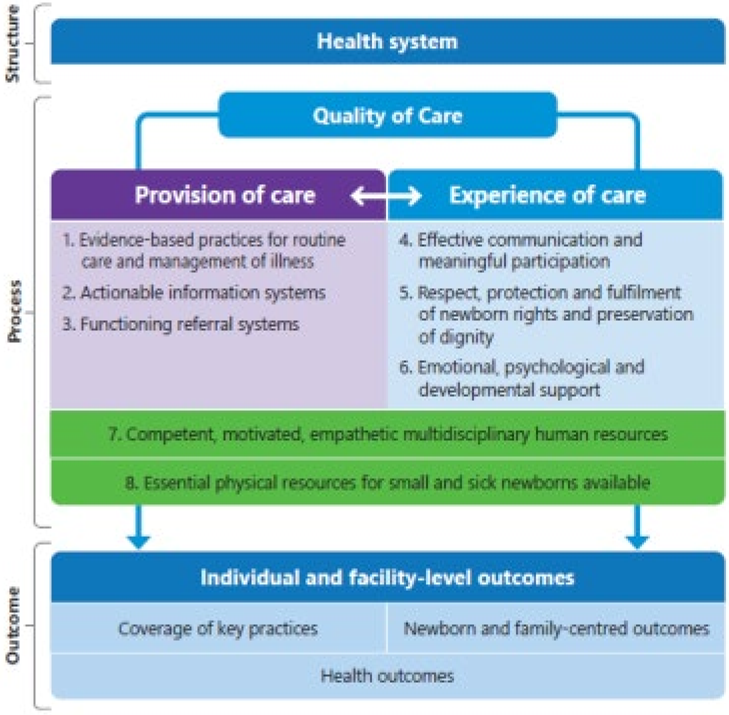

Small or sick indicators in the data pyramid

Indicators are standardized quantitative measures to provide critical information to monitor performance, measure achievement and determine accountability (Table 1). They form an important part of the actionable information system (quality standard 2) give a sign or signal (literally "indicate") something which allows comparisons over time and geographies. Indicators have defined components (title, definition, purpose, rationale, method of measurement, numerator, denominator, data collection method/ tool/ frequency, data disaggregation, guidelines to interpret and use data).[9]

Table 1: Indicator purposes

**Indicator Purpose**	**Details**
Monitor performance	Effective or efficient operation of an activity / project/ programme
Measure achievement	Successful accomplishments of an activity / project/ programme
Determine accountability	Responsibility for the performance and/ or achievements of activity / project/ programme

Indicators connect on the data pyramid broadly at three levels (Figure 3).

A) Facility quality improvement indicators (catalogue)
These standardized quality improvement measures are closely linked to the agreed SSNB standards-statements-measures. They are sensitive to change, closely linked to performance and used by QI facility teams to support rapid improvement of specific processes and health outcomes.

B) (Sub)-national priority performance indicators (core)
A smaller number of priority performance indicators aggregated from facility level indicators are strategically useful by district managers to monitor performance, measure achievement and determine accountability. These indicators are sensitive to change linked to changes in quality.

C) Global common indicators (core)
A few indicators are used for accountability for globally agreed goals (e.g. Sustainable Development Goals SDG) and typically change slowly.

Figure 3: Indicator pyramid - levels of indicators

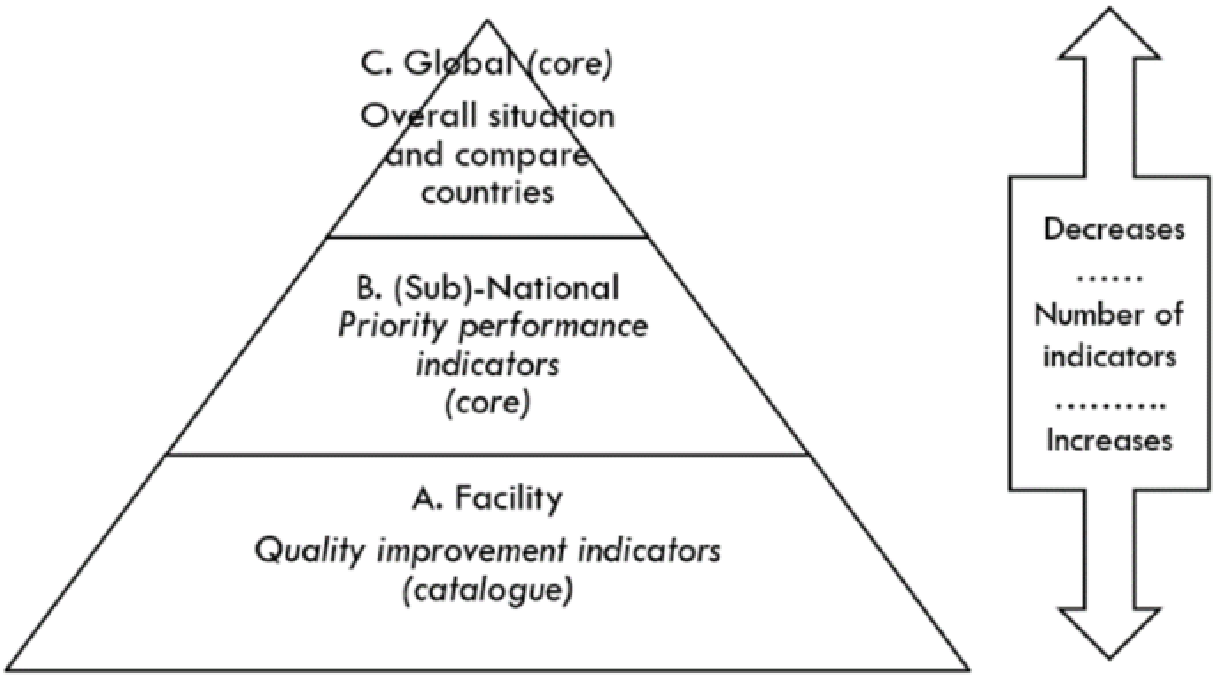

Purpose and Audience of core small or sick indicators

WHO are leading this first global effort to establish a set of priority core SSNB indicators.

Priority performance indicators (Figure 3) are envisaged, to enable change management for an audience of health workers, facility managers and sub-national health managers because ""What gets measured better gets done better.[40] Measurement through the Routine Health Information Systems (RHIS) is desirable to facilitate regular (yearly/ quarterly/ monthly) data availability for use, however noting that the domain of experience of care necessitates client interviews.

Methodology
A guideline-based quality indicator development methodology has been used with several steps:

- **Extraction of measures for potential (SSNB) quality indicators** – two extractors used scoring criteria to pre-select a sub-set of the 578 quality measures from the SSNB standards document for consideration for potential indicator development.

- **Selection of quality SSNB indicators proposed for development** – a small working group formed by WHO to purposively include clinical health workers, measurement and quality improvement experts used scoring criteria (Figure 1) independently and by discussion for consensus to select a smaller set of proposed core quality indicators.

Throughout, the working group has sought to:

- strengthen existing indicator measurement by adopting or adapting indicators from existing key/core lists.

- add only a small number of extra indicators to reduce measurement burden in high mortality/ morbidity settings.

Stakeholder review of proposed set of SSNB quality indicators:
The topic of this consultation is an invitation for your review to:

- **Consider if the proposed set of SSNB quality indicators are:**
  - **balanced across the whole newborn period 0-28 days?**
  - **balanced for newborns to both survive and thrive?**
- **Listen to your recommendations to include or exclude specific proposed quality indicators from the proposed core set.**
- **Estimate the timeframe within which the proposed quality indicators could be measured in the geographical settings where you work.**

You will also have the opportunity to add optional comments.

Online Survey Page 4 – Care for small or sick newborns [in survey]

Small or sick newborns (SSNB) are a group of high-risk babies: **small newborns** weigh <2500g at birth (low birth weight including preterm) and **sick newborns** have any medical or surgical condition. To Survive and Thrive, SSNB typically require inpatient hospital treatment including multiple care practices and interventions.

The *Standards for improving the quality of care for small and sick newborns in health facilities* (hereafter "*SSNB Standards document*" includes a framework describing eight domains (Figure 2). Each domain has one linked standard describing what is expected to ensure high-quality care.

Figure 2: Framework for improving the quality of care for small and sick newborns

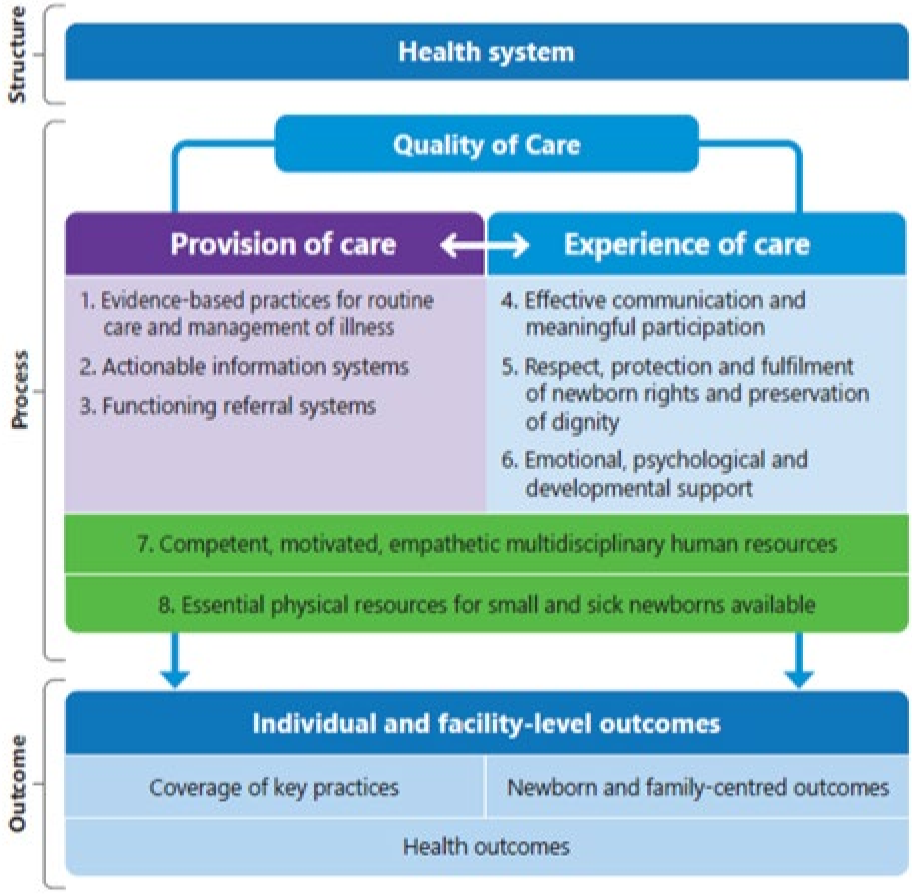

Online Survey Page 5 – Small or sick newborns in the data pyramid [in survey]

Indicators are standardized quantitative measures to provide critical information to monitor performance, measure achievement and determine accountability. They "indicate" something which allows comparisons over time and geographies.

Indicators connect on the data pyramid broadly at three levels (Figure 3).

A) Facility quality improvement indicators (catalogue)
Closely linked to performance and used by quality improvement facility teams to support rapid improvement of specific processes and health outcomes.

B) (Sub)-national priority performance indicators (core)
A smaller number aggregated from facility level indicators for strategic use by sub-nationally and sensitive to change linked to changes in quality.

C) Global common indicators (core)
A few indicators are used for accountability for globally agreed goals (e.g. Sustainable Development Goals SDG) and typically change slowly.

Figure 3: Indicator pyramid - levels of indicators

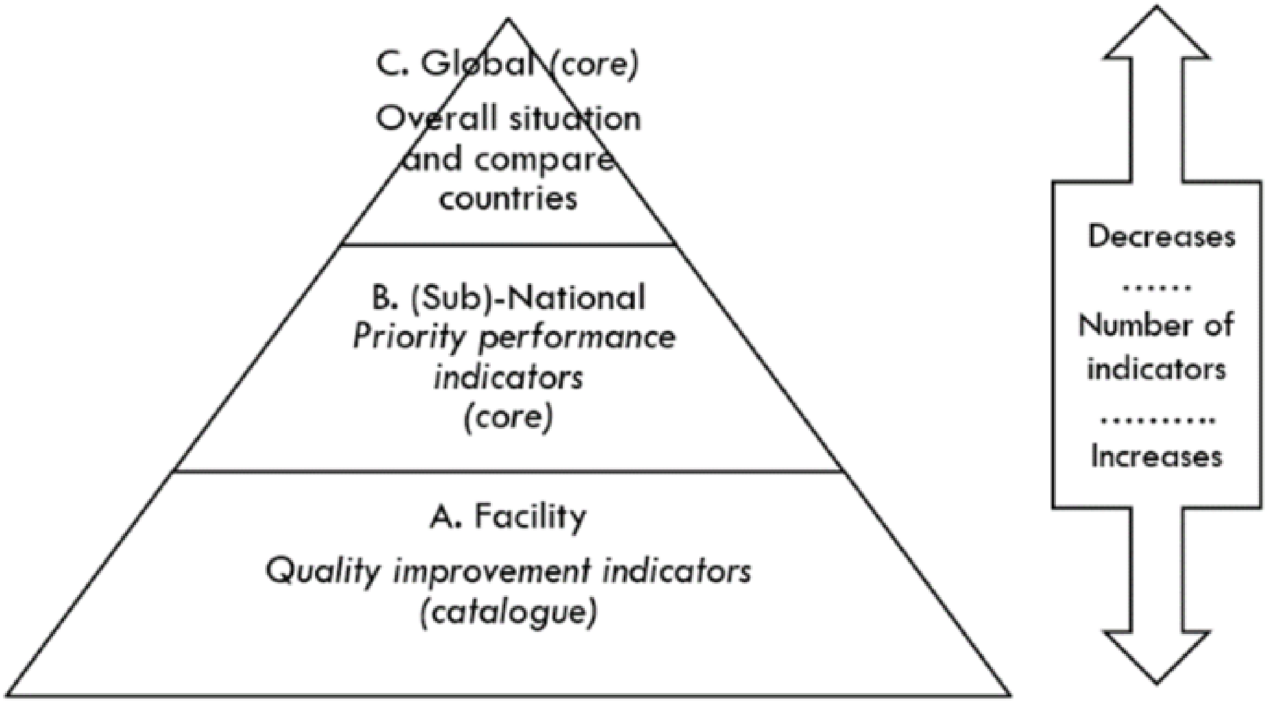

Online Survey Page 6 – Purpose and audience of core indicator set [in survey]

WHO are leading this first global effort to establish a set of priority core indicators for quality of care for small or sick newborns for countries with current high mortality and morbidity rates.

Priority performance indicators (B in Figure 3 above) are envisaged, to enable change management for an audience of health workers, facility managers and sub-national health managers because "What gets measured better gets done better". Measurement through the Routine Health Information Systems (RHIS) is desirable to facilitate regular (yearly/ quarterly/ monthly) data availability for use, however noting that the domain of experience of care necessitates client interviews.

Methodology

A guideline-based quality indicator development methodology has used the WHO SSNB Standards document, throughout seeking to:

- strengthen existing indicator measurement by adopting or adapting indicators from existing key/core lists.
- add only a very limited number of extra core indicators to avoid unnecessary additional measurement burden
- prioritize process/output indicators where possible ahead of input indicators.

Stakeholder review of proposed set of SSNB quality indicators:

The topic of this consultation is an invitation for your review to:

- **Consider if the proposed set of SSNB quality indicators are:**
    - **balanced across the whole newborn period 0-28 days?**
    - **balanced for newborns to both survive and thrive?**
- **Listen to your recommendations to include or exclude specific proposed quality indicators from the proposed core set**
- **Estimate the timeframe within which the proposed quality indicators could be measured in the geographical settings where you work**

You will also have the opportunity to add optional comments.

Online Survey Page 7 - Consent - Participant Information Sheet [in survey]

Please read the participant information sheet

Title of Consultation: *Develop Core Indicator set for quality of care for Small or Sick Newborns*

Introduction

Thank you for considering this invitation to take part in our online consultation. Participation is entirely up to you and before you decide, we would like you to understand why the consultation is being done and what it would involve for you. Ask questions if anything you read is not clear or you would like more information. Please feel free to talk to others about the consultation if you wish. This first part of the participant information sheet tells you the purpose of the study then we give you more detailed information about the consultation.

What is the purpose of the consultation?

The purpose is to gather feedback from partners and stakeholders like yourself on proposed core indicator sets for quality of care for small or sick newborns in low-and middle-income countries. The consultation is being coordinated by London School of Hygiene & Tropical Medicine (LSHTM) working with a working group at World Health Organization (WHO).

Why have I been asked to take part?

You have been invited as a global partner and stakeholder for small or sick newborn indicator data collection and use. You can complete this consultation form either as an individual or on behalf of the organization for which you work.

Do I have to take part?

No. It is up to you to decide to take part or not. If you agree to take part, we will ask for your consent and collect some background information about your profession, experience, country where you are working and email contact details so we can contact you for further information on the comments you submit. You can submit your responses anonymously if you prefer.

What will happen to me if I take part?

After you have read this information sheet we expect the online survey to take 15-30 minutes of your time.

What will I have to do?

You will be asked to read a brief background document explaining:

- *current context of measurement for small or sick newborns.*
- *criteria that have been used to select and form sets of these sets of additional indicators:*

You will be asked to review between 1-3 sets of quality indicators to assess whether the proposed indicator set is balanced (Strongly disagree/ Disagree/ neutral / Agree / Strongly Agree):

- *The small or sick newborn core quality indicator set is balanced across the whole newborn period 0-28 days.*
- *The small or sick newborn core quality indicator set is balanced for both newborns surviving and thriving.*

You can send us optional comments regarding any of the proposed indicators regarding 5 criteria: Action focused, Important, Simple and valued, Operational, Feasible

What are the possible risks and disadvantages?

There are no direct risks to participating but it will take approximately 15-30 minutes of your time. If you choose to give your contact details, then you may be contacted to follow up to your comments or for a consultation meeting.

What are the possible benefits?

The information we get from the consultation will be used to revise the indicator sets.

What if something goes wrong?

If you have a concern about any aspect of this consultation, you should email the researchers who will do their best to answer your questions louise-tina.day@lshtm.ac.uk. If you remain unhappy and wish to complain formally, you can do this by contacting Patricia Henley at rgio@lshtm.ac.uk or +44 (0) 20 7927 2626

Can I change my mind about taking part?

Yes. You can withdraw from the consultation at any time.

What will happen to information collected about me?

Your personal details, meaning your name and other identifiable information, will be kept safe in a secure database at LSHTM and only the researchers will have access to the data.

At the end of the project, all identifying information will be removed from the data which will be archived at LSHTM for 10 years in a secure back-up system or a data repository to be available on request for journal publications from this project or further analyses and journal publications by subsequent projects. Your personal information will not be included and there is no way that you can be identified.

What are your choices about how your information is used?

You can stop being part of the study at any time, without giving a reason and we will not include anything you have submitted in the consultation.

Where can you find out more about how your information is used?

You can find out more about how we use your information

- *At https://www.lshtm.ac.uk/files/research-participant-privacy-notice.pdf*
- *by asking one of the research team*
- *by sending an email to DPO@lshtm.ac.uk*

What will happen to the results of this study?

The study results will be published in a journal to share the learning from this consultation to improve measuring indicators for small or sick newborns can learn from them. Your personal information will not be included in the study report and there is no way that you can be identified from it.

Who is organizing and funding this study?

World Health Organization are the sponsor for this indicator prioritization process through a contract with the London School of Hygiene & Tropical Medicine who have full responsibility for the project including the collection, storage and analysis of your data, and will act as the Data Controller for the study. This means that we are responsible for looking after your information and using it properly.

Who has reviewed this study?

All research involving human participants is looked at by an independent group of people, called a Research Ethics Committee, to protect your interests. This study has been reviewed and given favorable opinion by The London School of Hygiene and Tropical Medicine Research Ethics Committee (26438).

Further information and contact details

Thank you very much for taking time to read this information sheet. If you think you will take part in the study, please read the background document and when you start the online survey, the first questions are for you to indicate your consent.

Online Survey Page 7 - Consent Form

Please answer each question

Title of Consultation: *Develop Core Indicator set for quality of care for Small or Sick Newborns*

Name of PI/Researcher responsible for project: Dr Louise Tina Day

**1. Statement**	**Select the radio button**
This survey aims to gather feedback from partners and stakeholders on proposed core indicator sets for quality of care for small or sick newborns in low- and middle-income countries. This study was approved by LSHTM Ethics Committee (ref 26438)<br>* required	I understand/confirm/agree
I have read the information sheet for the above-named study dated 25 July 2021 (version 1)<br>* required	I understand/confirm/agree
I understand that my consent is voluntary and that I am free to withdraw this consent at any time without giving any reason, information already collected will be destroyed<br>* required	I understand/confirm/agree
I understand that data collected during the consultation will be looked at by researchers from London School of Hygiene & Tropical Medicine and World Health Organization and I give permission for these individuals to have access to my survey responses.<br>* required	I understand/confirm/agree
I understand that data may be shared via a public data repository or by sharing directly with other researchers, and that I will not be identifiable from this information.<br>* required	I understand/confirm/agree
I agree to take part in the *Develop Core Indicator set for quality of care for Small or Sick Newborns* consultation<br>* required	I understand/confirm/agree
**2. Future contact questions:**	
a) I am willing to be contacted for further information on my responses submitted	Yes/ No
b) I would like to be informed of the results of the consultation	Yes/ No
If answers "no" to both a) or b) skip to start of survey<br>If answers "yes" to either a) or b) go to question c)	
**3. I answered "yes" to one of these questions I can be contacted on this email address:**	Text box for email address

Online Survey Section 2: Page 8 - Respondent Background

**Requires response*

4. *N*ame and surname

Please enter N/A if submitting without name and surname

5. Are you submitting on behalf of an individual or an organization?
Choose one of the following answers.

   - Individual

   - Organization

6. Name of organization. *Please enter N/A if submitting as an individual*

7. Please indicate from this list of stakeholders below the category/ categories that best describe you as a respondent. *Check any that apply**
   - WHO and UNICEF regional and country offices,
   - Parent or parents' organization,
   - ministries of health,
   - health worker,
   - professional organization,
   - implementing partner organization
   - Technical working groups,
   - research institution,
   - independent expert
   - Work in the public sector
   - Work in the private sector

7. Country where you are based.
Please select from the drop down the name of the country where you are based.

8. Geographic scope of your organization's work: *Check any that apply**

   - Global
   - Regional
   - National
   - Sub-national

9. Please select the SDG region(s) in which your organization performs its work: *Check any that apply**

- Sub-Saharan Africa
- Northern Africa and Western Asia
- Central and Southern Asia
- Eastern and South-Eastern Asia
- Latin America and the Caribbean
- Oceania
- Europe and Northern America

10. Please select your cadre *Check any that apply**

- Parent
- Midwife
- Nurse
- Medical Doctor
- Other medical professional
- Public Health
- Epidemiology
- Research
- Health management information specialist
- Other (please specify)

11a. Other (please specify

11. Please select your primary professional role with respect to newborn health:

- -Parent or representative of a parents' association
- -National, Regional or District MNH/MNCH Manager
- -National, Regional or District Health Information manager
- -Hospital Manager
- -Manager in a level 2 or level 3 Newborn Unit
- -Health worker in a in a level 2 or level 3 Newborn Unit
- -Public Health practitioner (implementing partner)
- -Researcher
- -Other (please specify)

12. Other primary professional role – please specify

Online Survey Section 3: Proposed set of small or sick (SSNB) quality indicators

Please select from the drop down one country you know well and use this context to answer questions in section 3.1, 3.2, 3.3 and 3.4

Section 3.1 - Proposed SSNB CORE quality Indicators - already prioritized for SSNB in existing global CORE indicator lists

13.

These indicators are already prioritised for small or sick newborns in existing global newborn health CORE indicator lists (references available in supplementary file Annex 4). We propose all these indicators to be automatically adopted for inclusion in the SSNB quality indicator CORE list to strengthen existing measurement. We would like to know your opinion and request you to kindly answer the questions below please

	My assessment is that for monitoring and improving quality of SSN care in the country specified above, this indicator will be: *				My assessment for this proposed indicator is to: *			My estimation is that measurement of this indicator in the country specified above would be possible within a timeframe of: *			
	very useful	somewhat useful	not useful	don't know	keep in core set	move to optional set	remove from core and optional sets	2 years	3 to 5 years	more than 5 years	don't know
(Standard 1) Pre-discharge neonatal mortality rate	○	○	○	○	○	○	○	○	○	○	○
(Standard 1) Neonatal cause of death in health facilities	○	○	○	○	○	○	○	○	○	○	○
(Standard 1) Institutional low birth weight rate	○	○	○	○	○	○	○	○	○	○	○
(Standard 1) Institutional preterm birth rate	○	○	○	○	○	○	○	○	○	○	○
(Standard 1) Small for gestational age prevalence	○	○	○	○	○	○	○	○	○	○	○
(Standard 1) Neonatal resuscitation bag-and-mask ventilation coverage (currently being defined)	○	○	○	○	○	○	○	○	○	○	○
(Standard 1) Antenatal corticosteroid coverage (currently being defined)	○	○	○	○	○	○	○	○	○	○	○
(Standard 1) Neonatal sepsis/ infection treatment coverage (currently being defined)	○	○	○	○	○	○	○	○	○	○	○
(Standard 1) Kangaroo Mother Care coverage (currently being defined)	○	○	○	○	○	○	○	○	○	○	○

Section 3.2 - Proposed SSNB CORE quality indicators - based on adaptation of existing prioritized global maternal, newborn and paediatric CORE health indicator recommendations

14.

These quality indicators are newly adapted for SSNB from paediatric or health facilities CORE quality indicator recommendations. We propose that these adapted indicators be considered for automatic inclusion in the SSNB quality indicator CORE list, to strengthen existing measurement. We would like to know your opinion and request that you kindly answer the questions below please.

	My assessment is that for monitoring and improving quality of SSN care in the country specified above, this indicator will be: *				My assessment for this proposed indicator is to: *			My estimation is that measurement of this indicator in the country specified above would be possible within a timeframe of: *				
	very useful	somewhat useful	not useful	don't know	keep in core set	move to optional set	remove from core and optional sets	2 years	3 to 5 years	more than 5 years	don't know	Optional comments
(Standard 1) % of carers of newborns admitted to the newborn unit who are aware of newborn danger signs, when to seek care and how to feed their baby during illness	○	○	○	○	○	○	○	○	○	○	○	T
(Standard 2) % of medical records of newborns admitted to the newborn unit with complete patient key information at admission assessment, discharge and follow-up plans	○	○	○	○	○	○	○	○	○	○	○	T
(Standard 2) % of newborn units that have conducted monthly neonatal Quality of Care indicator data review for the purpose of improving care	○	○	○	○	○	○	○	○	○	○	○	T
(Standard 4) % of carers of newborns admitted to the newborn unit who can describe the newborn's condition, home care and follow-up plan	○	○	○	○	○	○	○	○	○	○	○	T
(Standard 5) % of carers of newborns admitted to the newborn unit who reported being adequately informed about their child's rights to care	○	○	○	○	○	○	○	○	○	○	○	T
(Standard 5) % of carers of newborns admitted to the newborn unit who reported their newborn being mistreated during care	○	○	○	○	○	○	○	○	○	○	○	T
(Standard 6) % of carers of newborns admitted to the newborn unit who reported being satisfied with the decision-making process for care.	○	○	○	○	○	○	○	○	○	○	○	T
(Standard 6) % of carers of newborns admitted to the newborn unit who report being satisfied with ability to stay/room-in with their newborn during their admission at any time.	○	○	○	○	○	○	○	○	○	○	○	T
(Standard 7) Health workers with neonatal skills density and distribution (UHC) / 1000 population	○	○	○	○	○	○	○	○	○	○	○	T
(Standard 7) % of newborn health providers working on the newborn unit who reported receiving clinical Quality Improvement mentorship or training in the past 6 months	○	○	○	○	○	○	○	○	○	○	○	T
(Standard 8) % of newborn units with basic sanitation available for carers of newborns admitted to newborn unit	○	○	○	○	○	○	○	○	○	○	○	T
(Standard 8) % of newborn units which have functioning hand hygiene station at the entrance with water and soap available	○	○	○	○	○	○	○	○	○	○	○	T
(Standard 8) % of newborn units reporting no stockout of three essential tracer medicines in correct formulations (1. first-line injectable antibiotics, 2. phenobarbitone 3. caffeine (or other methylxanthines)) in a specified period	○	○	○	○	○	○	○	○	○	○	○	T

Section 3.3 - Proposed SSNB CORE quality indicators – based on selected priority SSNB quality standards that are not represented in existing global indicator lists/ recommendations

15. These quality indicators have been prioritized by the technical working group from the SSNB Standards document for inclusion in the CORE set. The criteria used to prioritize were: action-focused, important, simple and valued, operational and feasible (see Figure 1 in "Purpose of the consultation" section or background information document). These indicators are not currently in existing global newborn indicator CORE lists. We would like to know your opinion and request that you kindly answer the questions below please.

	My assessment is that for monitoring and improving quality of SSN care in the country specified above, this indicator will be: *				My assessment for this proposed indicator is to: *			My estimation is that measurement of this indicator in the country specified above would be possible within a timeframe of: *				
	very useful	somewhat useful	not useful	don't know	keep in core set	move to optional set	remove from core and optional sets	2 years	3 to 5 years	more than 5 years	don't know	Optional comments.
(Standard 1) % of newborns exclusively breast-milk fed (sucking, cup/ tube fed) at discharge from the newborn unit	○	○	○	○	○	○	○	○	○	○	○	
(Standard 1) % neonatal birth weight group survival rate at facility discharge after admission to the newborn unit	○	○	○	○	○	○	○	○	○	○	○	
(Standard 3) % of newborns with an admission temperature < 36.5 °C on admission to the newborn unit from other hospital ward or referral	○	○	○	○	○	○	○	○	○	○	○	
(Standard 5) % of newborns born in the health facility notified/ registered* to the appropriate civil authority (* = appropriate for setting)	○	○	○	○	○	○	○	○	○	○	○	
(Standard 5) % of newborn deaths and stillbirths in the health facility notified/ registered* to the appropriate civil authority (* = appropriate for setting)	○	○	○	○	○	○	○	○	○	○	○	
(Standard 8) % of newborn units with no stockout of safe oxygen delivery systems	○	○	○	○	○	○	○	○	○	○	○	
(Standard 8) % of districts/sub-national areas with one or more level 2 newborn units	○	○	○	○	○	○	○	○	○	○	○	

Section 3.4 - Proposed SSNB OPTIONAL quality indicators – based on selected priority SSNB quality standards/statements that are not represented in existing global newborn indicator lists/recommendations.

16.

These quality indicators have been prioritized by the working group from the SSNB Standards document for inclusion in the OPTIONAL set using the same criteria as section 3.3. We would like to know your opinion and request that you kindly answer the questions below please

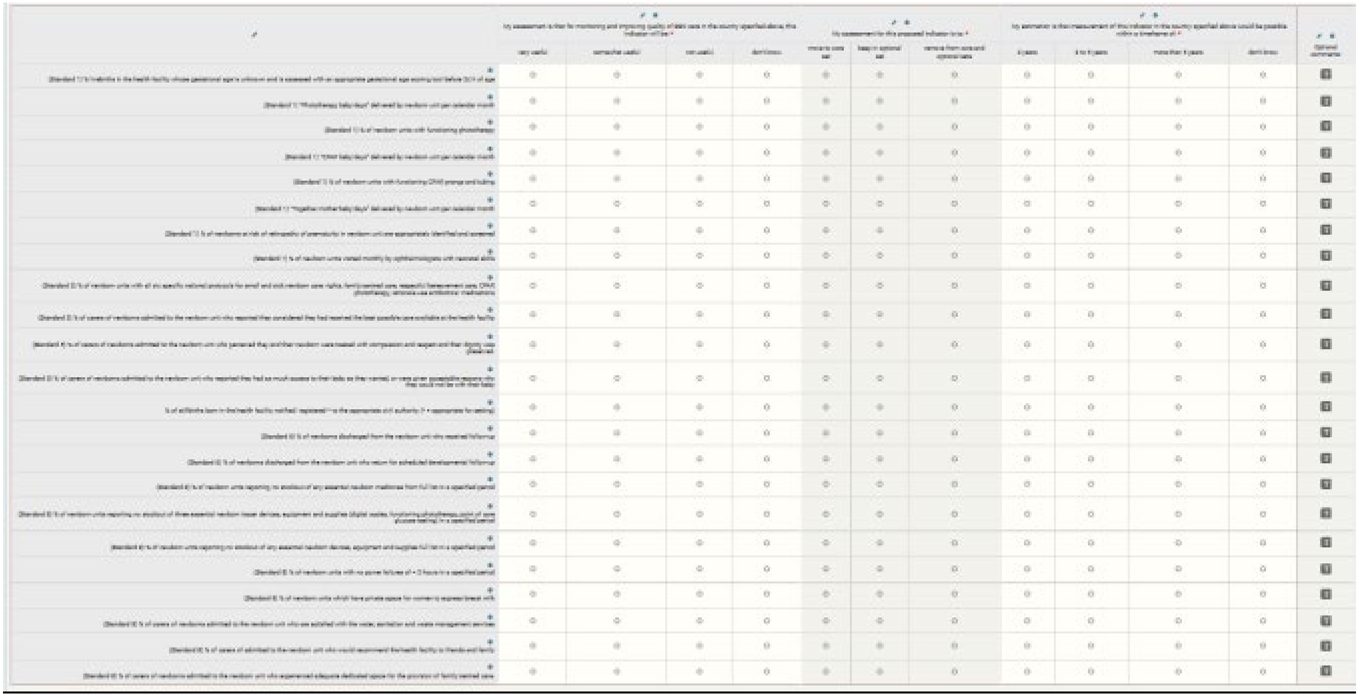

Section 3.5 – Summary questions

17. In summary, my assessment is that, after considering the suggestions I have made above*:

a) This small or sick newborn core quality indicator set is balanced across the whole newborn period 0-28 days.

Strongly disagree/ disagree/ neutral / agree / strongly agree

Optional Comments

18. In summary, my assessment is that, after considering the suggestions I have made above*:

b) This small or sick newborn core quality indicator set is balanced for both newborns surviving and thriving.

Strongly disagree/ disagree/ neutral / agree / strongly agree

Optional Comments

Online Survey Section 4: More Information

21. Please add any indicators for quality of care for small and sick newborns that are not in the lists above but that you would like to be considered as core indicators

22. Do you know of any countries contexts that currently regularly monitor indicators of quality of care for small or sick newborns and use the data to improve care for small or sick newborns? If yes, please complete table

4.2.5. Online Survey Section 5: Final Page

	Name of indicator	Country	Data source (e.g. newborn unit register, patient case notes, client interview, provider interview etc)	How is this indicator used - for what purpose?	Who uses this indicator?
Indicator 1	T	T	T	T	T
Indicator 2	T	T	T	T	T
Indicator 3	T	T	T	T	T
Indicator 4	T	T	T	T	T
Indicator 5	T	T	T	T	T
Indicator 6	T	T	T	T	T
Indicator 7	T	T	T	T	T
Indicator 8	T	T	T	T	T
Indicator 9	T	T	T	T	T
Indicator 10	T	T	T	T	T

Thank you for taking the time to provide your feedback. All comments received will be synthesized and inform the development of the final small or sick newborn quality indicators finalized by MoNITOR.

For any questions on how to complete the online survey, you can contact louise-tina.day@lshtm.ac.uk.

Online Survey - Develop Core Indicator set for quality of care for Small or Sick Newborns Online Global Consultation 2022 - References for Section 3.1 and 3.2

Section 3.1 - Proposed SSNB CORE quality Indicators - already prioritized for SSNB in existing global CORE indicator lists (SSNB as numerator/ denominator) (n = 9) – Metadata in Annex 4.1

Standard	Indicator Name	Classification (Paed QoC, otherwise MONITOR)	CORE list summary and definition
1	Pre-discharge neonatal mortality rate	Impact	QoC MNH[32], Health Facility Indicator[26], QoC Child[34]
1	Neonatal cause of death in health facilities	Impact	MoNITOR[31], QoC MNH[32], Health Facility Indicator[26],
1	Institutional low birth weight rate	Impact	MoNITOR[31], RMNCAH[27]
1	Institutional preterm birth rate	Impact	MoNITOR[31], (optional), RMNCAH[27] (additional)
1	Small for gestational age prevalence	Impact	MoNITOR[31],
1	Neonatal resuscitation - Bag and Mask Ventilation coverage (currently being redefined)	Process/ Output	MoNITOR[31], ENAP[1],
1	Antenatal corticosteroid coverage (currently being redefined)	Process/ Output	ENAP[1]
1	Neonatal sepsis/ infection treatment (currently being redefined)	Process/ Output	MoNITOR[31], ENAP[1]
1	Kangaroo Mother Care coverage (currently being redefined)	Process/ Output	MoNITOR[31], ENAP[1], QoC MNH[32], QoC Child[34], COVID-19[30]

Section 3.2 - Proposed SSNB CORE quality indicators - based on adaptation of existing prioritized global maternal, newborn and paediatric CORE health indicator recommendations (n=13) – Metadata in Annex 4.2

Standard	Indicator Name	Classification (Paed QoC, otherwise MONITOR)	Core list summary and definition
1	Danger signs, when to seek care, illness feeding	Process / Output	QoC Child[34]
2	Completion of medical record documentation patient key information at admission assessment, discharge and follow-up plans	Input	QoC Child[34]
2	Periodic review of SSNB quality of care data for the purpose of improving care	Process / Output	QoC Child[34]
4	Parent knowledge and understanding of newborn's condition/ treatment plan	Process / Output	QoC Child[34]
5	Child rights awareness	Process / Output	QoC Child[34]
5	Disrespectful care for the newborn or carer	Outcome (patient reported)	QoC Child[34]
6	Satisfaction with decision-making process for care	Outcome (patient-reported)	QoC Child[34]
6	Satisfaction with accompaniment during care/ rooming in	Process / Output	QoC Child[34]
7	Health worker density and distribution	Input	Health Facility Indicator[26]
7	Clinical mentorship or training	Input	QoC Child[34]
8	Basic sanitation amenities for carers and their families	Output	MoNITOR[31], QoC MNH[32],
8	Basic amenities for hand hygiene at facility unit entrance	Output	MoNITOR[31], QoC MNH[32],
8	No stock out three essential newborn medicines (1. first-line injectable antibiotics, 2. phenobarbitone 3. caffeine (or other methylxanthines))	Input	QoC Child[34], Health Facility Indicator[26]

Supplemental Material 9: Online consultation – respondent characteristics

Supplementary Table 2: SSNB Core Indicator Online Survey respondent background characteristics

		Overall,<br><br>N = 172	For organization, N=49	As individual, N=123
Language survey				
	English	142 (83%)		
	French	10 (6%)		
	Spanish	20 (12%)		
Stakeholder categories (multiple responses permitted)				
	WHO and UNICEF regional and country offices	17 (9.9%)	7 (14%)	10 (8.1%)
	Parent or parents' organization	8 (4.7%)	5 (10%)	3 (2.4%)
	Ministries of Health	20 (12%)	11 (22%)	9 (7.3%)
	Health worker	69 (40%)	15 (31%)	54 (44%)
	Professional organization	30 (17%)	15 (31%)	15 (12%)
	Implementing partner organization	46 (27%)	14 (29%)	32 (26%)
	Technical working group	17 (9.9%)	6 (12%)	11 (8.9%)
	Research institution	35 (20%)	7 (14%)	28 (23%)
	Independent expert	32 (19%)	3 (6.1%)	29 (24%)
	Work in the Public sector	40 (23%)	6 (12%)	34 (28%)
	Work in the Private sector	22 (13%)	6 (12%)	16 (13%)
Geographic scope of your organization's work (multiple responses permitted)				
	Global	75 (44%)	19 (39%)	56 (46%)
	Regional	58 (34%)	13 (27%)	45 (37%)
	National	83 (48%)	32 (65%)	51 (41%)
	Sub-national	39 (23%)	12 (24%)	27 (22%)
SDG regions in which organization performs its work (multiple responses permitted)				
	Sub-Saharan Africa	100 (58%)	18 (37%)	82 (67%)
	Northern Africa and Western Asia	33 (19%)	3 (6.1%)	30 (24%)
	Central and Southern Asia	37 (22%)	5 (10%)	32 (26%)
	Eastern and South-Eastern Asia	52 (30%)	19 (39%)	33 (27%)
	Latin America and the Caribbean	45 (26%)	7 (14%)	38 (31%)
	Oceania	14 (8.1%)	2 (4.1%)	12 (9.8%)
	Europe and Northern America	31 (18%)	9 (18%)	22 (18%)
Respondent cadres (multiple responses permitted)				
	Parent	7 (4.1%)	3 (6.1%)	4 (3.3%)
	Midwife	12 (7.0%)	5 (10%)	7 (5.7%)
	Nurse	24 (14%)	6 (12%)	18 (15%)
	Medical Doctor	95 (55%)	26 (53%)	69 (56%)
	Other medical professional	10 (5.8%)	5 (10%)	5 (4.1%)
	Public Health	58 (34%)	17 (35%)	41 (33%)
	Epidemiology	14 (8.1%)	2 (4.1%)	12 (9.8%)
	Research	46 (27%)	10 (20%)	36 (29%)
	Health management information specialist	8 (4.7%)	4 (8.2%)	4 (3.3%)
	Other (please specify below)	19 (11%)	10 (20%)	9 (7.3%)

Supplemental Material 10: Online consultation – frequency of selected country

Supplementary Table 3: Number of responses by 71 selected countries for indicator questions, respondent n=172

Countries	Respondents
Ethiopia	13
India	12
Nigeria	10
South Africa	8
Tanzania	8
Uganda	7
Argentina	6
Ghana	6
Malawi	6
Nepal	6
Kenya	5
Central African Republic	4
Afghanistan	3
Bangladesh	3
Czechia (Czech Republic)	3
Russia	3
United States of America	3
Cambodia	2
Cameroon	2
Chile	2
Colombia	2
Ecuador	2
Guatemala	2
Philippines	2
Rwanda	2
Senegal	2
Spain	2
Venezuela	2
Zambia	2
Albania	1
Australia	1
Bahrain	1
Benin	1
Bhutan	1
Brazil	1
Burkina Faso	1
Burundi	1
Canada	1
Chad	1
Congo (Congo-Brazzaville)	1
Costa Rica	1
Democratic Republic of the Congo	1
Djibouti	1
Honduras	1
Iraq	1
Italy	1

Jamaica	1
Jordan	1
Laos	1
Latvia	1
Lesotho	1
Liberia	1
Madagascar	1
Moldova	1
Mongolia	1
North Macedonia	1
Pakistan	1
Palestine State	1
Papua New Guinea	1
Peru	1
Sierra Leone	1
Slovenia	1
Somalia	1
South Sudan	1
Tajikistan	1
Timor-Leste	1
United Arab Emirates	1
United Kingdom	1
Uruguay	1
Yemen	1
Zimbabwe	1

Supplemental Material 11: Online consultation – results

SSNB Indicator perceived usefulness

Question: My assessment is that for monitoring and improving quality of SSNB care in the country specified above, this indicator will be: Very useful / Somewhat useful / Not very useful / Don't know Responses were ranked in order of frequency of "very useful" then "Somewhat useful" then "Not very useful" then "don't know". Results are shown in Supplementary figure 5

Supplementary figure 5: Potential 52 SSNB core quality indicators ranked by usefullness, online survey consultation, N =172

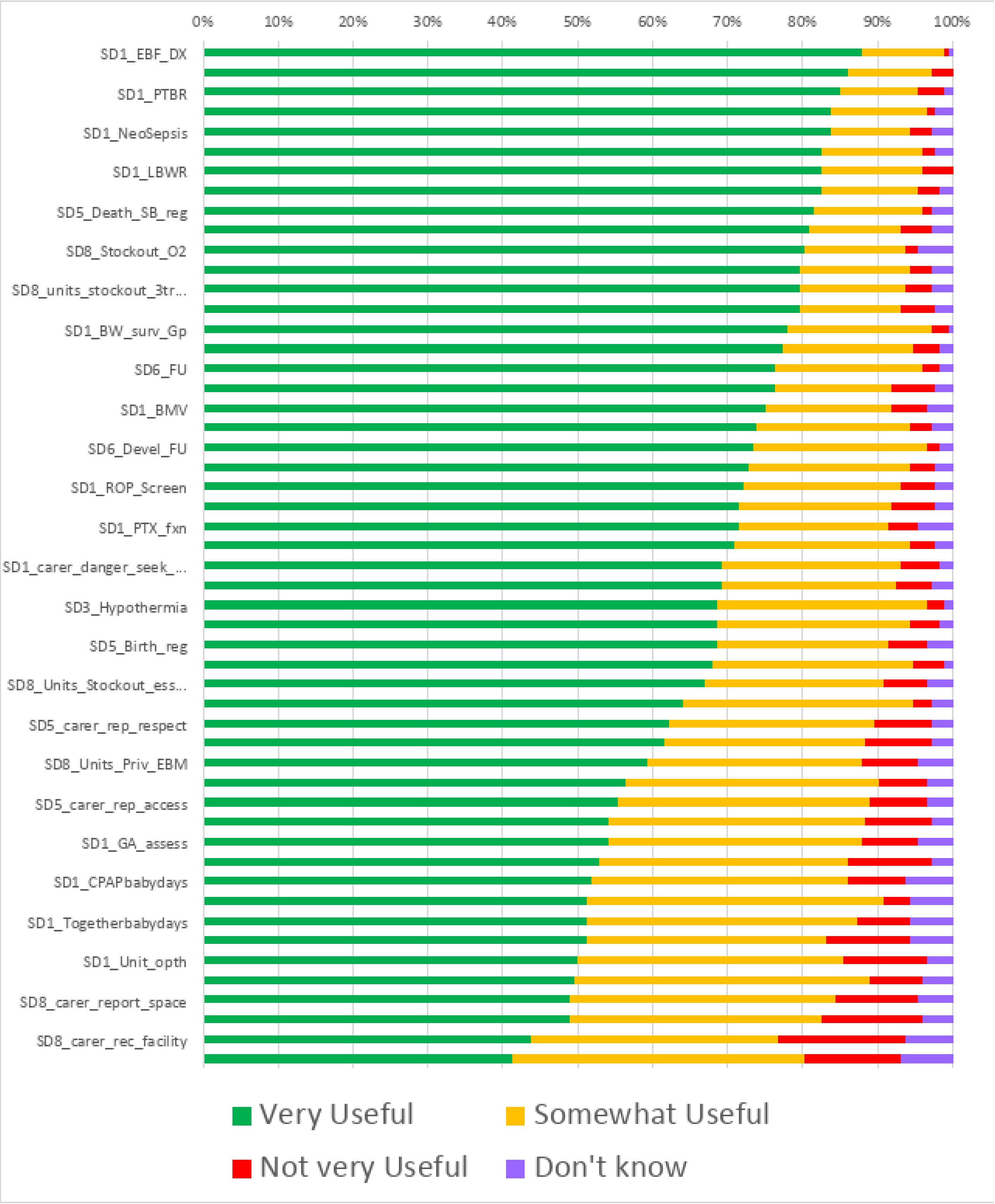

Core/Optional SSNB quality indicator selection

Question: My assessment for this proposed indicator is to be: Core / Optional / Neither (Supplementary figure 6) shows responses were ranked in order of frequency of "core" then "optional" then "neither".

Supplementary figure 6: Potential 52 Core Quality indicators ranked by selection as core/ optional/ neither, online survey consultation, N=172

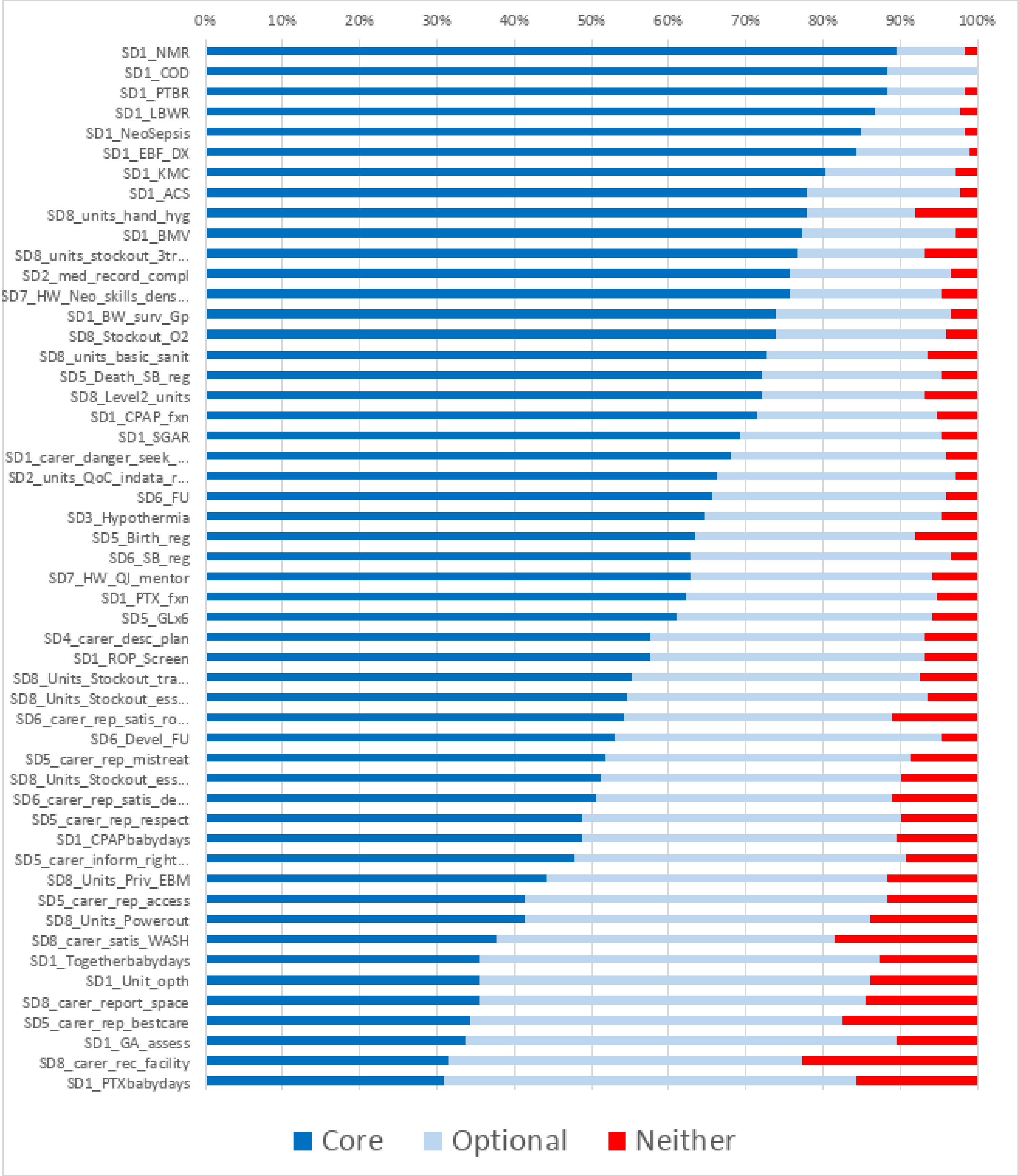

Supplementary figure 7 shows responses were ranked in order of frequency of "neither" then "optional" then "core".

Supplementary figure 7: Potential 52 SSNB Core Quality indicators ranked by selection as neither/ optional/ core, online survey consultation N=172

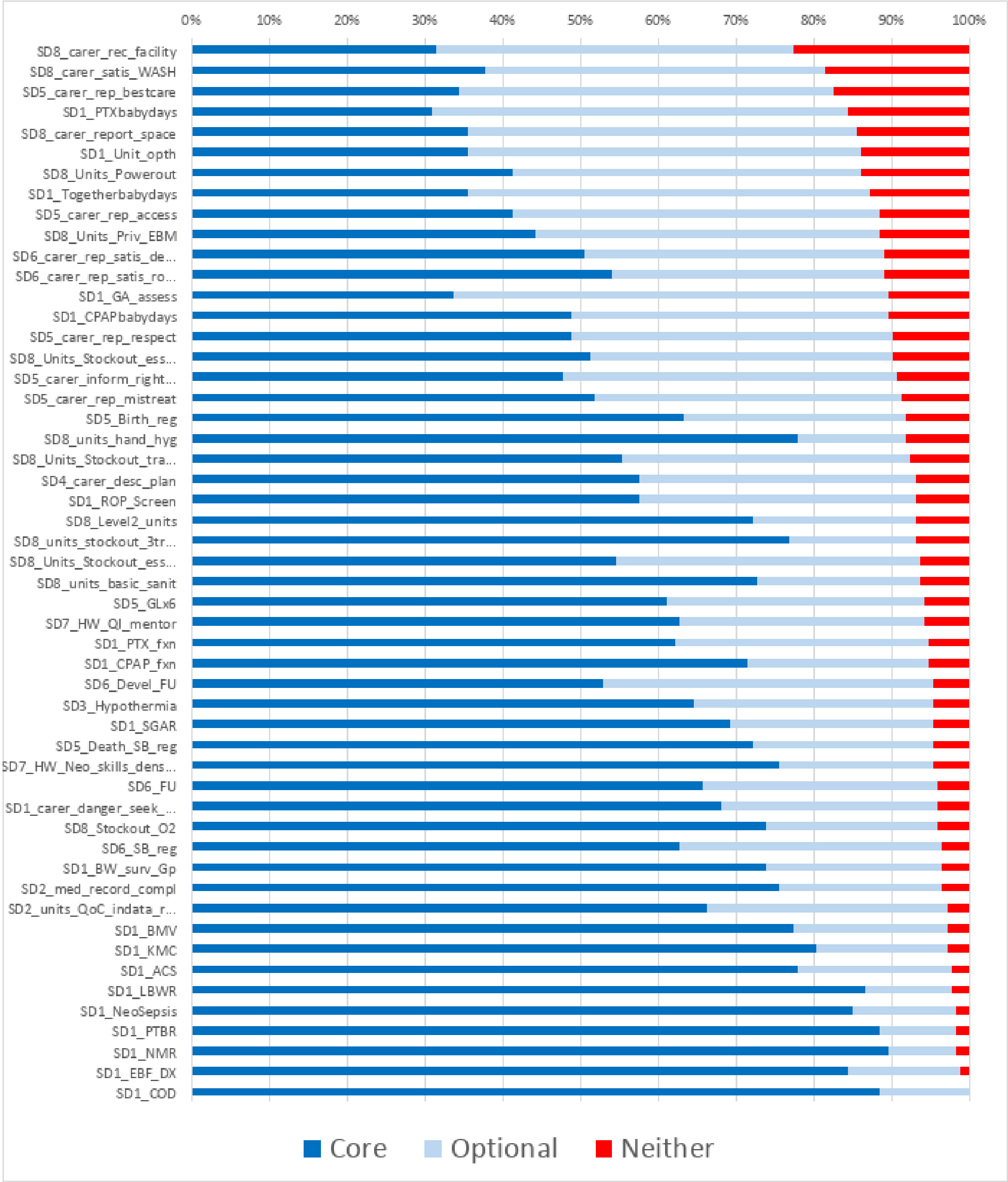

SSNB Indicator perceived feasibility to measure

Question: My estimation is that measurement of this indicator in the country specified above would be possible within a timeframe of: 2 years / 3-5 years / >5 years / Don't know
Supplementary figure 8 shows responses ranked by "2 years" to longer

Supplementary figure 8: Potential 52 SSN Core Quality indicators ranked by estimation of number of years to possible measurement "2 years" to longer, online survey consultation, N=172

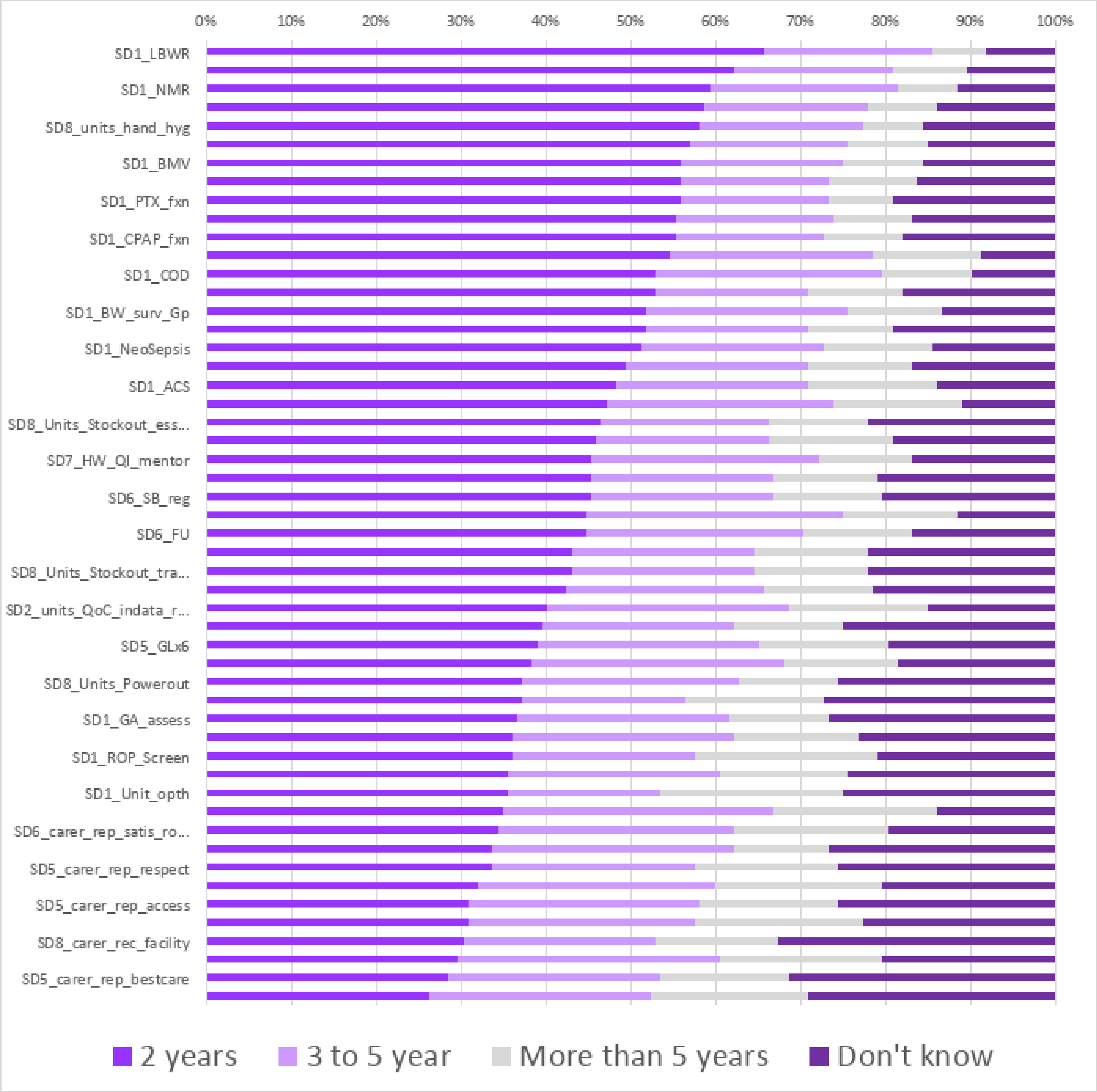

Supplementary figure 9 shows responses ranked by “don’t know” then “>5 years

Supplementary figure 9: SSN Core Quality indicators ranked by estimation of number of years to possible measurement ranking from "don't know" to longer, online survey consultation, N=172

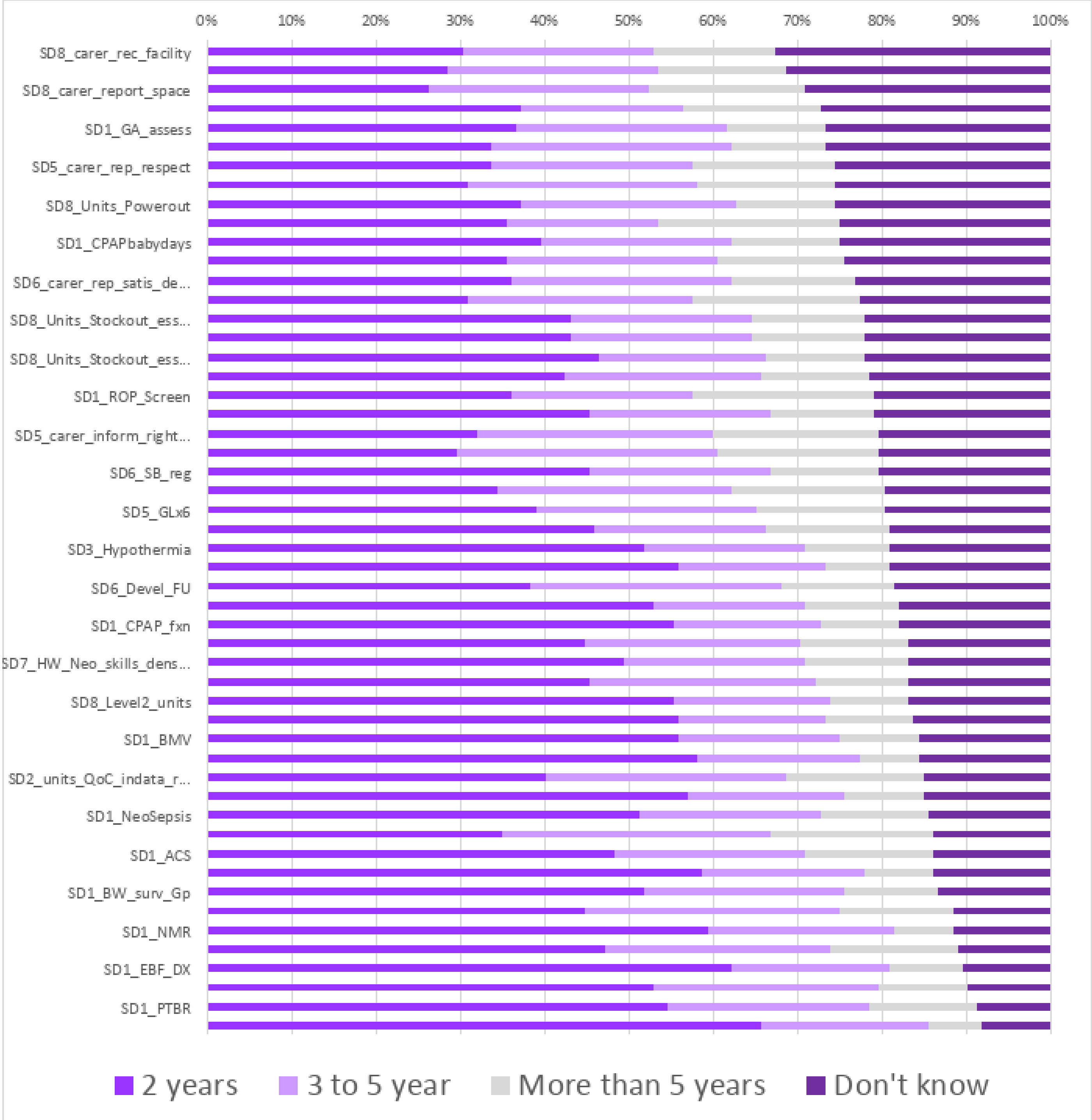

Supplemental Material 12: Combined Ranking Useful/ Core/ Measurable

Ranked 4 groups shown together

- SSN already specific CORE
- SSN if adapted CORE
- SSN QI proposed Standards CORE
- SSN QI proposed Standards OPTIONAL

Useful

Rank useful	Group	name	Very Useful
1	3.3	SD1_EBF_DX	151
2	3.1	SD1_COD	148
3	3.1	SD1_PTBR	146
4	3.1	SD1_NMR	144
5	3.1	SD1_NeoSepsis	144
6	3.1	SD1_KMC	142
7	3.1	SD1_LBWR	142
8	3.2	SD2_med_record_compl	142
9	3.3	SD5_Death_SB_reg	140
10	3.2	SD8_units_hand_hyg	139
11	3.3	SD8_Stockout_O2	138
12	3.4	SD1_CPAP_hm	137
13	3.2	SD8_units_stockout_3tracer_med	137
14	3.2	SD7_HW_Neo_skills_density	137
15	3.3	SD1_BW_surv_Gp	134
16	3.2	SD8_units_basic_sanit	133
17	3.4	SD6_FU	131
18	3.3	SD8_Level2_units	131
19	3.1	SD1_BMV	129
20	3.4	SD6_SB_reg	127
21	3.4	SD6_Devel_FU	126
22	3.1	SD1_ACS	125
23	3.4	SD1_ROP_Screen	124
24	3.4	SD8_Units_Stockout_essent_med	123
25	3.4	SD1_PTX_hm	123
26	3.4	SD5_GLx6	122
27	3.2	SD1_carer_danger_seek_feed	119
28	3.4	SD8_Units_Stockout_tracer3_device	119
29	3.3	SD3_Hypothermia	118
30	3.2	SD2_units_QoC_indata_rev	118
31	3.3	SD5_Birth_reg	118
32	3.2	SD7_HW_QI_mentor	117
33	3.4	SD8_Units_Stockout_essent_device	116
34	3.1	SD1_SGAR	110
35	3.4	SD5_carer_rep_respect	107
36	3.2	SD6_carer_rep_satis_room_in	106
37	3.4	SD8_Units_Priv_EBM	102
38	3.2	SD4_carer_desc_plan	97
39	3.4	SD5_carer_rep_access	95
40	3.2	SD6_carer_rep_satis_decision	93
41	3.4	SD1_GA_assess	93
42	3.4	SD8_Units_Powerout	91
43	3.4	SD1_CPAPbabydays	89
44	3.2	SD5_carer_rep_mistreat	88
45	3.4	SD1_Togetherbabydays	88
46	3.4	SD8_carer_satis_WASH	88
47	3.4	SD1_Unit_opth	86
48	3.2	SD5_carer_inform_rights	85
49	3.4	SD8_carer_report_space	84
50	3.4	SD5_carer_rep_bestcare	84

Core

Rank Core	Group	name	Core
1	3.1	SD1_NMR	154
2	3.1	SD1_COD	152
3	3.1	SD1_PTBR	152
4	3.1	SD1_LBWR	149
5	3.1	SD1_NeoSepsis	146
6	3.3	SD1_EBF_DX	145
7	3.1	SD1_KMC	138
8	3.1	SD1_ACS	134
9	3.2	SD8_units_hand_hyg	134
10	3.1	SD1_BMV	133
11	3.2	SD8_units_stockout_3tracer_med	132
12	3.2	SD2_med_record_compl	130
13	3.2	SD7_HW_Neo_skills_density	130
14	3.3	SD1_BW_surv_Gp	127
15	3.3	SD8_Stockout_O2	127
16	3.2	SD8_units_basic_sanit	125
17	3.3	SD5_Death_SB_reg	124
18	3.3	SD8_Level2_units	124
19	3.4	SD1_CPAP_hm	123
20	3.1	SD1_SGAR	119
21	3.2	SD1_carer_danger_seek_feed	117
22	3.2	SD2_units_QoC_indata_rev	114
23	3.4	SD6_FU	113
24	3.3	SD3_Hypothermia	111
25	3.3	SD5_Birth_reg	109
26	3.4	SD6_SB_reg	108
27	3.2	SD7_HW_QI_mentor	108
28	3.4	SD1_PTX_hm	107
29	3.4	SD5_GLx6	105
30	3.4	SD1_ROP_Screen	99
31	3.2	SD4_carer_desc_plan	99
32	3.4	SD8_Units_Stockout_tracer3_device	95
33	3.4	SD8_Units_Stockout_essent_med	94
34	3.2	SD6_carer_rep_satis_room_in	93
35	3.4	SD6_Devel_FU	91
36	3.2	SD5_carer_rep_mistreat	89
37	3.4	SD8_Units_Stockout_essent_device	88
38	3.2	SD6_carer_rep_satis_decision	87
39	3.4	SD5_carer_rep_respect	84
40	3.4	SD1_CPAPbabydays	84
41	3.2	SD5_carer_inform_rights	82
42	3.4	SD8_Units_Priv_EBM	78
43	3.4	SD5_carer_rep_access	71
44	3.4	SD8_Units_Powerout	71
45	3.4	SD8_carer_satis_WASH	65
46	3.4	SD1_Togetherbabydays	61
47	3.4	SD1_Unit_opth	61
48	3.4	SD8_carer_report_space	61
49	3.4	SD5_carer_rep_bestcare	59
50	3.4	SD1_GA_assess	58

Measure

Rank meas.	Group	name	2 years
1	3.1	SD1_LBWR	113
2	3.3	SD1_EBF_DX	107
3	3.1	SD1_NMR	102
4	3.1	SD1_KMC	101
5	3.2	SD8_units_hand_hyg	100
6	3.2	SD8_units_basic_sanit	98
7	3.1	SD1_BMV	96
8	3.3	SD8_Stockout_O2	96
9	3.4	SD1_PTX_hm	96
10	3.3	SD8_Level2_units	95
11	3.4	SD1_CPAP_hm	95
12	3.1	SD1_PTBR	94
13	3.1	SD1_COD	91
14	3.2	SD8_units_stockout_3tracer_med	91
15	3.3	SD1_BW_surv_Gp	89
16	3.3	SD3_Hypothermia	89
17	3.1	SD1_NeoSepsis	88
18	3.2	SD7_HW_Neo_skills_density	85
19	3.1	SD1_ACS	83
20	3.1	SD1_SGAR	81
21	3.4	SD8_Units_Stockout_essent_med	80
22	3.3	SD5_Death_SB_reg	79
23	3.2	SD7_HW_QI_mentor	78
24	3.4	SD6_SB_reg	78
25	3.3	SD5_Birth_reg	78
26	3.2	SD2_med_record_compl	77
27	3.4	SD6_FU	77
28	3.4	SD8_Units_Stockout_tracer3_device	74
29	3.4	SD8_Units_Stockout_essent_device	74
30	3.4	SD8_Units_Priv_EBM	73
31	3.2	SD2_units_QoC_indata_rev	69
32	3.4	SD1_CPAPbabydays	68
33	3.4	SD5_GLx6	67
34	3.4	SD6_Devel_FU	66
35	3.4	SD8_Units_Powerout	64
36	3.4	SD8_carer_satis_WASH	64
37	3.4	SD1_GA_assess	63
38	3.2	SD6_carer_rep_satis_decision	62
39	3.4	SD1_ROP_Screen	62
40	3.4	SD1_PTXbabydays	61
41	3.4	SD1_Unit_opth	61
42	3.2	SD1_carer_danger_seek_feed	60
43	3.2	SD6_carer_rep_satis_room_in	59
44	3.4	SD1_Togetherbabydays	58
45	3.4	SD5_carer_rep_respect	58
46	3.2	SD5_carer_inform_rights	55
47	3.4	SD5_carer_rep_access	53
48	3.2	SD5_carer_rep_mistreat	53
49	3.4	SD5_carer_rec_facility	52
50	3.2	SD4_carer_desc_plan	51

Ranked by group shown online survey

- SSN already specific CORE
- SSN if adapted CORE
- SSN QI proposed Standards CORE
- SSN QI proposed Standards OPTIONAL

ext

Useful

Rank useful	Rank Core	Rank measure	Group	name
1	6	2	3.3	SD1_EBF_DX
9	17	22	3.3	SD5_Death_SB_reg
11	15	8	3.3	SD8_Stockout_O2
15	14	15	3.3	SD1_BW_surv_Gp
18	18	10	3.3	SD8_Level2_units
29	24	16	3.3	SD3_Hypothermia
31	25	25	3.3	SD5_Birth_reg
8	12	26	3.2	SD2_med_record_compl
10	9	5	3.2	SD8_units_hand_hyg
13	11	14	3.2	SD8_units_stockout_3tracer_med
14	13	18	3.2	SD7_HW_Neo_skills_density
16	16	6	3.2	SD8_units_basic_sanit
27	21	42	3.2	SD1_carer_danger_seek_feed
30	22	31	3.2	SD2_units_QoC_indata_rev
32	27	23	3.2	SD7_HW_QI_mentor
36	34	43	3.2	SD6_carer_rep_satis_room_in
38	31	50	3.2	SD4_carer_desc_plan
40	38	38	3.2	SD6_carer_rep_satis_decision
44	36	48	3.2	SD5_carer_rep_mistreat
48	41	46	3.2	SD5_carer_inform_rights
2	2	13	3.1	SD1_COD
3	3	12	3.1	SD1_PTBR
4	1	3	3.1	SD1_NMR
5	5	17	3.1	SD1_NeoSepsis
6	7	4	3.1	SD1_KMC
7	4	1	3.1	SD1_LBWR
19	10	7	3.1	SD1_BMV
22	8	19	3.1	SD1_ACS
34	20	20	3.1	SD1_SGAR
12	19	11	3.4	SD1_CPAP_hm
17	23	27	3.4	SD6_FU
20	26	24	3.4	SD6_SB_reg
21	35	34	3.4	SD6_Devel_FU
23	30	39	3.4	SD1_ROP_Screen
24	33	21	3.4	SD8_Units_Stockout_essent_med
25	28	9	3.4	SD1_PTX_hm
26	29	33	3.4	SD5_GLx6
28	32	28	3.4	SD8_Units_Stockout_tracer3_device
33	37	29	3.4	SD8_Units_Stockout_essent_device
35	39	45	3.4	SD5_carer_rep_respect
37	42	30	3.4	SD8_Units_Priv_EBM
39	43	47	3.4	SD5_carer_rep_access
41	50	37	3.4	SD1_GA_assess
42	44	35	3.4	SD8_Units_Powerout
43	40	32	3.4	SD1_CPAPbabydays
45	46	44	3.4	SD1_Togetherbabydays
46	45	36	3.4	SD8_carer_satis_WASH
47	47	41	3.4	SD1_Unit_opth
49	48	52	3.4	SD8_carer_report_space
50	49	51	3.4	SD5_carer_rep_bestcare

Core

Rank useful	Rank Core	Rank measure	Group	name
1	6	2	3.3	SD1_EBF_DX
15	14	15	3.3	SD1_BW_surv_Gp
11	15	8	3.3	SD8_Stockout_O2
9	17	22	3.3	SD5_Death_SB_reg
18	18	10	3.3	SD8_Level2_units
29	24	16	3.3	SD3_Hypothermia
31	25	25	3.3	SD5_Birth_reg
10	9	5	3.2	SD8_units_hand_hyg
13	11	14	3.2	SD8_units_stockout_3tracer_med
8	12	26	3.2	SD2_med_record_compl
14	13	18	3.2	SD7_HW_Neo_skills_density
16	16	6	3.2	SD8_units_basic_sanit
27	21	42	3.2	SD1_carer_danger_seek_feed
30	22	31	3.2	SD2_units_QoC_indata_rev
32	27	23	3.2	SD7_HW_QI_mentor
38	31	50	3.2	SD4_carer_desc_plan
36	34	43	3.2	SD6_carer_rep_satis_room_in
44	36	48	3.2	SD5_carer_rep_mistreat
40	38	38	3.2	SD6_carer_rep_satis_decision
48	41	46	3.2	SD5_carer_inform_rights
4	1	3	3.1	SD1_NMR
2	2	13	3.1	SD1_COD
3	3	12	3.1	SD1_PTBR
7	4	1	3.1	SD1_LBWR
5	5	17	3.1	SD1_NeoSepsis
6	7	4	3.1	SD1_KMC
22	8	19	3.1	SD1_ACS
19	10	7	3.1	SD1_BMV
34	20	20	3.1	SD1_SGAR
12	19	11	3.4	SD1_CPAP_hm
17	23	27	3.4	SD6_FU
20	26	24	3.4	SD6_SB_reg
25	28	9	3.4	SD1_PTX_hm
26	29	33	3.4	SD5_GLx6
23	30	39	3.4	SD1_ROP_Screen
28	32	28	3.4	SD8_Units_Stockout_tracer3_device
24	33	21	3.4	SD8_Units_Stockout_essent_med
21	35	34	3.4	SD6_Devel_FU
33	37	29	3.4	SD8_Units_Stockout_essent_device
35	39	45	3.4	SD5_carer_rep_respect
43	40	32	3.4	SD1_CPAPbabydays
37	42	30	3.4	SD8_Units_Priv_EBM
39	43	47	3.4	SD5_carer_rep_access
42	44	35	3.4	SD8_Units_Powerout
46	45	36	3.4	SD8_carer_satis_WASH
45	46	44	3.4	SD1_Togetherbabydays
47	47	41	3.4	SD1_Unit_opth
49	48	52	3.4	SD8_carer_report_space
50	49	51	3.4	SD5_carer_rep_bestcare
41	50	37	3.4	SD1_GA_assess

Supplemental Material 13: Prioritized indicators to measure quality of care for small and/or sick newborns (SSNB), metadata, online survey ranking and rationale for inclusion

Linked to manuscript Table 1

Supplementary Table 3: Prioritized indicators to measure quality-of-care for small and/or sick newborns (SSNB) categorized by WHO quality domain standards 1-8, indicator type (input, process/ output, outcome), context of care and online survey ranking
A) All SSNB cared for in all health facility wards (labour & delivery ward, postnatal ward, KMC ward, neonatal wards/units) (n=10 indicators)
B) Subset of SSNB admitted to neonatal wards/units only: defined (n=17 indicators), needing definitional work (n=3 indicators).

Context/ Quality Standard / framework outcome	Indicator name	Indicator definition	Classification (Paed QoC, otherwise MoNITOR)	Service level (ward) for measurement	Numerator	Denominator	Proposed			Prioritized list alignment and definition *	Online survey ranking			Rationale
							Disaggregation	Data source	Report frequency		Useful rank	Core rank	Measure 2y rank	
A) All SSNB cared for in all health facility wards (labour & delivery ward, postnatal ward, KMC ward, neonatal wards/units) (n=10 indicators)														
Context for inpatient SSNB care	Inpatient admissions among newborns	# or % of inpatient admissions among newborns	Output/process<br>*(Donabedian = process)*	All health facility wards	# of inpatient neonatal (0-27 days) admissions	(if calculating %) Estimated total population of neonates	inborn/ outborn, sex, type of neonate ward (e.g., level 1,2,3), postnatal ward, paediatric ward, labour and delivery ward, emergency room, reason for admission	RHIS patient data: patient charts/ case notes, registers	Monthly	MoNITOR[6], MNCAH[3], COVID 19 Routine Data[11]	Not in survey			Adopted to measure quality (Donabedian process) This indicator measures case mix and service demand in a population. The indicator data has potential for use for a set of actions at health systems, health facility, physical and human resources, clinical and educational planning for actions including intrapartum care.<br>Survive and Thrive: day 1-27

Context/ Quality Standard / framework outcome	Indicator name	Indicator definition	Classification (Paed QoC, otherwise MoNITOR)	Service level (ward) for measurement	Numerator	Denominator	Proposed			Prioritized list alignment and definition *	Online survey ranking			Rationale
							Disaggregation	Data source	Report frequency		Useful rank	Core rank	Measure 2y rank	
1	Neonatal resuscitation	% of newborns who received positive-pressure ventilation at birth	Process/ Output<br>*(Donabedian = process)*	All wards: focus Labour & Delivery	# of neonates receiving positive-pressure ventilation at birth	# of neonates born in the health facility not breathing well at birth.~	health facility type	RHIS patient data: patient charts/ case notes, registers	Monthly	ENAP coverage[1,2], MNCAH[3]	19	9	9	Adopted to measure quality (Donabedian = process) of an intervention directly addresses a major cause of neonatal death/disability (asphyxia), indirectly into the quality of intrapartum care and severity of intrapartum-related events,<br>This indicator will give insight into coverage of provision of care around the time of birth including preterm infants unprepared with ACS. Cross-cutting quality domains include highlighting available equipment.<br>Survive and Thrive: day 1
1	Kangaroo Mother Care (KMC)† initiated	% of admitted neonates weighing <2500g initiated on Kangaroo Mother Care anywhere in the health facility	Process/ Output<br>*(Donabedian = process)*	All wards: Labour & Delivery, postnatal, KMC neonatal unit	# of admitted neonates with a birthweight <2500g who were initiated on KMC (placed in the kangaroo position) anywhere in the health facility	# of admitted with a birthweight <2500g anywhere in the facility	1000-1499g, 1500-1999g, 2000-2499g	RHIS patient data: patient charts/ case notes, registers	Monthly	ENAP coverage[1,2], MNCAH[3]	6	7	4	Adopted to measure quality (Donabedian = process) of an intervention which directly addresses a major cause of neonatal death/ disability - prematurity/ Low birthweight.<br>This indicator will give insight at patient level into coverage of an intervention that has been slow to scale up for decades.<br>Cross-cutting quality domains include highlighting needed changes in infrastructure and staffing<br>Survive and Thrive: day 1-27
1	Antibiotic treatment for neonatal infection	% of neonates (0-28 days) identified as clinically suspected sepsis (serious bacterial infection)‡ in inpatient settings, initiated who received at least two days of appropriate injectable antibiotics	Process/ Output<br>*(Donabedian = process)*	All wards: Labour & Delivery, postnatal, KMC, neonatal unit	# of neonates identified as cases of clinically suspected sepsis‡ in inpatient settings, who received at least two days of appropriate injectable antibiotics	# of neonates identified as having clinically suspected sepsis‡ in inpatient settings	Inborn/ outborn	RHIS patient data: patient charts/ case notes, registers	Monthly	ENAP coverage[1,2] MNCAH[3]	5	5	17	Adopted to measure quality (Donabedian process) of an intervention that directly addresses a major cause of neonatal death/disability – sepsis.<br>This indicator will give insight at patient level into appropriate use (used when needed, not used when not needed). Cross-cutting quality domains include highlighting stockouts, and potentially overuse.<br>Survive and Thrive: day 1-27

Context/ Quality Standard / framework outcome	Indicator name	Indicator definition	Classification (Paed QoC, otherwise MoNITOR)	Service level (ward) for measurement	Numerator	Denominator	Proposed			Prioritized list alignment and definition *	Online survey ranking			Rationale
							Disaggregation	Data source	Report frequency		Useful rank	Core rank	Measure 2y rank	
1	Antenatal corticosteroids	% of women who delivered between 24- and 34- weeks gestational age who received at least one dose of antenatal corticosteroids	Process/ Output<br>*(Donabedian = process)*	All wards: Labour & Delivery, postnatal, neonatal unit	# of women who delivered between 24- and 34- weeks gestational age who received at least one dose of antenatal corticosteroids	# of women who delivered between 24- and 34- weeks gestational age	Inborn/ outborn	RHIS patient data: patient charts/ case notes, registers	Monthly	ENAP coverage[1,2] MNCAH[3]	22	8	19	Adopted to measures quality (Donabedian = process) of an intervention that directly addresses a major cause of neonatal death/ disability – prematurity.<br>This indicator will give insight into an intervention requiring collaboration with maternal care providers for secondary prevention of neonatal morbidity. Cross-cutting quality domains include highlighting stockouts.,<br>Survive and Thrive: day 1-27
**Facility /individual level outcome**	In-facility§ late gestation‡‡‡ stillbirth	% of in-facility§ stillbirths⁺⁺ among late gestation‡‡‡ total births	Impact<br>*(Donabedian = outcome)*	All wards: focus Labour & Delivery	# of in-facility⁺ late gestation‡‡‡ stillbirths	# of late gestation‡‡‡ total births x1000	1000-1499g, 1500-1999g, 2000-2499g, 2500 -3999g, ≥ 4000g<br><br>preterm (<37+0 weeks, <259 days), term (37+0 to 41+6 weeks, 259–293 days), post-term (≥42+0 weeks, ≥294 days)	RHIS patient data: patient charts/ case notes, registers	Monthly	UNICEF [4], ICD 11[5]	Not in survey			Adopted to measure quality (Donabedian = outcome) for a group of children that represent a substantial contribution to perinatal mortality, prioritized by ICD and ENAP, Global strategy. but not an SDG.<br>This Indicator will give insight into the quality of intrapartum care (labour monitoring and management) and severity of intrapartum-related events and needs to be considered in tandem with the newborn resuscitation indicator and early neonatal mortality rate due to frequent misclassification.<br>Survive and Thrive: day 0

Context/ Quality Standard / framework outcome	Indicator name	Indicator definition	Classification (Paed QoC, otherwise MoNITOR)	Service level (ward) for measurement	Numerator	Denominator	Proposed			Prioritized list alignment and definition *	Online survey ranking			Rationale
							Disaggregation	Data source	Report frequency		Useful rank	Core rank	Measure 2y rank	
Facility /individual level outcome	Low birthweight (<2500 g)	Proportion of live births in facilities with birthweight < 2500 g	Outcome<br>*(Donabedian = outcome)*	All wards: focus Labour & Delivery	# Number of newborns born alive in a facility with birthweight <2500 g at birth	# of live births in facility	1000-1499g, 1500-1999g, (and subgroup <2000g), 2000-2499g	RHIS patient data: patient charts/ case notes, registers	Monthly	MNCAH[3] MoNITOR[6],	7	4	1	Adopted to measure quality (Donabedian = outcome) for the target "small" newborns that directly addresses a major cause of neonatal death/ disability – low birth weight (prematurity and growth restricted infants). Birthweight measurement is needed to risk stratify for clinical care and follow up.<br>This indicator will give insight regarding case mix to enable service delivery planning as higher volume centres are typically associated with better outcomes. For a set of actions at health systems, health facility, physical and human resources, clinical and educational planning for actions including KMC, alternative feeding methods, and longer inpatient stay before meeting discharge criteria. Aligns with improving quality (Donabedian = process) of birthweight measurement (WHO SSNB Quality standard 1 Evidence-based practices) to identify "small newborns", including the target group for KMC. Also aligns with improving WHO SSNB Quality standard 2 "Actionable information systems by strengthening health facility reporting of the low birthweight global nutrition target as well as KMC indicator interpretation.<br>Survive and Thrive: day 1

Context/ Quality Standard / framework outcome	Indicator name	Indicator definition	Classification (Paed QoC, otherwise MoNITOR)	Service level (ward) for measurement	Numerator	Denominator	Proposed			Prioritized list alignment and definition *	Online survey ranking			Rationale
							Disaggregation	Data source	Report frequency		Useful rank	Core rank	Measure 2y rank	
**Facility /individual level outcome**	Preterm birth	% of births in facilities that are preterm (< 37 completed weeks gestation)	Outcome<br><br>*(Donabedian = outcome)*	All wards: focus Labour & Delivery	# of neonates born <37 weeks completed gestation	# of live births in facility	<28 weeks, 28-31 weeks, 32-36 weeks	RHIS patient data: patient charts/ case notes, registers	Monthly	MoNITOR[6], MNCAH[3]	3	3	12	Adopted to measure quality (Donabedian = outcome) for the target "small and/or sick" group requiring inpatient care that directly addresses a major cause of neonatal death/ disability – prematurity. Gestation is needed to risk stratify for clinical care and follow up.<br>This indicator will give insight regarding case mix to enable service delivery planning as higher volume centres are typically associated with better outcomes. For use for a set of actions at health systems, health facility, physical and human resources, clinical and educational planning for actions including KMC, alternative feeding methods, and longer inpatient stay before meeting discharge criteria. Aligns with improving quality of gestational assessment (Donabedian = process) to identify "small and/or sick" newborns (WHO SSNB Quality standard 1 Evidence-based practices). This indicator will gain reliability with increasing use of first trimester ultrasound dating.<br>Also aligns with improving WHO SSNB Quality standard 2 "Actionable information systems, by strengthening health facility reporting of the gestational age.<br>Survive and Thrive: day 1-3

Context/ Quality Standard / framework outcome	Indicator name	Indicator definition	Classification (Paed QoC, otherwise MoNITOR)	Service level (ward) for measurement	Numerator	Denominator	Proposed			Prioritized list alignment and definition *	Online survey ranking			Rationale
							Disaggregation	Data source	Report frequency		Useful rank	Core rank	Measure 2y rank	
**Facility /individual level outcome**	Small for gestational age	% of neonates with birthweight below the 10th percentile of the expected weight for gestational age of the reference population.	Outcome<br>*(Donabedian = outcome)*	All wards: focus Labour & Delivery	# of neonates with birthweight below the 10th percentile of the expected weight for gestational age of the reference population	# of live births		RHIS patient data: patient charts/ case notes, registers	Monthly	MoNITOR[6]	34	20	20	Adopted to measure quality (Donabedian = outcome) regarding how the health facility identifies a crucial clinical subset of "small and/or sick" group requiring inpatient care that directly addresses a major cause of neonatal death/ disability – that is very prevalent in many high mortality settings. Comparing birth weight and gestation enables risk stratification for clinical care and follow up.<br>This indicator will give insight regarding case mix to enable service delivery planning and set of actions at health systems, health facility, physical and human resources, clinical and educational planning for actions that are different to low birth weight and preterm including cross-cutting with obstetrics. This indicator will gain reliability with improving gestational assessment. Survive and Thrive: day 1-3
**Facility /individual level outcome**	Institutional neonatal mortality	% of inpatient neonatal deaths 0–27 days in health facilities	Impact<br>*(Donabedian = outcome)*	All wards: focus Labour & Delivery, postnatal, KMC newborn unit	Number of inpatient deaths	# of discharges (including deaths) among newborns age 0-27 days	inborn/ outborn, type of newborn ward (e.g., level 1,2,3), postnatal ward, paediatric ward, labour and delivery ward, emergency room	RHIS patient data: patient charts/ case notes, registers	Monthly	MNCAH[3], Health Facility Indicator[7], QoC MNH[8], QoC Child[9]	4	1	3	Adopted to measure quality (Donabedian = outcome) for newborns across the health facility, as not all SSNB will be correctly identified. Indicator will give insight regarding case mix alongside consideration of all other indicators in this set for benchmarking and needs to be considered in tandem with the in-facility stillbirth rate due to frequent misclassification. Survive and Thrive: day 1-27

Context/ Quality Standard / framework outcome	Indicator name	Indicator definition	Classification (Paed QoC, otherwise MoNITOR)	Service level (ward) for measurement	Numerator	Denominator	Proposed			Prioritized list alignment and definition *	Online survey ranking			Rationale
							Disaggregation	Data source	Report frequency		Useful rank	Core rank	Measure 2y rank	
Facility /individual level outcome	Neonatal cause of death in health facilities	% of neonatal deaths (days 0-27 of life) of a specified cause≠	Impact<br><br>*(Donabedian = outcome)*	All wards: Labour & Delivery, postnatal, KMC newborn unit	# of neonatal deaths (days 0-27 of life) of a specified cause≠	# of neonatal deaths	inborn/ outborn,<br><br>1000-1499g, 1500-1999g, 2000-2499g 2500 -3999g, ≥ 4000g	RHIS patient data: patient charts/ case notes, registers	Monthly	MoNITOR[6], Health Facility Indicator[7], QoC MNH[8]	2	2	13	Adopted to measure quality (Donabedian = outcome) for newborns across the health facility, as not all SSNB will be correctly identified. Indicator will give insight regarding case mix alongside consideration of all other indicators in this set for benchmarking and is essential to interpret neonatal mortality in facility and target quality improvement interventions.<br>Survive and Thrive: day 1-27

Standard	Indicator Name	Indicator definition	Classification (based Paed QoC, otherwise MoNITOR)	Health facility service Level (ward) for measurement	Numerator	Denominator	Proposed			Prioritized list alignment and definition	Online consultation survey ranking			Rationale
							Disaggregation	Data source	Report frequency		Useful rank	Core rank	Measure 2y rank	
B) Subset of SSNB admitted to neonatal wards/ units only: defined (n=17 indicators), needing definitional work (n=3 indicators).														
Context for inpatient SSNB care	Level 2 inpatient unit for small and/or sick newborns	% of districts/ sub-national areas with one or more level 2 neonatal units	Input<br><br>*(Donabedian = structure)*	Neonatal unit	# of districts/ sub-national areas with one or more level 2 neonatal units	# of districts/ sub-national areas assessed	health facility type (level 1, 2, 3)	RHIS facility data with periodic survey	Quarterly	ENAP coverage target[10]	17	18	10	Adopted to measure quality (Donabedian= structure) of health system performance and capacity for SSNB care.<br>Survive and Thrive: day 1-27

Standard	Indicator Name	Indicator definition	Classification (based Paed QoC, otherwise MoNITOR)	Health facility service Level (ward) for measurement	Numerator	Denominator	Proposed			Prioritized list alignment and definition	Online consultation survey ranking			Rationale
							Disaggregation	Data source	Report frequency		Useful rank	Core rank	Measure 2y rank	
1	Assessment of Gestational age	% of neonates less than 96 h of age who are admitted to a neonatal unit with unknown gestational age and are assessed with an appropriate gestational age scoring tool	Process/ Output (patient level)<br><br>*(Donabedian = process)*	Neonatal unit	# of neonates less than 96 h of age who are admitted to a neonatal unit with unknown gestational age and are assessed with an appropriate gestational age scoring tool before 24 h of age	# of neonates less than 96 h of age who are admitted to the neonate unit with unknown gestational age	health facility type,	RHIS patient data: patient charts/ case notes, registers	Monthly	New from WHO SSNB Quality Standards[12]	40 adapt	50 adapt	38 adapt	New prioritized quality indicator from WHO SSNB Quality standard 1 "Evidence-based practices", statement 1.11. (Donabedian process) for measuring every newborn admitted to a neonatal unit has a gestational age either from antenatal care or if not then postnatal. Prematurity is the leading cause of death and disability for newborns and under five age children. This indicator data has potential to highlight the importance of more accurate first trimester gestational age assessment in utero, which is currently a priority in high mortality settings. Postnatal gestational age assessment is a useful addition when obstetric gestational age assessment is uncertain, to determine clinical risk immediately and for follow up, determine care plans, and is a component of prioritized indicators: preterm birth rate, antenatal corticosteroids.<br>Survive and Thrive: day 1-3

Standard	Indicator Name	Indicator definition	Classification (based Paed QoC, otherwise MoNITOR)	Health facility service Level (ward) for measurement	Numerator	Denominator	Proposed			Prioritized list alignment and definition	Online consultation survey ranking			Rationale
							Disaggregation	Data source	Report frequency		Useful rank	Core rank	Measure 2y rank	
1	Screening and treatment of Retinopathy of Prematurity (ROP)	% of neonatal units providing regular** Retinopathy of Prematurity (ROP) screening†† and treatment‡‡	Input<br><br>*(Donabedian = structure)*	Neonatal unit	# of neonate units providing regular** Retinopathy of Prematurity (ROP) screening†† and treatment‡‡	# of health facilities with neonatal units assessed during the reporting period	Screening only, both screening and treatment, health facility type	RHIS facility data, including from network of specialist eye hospitals	Monthly	New from WHO SSNB Quality Standards[12], Vermont Oxford Network[13,14] general discussion with ROP experts LSHTM]	47	47	41	New prioritized quality indicator from WHO SSNB Quality standard 1 "Evidence-based practices", statement 1.3. to measure quality (Donabedian structure) for the leading cause of acquired blindness in children. This indicator will give insight into coverage of provision of care detecting preventable blindness, actionable by using safer oxygen systems. This is important balancing measure as level 2 care is scaled up, increasing newborns' exposure to oxygen (including CPAP). A facility level indicator is needed currently due to the current absence of any ophthalmological services in many high mortality settings. Cross-cutting quality domains include highlighting available medical records, equipment, human resources.<br>Survive and Thrive: day 1-27
2	Completion of standardized individual small and/or sick neonate case notes	% of neonatal case notes with complete key clinical information at admission§§ and discharge¶¶	Process. /Output/<br><br>(facility level)<br><br>*(Donabedian = process)*	Neonatal unit	# of neonatal case notes with complete key clinical information at admission§§ and discharge¶¶	# of neonatal case notes assessed during reporting period	Age (0-7d, 8-28d), sex, outcome (alive, died), health facility type	RHIS patient data: case notes/ charts, Survey/ periodic assessments	Monthly	Adapted from QoC Child[9]	8	12	26	New prioritized quality indicator from WHO SSNB Quality standard 2 "Actionable Information Systems", statement 2.1. to measure quality (Donabedian process) to improve clinical information for real-time clinical decision making and quality improvement activities. This indicator will give insight into person-centred individual care and contribute to data for the other indicators in this set. Cross-cutting quality domains include highlighting available medical records, physical resources, human resource education needs.<br>Survive and Thrive: day 1-27

Standard	Indicator Name	Indicator definition	Classification (based Paed QoC, otherwise MoNITOR)	Health facility service Level (ward) for measurement	Numerator	Denominator	Proposed			Prioritized list alignment and definition	Online consultation survey ranking			Rationale
							Disaggregation	Data source	Report frequency		Useful rank	Core rank	Measure 2y rank	
3	Normothermia on admission	% of neonates with an admission temperature 36.5-37.5 °C on admission to the neonatal unit	Outcome<br><br>*(Donabedian = outcome)*	Neonatal unit	# of neonates who arrive on the neonatal unit with an admission temperature 36.5-37.5 °C	# of neonates admitted to the neonatal unit	inborn/ outborn, outcome (alive, died), health facility type	RHIS patient data: patient charts/ case notes, registers	Monthly	New from WHO SSNB Quality Standards[12]	29	24	16	New prioritized quality indicator from WHO SSNB Quality standard 3 "Functional referral systems", statement 3.5 (Donabedian outcome) to measure a preventable condition directly related to newborn survival for all small and/or sick newborns. This indicator will give insight into coverage of provision of care before and during transfer within the health facility as well from home or when born in a lower-level health facility. Cross-cutting quality domains include highlighting available medical records, physical resources, human resource education needs.<br>Survive and Thrive: day 1-27
4	Use of SSNB quality-of-care indicators - displayed on neonate unit	% of neonatal units that publicly display trends for ≥3 SSNC QoC indicators accessible to health facility staff and families	Process/ Output (facility level)<br><br>*(Donabedian = process)*	Neonate unit	# of neonatal units that publicly display trends for >3 SSNC QoC indicators accessible to health facility staff and families	# of health facilities with neonatal units assessed during the reporting period	health facility type,	HFA/ District supervisory survey	Quarterly	Adapted from QoC Child[9] to align with WHO SSNB Quality Standards[12]	Not in survey			New prioritized quality indicator (Donabedian process) from WHO SSNB Standard 4 "Effective communication and meaningful participation" to measure evidence of family-centred data use that is standard quality improvement practice in low mortality settings. This indicator will give insight for tracking into three or more SSNB Quality of care indicators, contextualized for the setting. The display promotes accountability to the community – both families, and health professionals. This display can also connect frontline neonatal units with health facility and sub-national management team. Cross-cutting quality domains include highlighting the topic of the selected quality indicator, actionable information systems, human resources education needs.<br>Survive and Thrive: day 1-27

Standard	Indicator Name	Indicator definition	Classification (based Paed QoC, otherwise MoNITOR)	Health facility service Level (ward) for measurement	Numerator	Denominator	Proposed			Prioritized list alignment and definition	Online consultation survey ranking			Rationale
							Disaggregation	Data source	Report frequency		Useful rank	Core rank	Measure 2y rank	
5	Death notification/ registration in civil registration and vital statistics system	% of institutional neonatal deaths notified/ registered*** to the appropriate civil authority	Process / Output (facility level) *(Donabedian = process)*	All wards	# of institutional neonatal deaths notified/ registered*** to the appropriate civil authority	# of neonates admitted to the neonatal unit and die during admission between day 0-27	health facility type,	RHIS patient data: patient charts/ case notes, registers, death review/ audit	Monthly	New from WHO SSNB Quality Standards[12]	9	17	22	New prioritized quality indicator from WHO SSNB Quality standard 5 "respect, protection and fulfilment of newborn rights and preservation of dignity", statement 5.5 (Donabedian process) to measure SSNB have their birth registered and has an identity. This indicator will give insight into adherence of this priority action aligning health facilities with civil registration and vital statistics platforms. Cross-cutting quality domains include human resources education needs. Survive and Thrive: day 1-27
6	Developmentally supportive care	% of neonates admitted to the neonatal unit who experience skin-to-skin care (called KMC only if low birthweight) for two or more hours daily during admission	Process/ Output (patient level) *(Donabedian = process)*	Neonatal unit	# of neonates who experienced skin-to-skin care (called KMC only if low birthweight) for two or more hours of daily during admission	# of neonates admitted to the neonatal unit	sex, stratified (<1000g, 1000-1499g, 1500-1999g, 2000-2499g (all KMC), 2500-3999g, >4000g), inborn/ outborn, outcome (alive, died), health facility type,	RHIS patient data: patient charts/ case notes, registers	Monthly	New from WHO SSNB Quality Standards[12] and Nurturing Care Framework[15]	Not in survey			New prioritized quality indicator from WHO SSNB Quality standard 6 "Emotional, psychosocial, and developmental support", statement 6.3 (Donabedian process) to measure SSNBs receive developmentally supportive care with their families as recognized partners in care. This indicator will give insight into adherence to this functional expression developmentally supportive care for the SSNB during admission and the priority action of zero separation. Skin-to-skin care is for all babies for 2 hours per days. Continuous skin-to-skin care when the newborn is low birth weight is defined as KMC in this set. The indicator data has potential to be used to accelerate transforming health facility environments to promote infant and family centred developmental care. Cross-cutting quality domains include physical resources to enable this evidence-based practice and human resources availability education needs. Survive and Thrive: day 1-27

Standard	Indicator Name	Indicator definition	Classification (based Paed QoC, otherwise MoNITOR)	Health facility service Level (ward) for measurement	Numerator	Denominator	Proposed			Prioritized list alignment and definition	Online consultation survey ranking			Rationale
							Disaggregation	Data source	Report frequency		Useful rank	Core rank	Measure 2y rank	
6	Follow-up for growth and development	% of neonates discharged from the neonatal unit who received timely risk-appropriate†† † follow-up as per discharge plan	Process/ Output (patient level)<br><br>*(Donabedian = process)*	Neonatal unit	# of neonates discharged from the neonatal unit who received timely risk-appropriate††† follow-up as per discharge plan	# of neonates discharged from the neonatal unit with identified risks	sex, stratified (<1000g, 1000-1499g, 1500-1999g, 2000-2499g, 2500-3999g, >4000g)	RHIS patient data Parent Report e.g. phone survey	Monthly	New from WHO SSNB Quality Standards[12]	17 adapt	23 adapt	27 adapt	New prioritized quality indicator from WHO SSNB Quality standard 6 "Emotional, psychosocial, and developmental support", statement 6.5 (Donabedian process) to measure interventions to promote thrive that directly addresses the high post-discharge mortality and morbidity for SSNB. This indicator will give insight into coverage of provision of care for early intervention to prevent the two major risks to SSNB thriving – malnutrition and developmental delay. The indicator data has potential for use for a set of actions at health systems, health facility, physical and human resources, clinical and educational planning, and actionable information systems with longitudinal data, for actions including intensive breast and complementary feeding support and early physiotherapy and occupational therapy. Cross-cutting quality domains include highlighting available medical records, equipment, human resources education needs. Survive and Thrive: day 1-27

Standard	Indicator Name	Indicator definition	Classification (based Paed QoC, otherwise MoNITOR)	Health facility service Level (ward) for measurement	Numerator	Denominator	Proposed			Prioritized list alignment and definition	Online consultation survey ranking			Rationale
							Disaggregation	Data source	Report frequency		Useful rank	Core rank	Measure 2y rank	
7	Nurse/ baby ratio on neonate unit	% of neonatal units regularly reporting nurse/baby ratios for every shift by type of ward	Input<br>*(Donabedian = structure)*	Neonatal unit	# of neonatal units regularly reporting nurse/baby ratios for every shift by type of ward	# of health facilities with neonatal units assessed during the reporting period	Neonatal unit type (intensive, high-dependency, special care, standard inpatient, KMC units, or by patient acuity, shift (morning, afternoon, night etc.)	RHIS facility data for newborn admissions, nursing roster to capture nurses per shift	Monthly	New from WHO SSNB Quality Standards[12]	Not in survey			New prioritized quality indicator from WHO SSNB Quality standard 7 "Competent motivated human resources", statement 7.1 (Donabedian input) to measure each health facility has adequate number of competent, motivated, empathetic, multi-disciplinary staff available with SSNB skills. This indicator will give insight into neonatal unit level appropriate SSNB skilled staffing ratios to enable SSNB and their families to receive optimal quality care that achieve desirable patient outcomes.<br>Survive and Thrive: day 1-27
8	Hand hygiene at facility unit entrance	% of neonatal units which have functioning (water and soap) hand hygiene station at the entrance	Input<br>*(Donabedian = structure)*	Neonatal unit	# of neonatal units with functioning (water and soap) hand hygiene station at the entrance	# of health facilities with neonatal units assessed during the reporting period	health facility type	HFA/ District supervisory survey	Quarterly	Adapted MoNITOR[6], QoC MNH[8]	10	9	5	New prioritized quality indicator from WHO SSNB Quality standard 8 "Essential physical resources", statement 8.2 (Donabedian input) to measure each health facility has availability of an actionable critical commodity of functioning infrastructure to provide adequate WASH for SSNB care. This indicator directly addresses the input to enable family and health professional behaviour to prevent one of the top three causes of neonatal mortality/morbidity.<br>Survive and Thrive: day 1-27

Standard	Indicator Name	Indicator definition	Classification (based Paed QoC, otherwise MoNITOR)	Health facility service Level (ward) for measurement	Numerator	Denominator	Proposed			Prioritized list alignment and definition	Online consultation survey ranking			Rationale
							Disaggregation	Data source	Report frequency		Useful rank	Core rank	Measure 2y rank	
8	Stockout of safe oxygen delivery systems¶¶¶	% of neonatal units with no stockout of safe oxygen delivery systems¶¶¶ in a specified period.	Input<br>*(Donabedian = structure)*	Neonatal unit	# of neonatal units reporting no stockout of safe oxygen delivery systems¶¶¶ in a specified period	# of health facilities with neonatal units assessed during the reporting period	health facility type, type of stock out	RHIS facility data: Inventory	Monthly	Adapted Health Facility Indicator[7]	11	15	8	New prioritized quality indicator from WHO SSNB Quality standard 8 "Essential physical resources", statement 8.3 (Donabedian input) to measure health facility continuous availability of an actionable critical commodity to enable newborns to survive and thrive. This indicator will give insight into supply chains for safe oxygen which requires adjustable FiO2 titrated by pulse oximetry on the baby. This Indicator needs to be considered alongside the balancing indicator measuring retinopathy of prematurity screening.<br>Survive and Thrive: day 1-27
8	Stockouts of three essential neonate tracer medicines	% of neonatal units reporting no stockout of three essential tracer medicines in correct formulations in a specified period	Input<br>*(Donabedian = structure)*	Neonatal unit	# of neonatal units reporting no stockout of three essential tracer medicines in correct formulations:<br>1. first-line injectable antibiotics,<br>2. phenobarbital<br>3. caffeine (or other methylxanthines)	# of health facilities with neonate units assessed during the reporting period	health facility type, type of medication	RHIS facility data: Inventory	Quarterly	Adapted QoC Child[9] and Health Facility Indicator[7]	13	11	14	New prioritized quality indicator from WHO SSNB Quality standard 8 "Essential physical resources", statement 8.3 (Donabedian input) to measure health facility continuous availability of an actionable critical commodity to enable newborns to survive and thrive. This indicator will give insight into supply chains for drugs with newborn formulations. This Indicator needs to be considered alongside the balancing indicator measuring retinopathy of prematurity screening.<br>Survive and Thrive: day 1-27

Standard	Indicator Name	Indicator definition	Classification (based Paed QoC, otherwise MoNITOR)	Health facility service Level (ward) for measurement	Numerator	Denominator	Proposed			Prioritized list alignment and definition	Online consultation survey ranking			Rationale
							Disaggregation	Data source	Report frequency		Useful rank	Core rank	Measure 2y rank	
8	Stock outs of three[≠] essential neonatal tracer devices, equipment and supplies	% of neonatal units reporting no stockout of three¶ functioning essential neonatal tracer devices, equipment and supplies in a specified period	Input<br><br>*(Donabedian = structure)*	Neonatal unit	# of neonatal units reporting no stockout of three¶ essential neonatal tracer devices, equipment and supplies in a specified period:<br>1.digital scales<br>2.phototherapy<br>3.point-of-care glucose testing	# of health facilities with neonatal units assessed during the reporting period	health facility type, type of device	RHIS facility data: Inventory	Quarterly	Adapted Health Facility Indicator[7]	28	32	28	New prioritized quality indicator from WHO SSNB Quality standard 8 "Essential physical resources", statement 8.4 (Donabedian input) to measure each health facility has availability of equipment essential to all neonatal units providing SSNB care to enable newborns to survive and thrive. This indicator will give insight into supply chains and engineers available to ensure continuous supply of functioning devices. For level 2 plus units and level 3 units, continuous positive airways pressure (CPAP) as a fourth tracer device can be added for tracking.<br>Survive and Thrive: day 1-27
Facility /individual level outcome	Exclusive breast-milk feeding at time of discharge	% of neonates exclusively breastmilk fed (sucking, cup/ tube fed) at discharge from the neonate unit	Outcome<br><br>*(Donabedian = outcome)*	Neonatal unit	# of infants discharged from the neonatal unit fed exclusively with breast milk in the preceding 24 hours	# of infants discharged from the neonatal unit	health facility type, weight <1000g, 1000-1499g, 1500-1999g, 2000-2499g, 2500--3999g, >4000g), inborn/ outborn	RHIS patient data: patient charts/ case notes, registers	Monthly	Adapted from 100 Prioritized health indicators[16], WHO SSNB Quality Standards[12], Child Health and wellbeing[17]	1	6	2	New prioritized quality indicator from WHO SSNB Quality standard 1 "Evidence-based practices", statement 1.3. (Donabedian outcome) as a summative outcome with enduring implications for maternal and newborn health. This indicator will give insight into the support for lactation and breastfeeding in each health facility providing SSNB care, which includes cross-cutting quality domain of competent motivated human resources and private physical spaces to express.<br>Survive and Thrive: day 1-27

Standard	Indicator Name	Indicator definition	Classification (based Paed QoC, otherwise MoNITOR)	Health facility service Level (ward) for measurement	Numerator	Denominator	Proposed			Prioritized list alignment and definition	Online consultation survey ranking			Rationale
							Disaggregation	Data source	Report frequency		Useful rank	Core rank	Measure 2y rank	
**Facility /individual level outcome**	Neonatal survival rate after inpatient care	% of neonatal survivors by birthweight category at facility discharge after admission to the neonatal unit	Impact<br>*(Donabedian = outcome)*	Neonatal unit	# of infant survivors by birthweight category at facility discharge after admission to the neonatal unit	# of infants admitted to the neonatal unit day 0-27 by birth weight categories	age: 0-7d, 8-28d, sex, stratified <1000g, 1000-1499g, 1500-1999g, 2000-2499g, 2500-3999g, >4000g, inborn/ outborn	RHIS patient data: patient charts/ case notes, registers, death review/ audit	Quarterly	New from WHO SSNB Quality Standards[12]	15	15	16	New prioritized quality indicator (Donabedian outcome) to measure survival among admitted SSNB care stratified by birthweight. This indicator will give insight into the performance of the neonatal unit, when adjusted for case mix and health facility level. The indicator data has potential for use for a set of actions at health systems, health facility, physical and human resources, clinical and educational planning for actions including intrapartum care.<br>Survive and Thrive: day 1-27
**Facility /individual level outcome**	Infant and family-centred neonatal care	*to be defined – proposed topic content see Supplemental material 16*	Outcome<br>*(Donabedian = outcome)*	Neonatal unit	*to be defined*	*to be defined*	*to be defined*	Survey: Parent interview	Quarterly	New from [SSNB Standards[8]]	Not in survey			*to be defined*
**Facility /individual level outcome**	Person-centred provider care	*to be defined – proposed topic content see Supplemental material 17*	Outcome<br>*(Donabedian = outcome)*	Neonatal unit	*to be defined*	*to be defined*	*to be defined*	Survey: Provider interview	Quarterly	New from [SSNB Standards[8]]	Not in survey			*to be defined*
**Facility /individual level outcome**	Neonatal thrive rate by birth weight group category *indicator*	*to be defined*	Impact<br>*(Donabedian = outcome)*	Neonatal unit	*to be defined*	*to be defined*	*to be defined*	RHIS patient data: patient charts/ case notes, registers, longitudinal data	Quarterly	New from [SSNB Standards[8]]	Not in survey			*to be defined*

Abbreviations:

Standard= WHO SSNB Quality Standards[5]
# = number
SSNB = small and/or sick newborns
QoC = Quality-of-care
ICD = International Classification of Diseases
MoNITOR = Mother and Newborn Information for Tracking Outcomes and Results WHO Technical Advisory Group

Footnotes:

Standard	WHO SSNB Quality Standards
#	Number
*	*Indicators in table 1A are adopted from previously prioritised global indicator lists, referenced in the table
~	Excluding macerated stillbirths
†	Kangaroo mother care is defined by WHO as early, continuous, and prolonged skin-to-skin contact between the mother (or other caregiver) and the baby, and exclusive breastfeeding.[18]
‡	Clinically suspected sepsis (serious bacterial infection). Danger signs include: not feeding well, convulsions, drowsy or unconscious, movement only when stimulated or no movement at all, fast breathing (60 breaths per minute), grunting, severe chest in-drawing, raised temperature: >38 °C, hypothermia: <35.5 °C,. Localizing signs of infection are: signs of pneumonia, many or severe skin pustules, umbilical redness extending to the peri-umbilical skin, umbilicus draining pus, bulging fontanelle, painful joints, joint swelling, reduced movement and irritability if these parts are handled[19]
§	known to be alive on admission to the facility[4]
‡‡‡	196 or more days gestation (≥28+0 weeks)[4]
**	weekly visits or contact as needed with for smaller units
††	indirect ophthalmologic examination or with imaging and remote grading by trained ophthalmologists / technicians / neonatal nurses, as appropriate,
‡‡	laser by indirect delivery or intravitreal injection by an ophthalmologist
§§	temperature, respiratory rate, weight, feeding status
¶¶	classification / diagnosis, treatment, counselling, care outcomes (weight, feeding status and place/ time of follow-up, link to follow-up plans)
***	context specific depends on legal frameworks to register deaths
†††	context specific risk management by neonatal condition.
¶¶¶	adjustable FiO2 using pulse oximetry
¶	for level 2 plus units and level 3 units, continuous positive airways pressure (CPAP) as a fourth tracer device can be added for tracking

Supplemental Material 14: Reporting standards for guideline-based performance measure development and re-evaluation[20]

**Criteria**	
**1. Guideline selection**	
1a. State the currency of the guideline(s) used for guideline-based performance measure development and state if it/they meet the criteria set out by the Guidelines International Network (G-I-N). Describe the guideline quality using a validated guideline appraisal tool, such as AGREE II.	World Health Organization. Standards for improving the quality of care for small and sick newborns in health facilities 2020. https://apps.who.int/iris/bitstream/handle/10665/334126/9789240010765-eng.pdf (accessed 23 November 2020).[8]<br><br>**Chapter 6: Development of the standards**<br>"To develop standards specific for small and sick newborns, existing evidence, guidelines and standards of care were reviewed to identify shortcomings. The literature review covered all WHO guidelines and recommendations for evidence-based practices in newborn health, published literature on standards of care for small and sick newborns, international best practices, standards and guidelines and *Lancet* series on topics that included maternal, newborn and child health. The Cochrane Database of Systematic Reviews on neonatal health topics was reviewed, and the website of the National Institute for Health and Care Excellence was searched for clinical guidelines and quality standards relevant to newborns. When gaps were identified, PubMed searches were conducted for up-to-date systematic reviews on relevant topics. The website of the European Standards of Care for Newborn Health project, an interdisciplinary European collaboration to develop standards of care for 11 areas in newborn health, was reviewed to determine its relevance to global standards of care for small and sick newborns.<br><br>**6.1 Exclusion criteria**<br>Ending preventable newborn deaths requires interventions delivered throughout the continuum of care, with high-quality care during labour, around birth and the first week of life and care for small and sick newborns. Care options given to the mother to improve newborn outcomes have been provided in WHO maternal health guidance documents addressing preterm birth outcomes and antenatal and intrapartum care for a positive childbirth experience. Interventions given to women in preterm labour that are published as standards include antenatal corticosteroids for preterm birth at 24–34 weeks' gestation, magnesium sulfate at birth before 32 weeks' gestation and antibiotics in preterm pre-labour rupture of membranes. Other relevant maternal interventions include maternal nutrition, maternal iron and folic acid supplementation, tetanus toxoid vaccination and delayed umbilical cord clamping. These interventions were not included in the standards for small and sick newborns.<br>**6.2 Findings**<br>Existing WHO guidance provides numerous standards and recommendations for the provision and experience of care. These include evidence-based care of small and sick newborns in domain 1 and other standards in domains 2–8 (see Fig. 1). The findings of the literature review were mapped to existing WHO guidance, and topics important for ensuring high-quality standards for small and sick newborns in health facilities under each of the eight domains were listed to identify gaps in WHO guidance.

	In summary, most evidence-based interventions were already included in WHO standards and recommendations for special care, but additional special care and neonatal intensive care interventions for small and sick newborns were required. There was limited advice on surveillance, prevention and management of important neonatal problems, including congenital abnormalities and retinopathy of prematurity, and on general and neurodevelopmental follow-up and screening. Gaps were found particularly in the newborn's experience of care, including newborn participation, newborn rights and respectful care.<br>Standards, quality statements and quality measures specific for small and sick newborns were drafted to augment existing WHO standards and to fill any gaps. Several drafts were prepared and shared with experts for external review, and a technical meeting was held on 10–12 April 2019 in Geneva. A final draft was prepared with input from that meeting and from WHO technical units.<br>**6.3 Quality measures**<br>When the quality measures in the *Standards for improving quality of maternal and newborn care in health facilities* (2016) and the *Standards for improving the quality of care for children and young adolescents in health facilities* (2018) were considered sufficient for small and sick newborns, they underwent minor modification to make them more applicable.<br>When new quality measures were required specifically for small and sick newborns, these were provided. All new quality statements were provided with new quality measures.<br>The standards, quality statements and quality measures for small and sick newborns include interventions to level 3 care for low- and middle-income countries. They do not include standards for high-technology interventions provided in high-income countries, which are listed in textbooks and clinical guidelines for neonatal intensive care"
1b. Indicate additional sources, if used and the rationale for their use.	Existing global indicator lists, referenced in methods step 2
**2. Selection of guideline recommendations**	
State the strength of evidence and/or the grade of recommendation qualifying the guideline recommendations to be used for guideline-based performance measures.	Existing indicator criteria, referenced in methods step 3
**3. Selection process of performance measures from guideline recommendations**	
Describe clearly and in detail the methods used to develop the performance measures from the supporting clinical guideline recommendations.	Methods steps 1-4
**4. Core attributes of performance measures**	
State, if the following attributes within the development process of guideline-based performance measures were considered:<br>• Relevance (as a minimum: potential for improvement/clinical relevance)	Methods steps 1-4, Supplemental material 1-7

• Scientific Soundness (as a minimum: the evidence supporting the measure)<br>• Feasibility (as a minimum: clarity of definition and measurability)	
**5. Specification of performance measures**	
State that numerator and denominator of the guideline-based Performance Measure is specified unambiguously and in detail.	Table 1
**6. Intended use of performance measures**	
State if there is a clear description of the intended use of the performance measure (quality improvement, quality assurance with or without accountability purposes, pay for performance) and at what level in the health system it is used (local, regional, national).	Methods step 1
**7. Practice test of performance measures**	
If a practice test (piloting) is carried out prior using the guideline-based performance measure, provide a full description of the process.<br>If no practice test is done, provide the rationale for this.<br>Provide information about any other validation process in use.	Beyond the scope of this study, recommended as next steps
**8. Review and re-evaluation of performance measures**	
Report the currency of the performance measures in use.<br>State if there are criteria for deciding to change or stop using performance measures.	No existing global list – Introduction
**9. Composition of the panel deciding on guideline-based performance Measures**	
Describe clearly the composition of the panel deciding on guideline-based performance measures with information on participation of multidisciplinary experts, stakeholders in the field, experts in quality measurement, and patient representatives.	Supplemental Material 1: Composition of the SSNB QoC Indicators Technical Working Group

Supplemental Material 15: Proposed parent survey indicators to include in an infant and family-centred newborn care score for neonatal unit, covering standards 1 to 8. Score requires defining and context validation (n=16)

Standar	Indicator Name	Classification (Paed QoC, otherwise MONITOR)	Service Level for measurement - ward	Proposed data source	Proposed measurement method	Proposed measurement frequency	CORE list summary and definition	Online survey ranking		
								Useful rank	Core rank	Measure 2y rank
**Parent report of quality domains**										
1	Knowledge of danger signs, when to seek care, illness feeding	Outcome (patient report)	Focus newborn unit	Survey	Parent interview	quarterly	QoC Child[34]	27	21	40
2	Usefulness of child home-based records	Outcome (patient report)	Focus newborn unit	Survey	Parent interview	quarterly	None, proposed requires testing	-	-	-
3	Enabled to attend follow up for growth/ development	Outcome (patient report)	Focus newborn unit	Survey	Parent interview	quarterly	None, proposed requires testing	-	-	-
4	Parent knowledge and understanding of newborn's condition/ treatment plan	Outcome (patient report)	Focus newborn unit	Survey	Parent interview	quarterly	QoC Child[34]	38	31	50
5	Experienced continuous access to baby	Outcome (patient report)	Focus newborn unit	Survey	Parent interview	quarterly	[SSNB Standards[8]]	39	43	47
5	Experienced compassion and respect	Outcome (patient report)	Focus newborn unit	Survey	Parent interview	quarterly	[SSNB Standards[8]]	35	39	44
5	Disrespectful care for the newborn	Outcome (patient report)	Focus newborn unit	Survey	Parent interview	quarterly	QoC Child[34]	43	36	48
5	Received best possible care at the facility	Outcome (patient report)	Focus newborn unit	Survey	Parent interview	quarterly	None, proposed requires testing	50	49	51
8	Recommend health facility newborn care to other families	Outcome (patient report)	Focus newborn unit	Survey	Parent interview	quarterly	None, proposed requires testing	51	51	50
5	Child rights awareness	Outcome (patient report)	Focus newborn unit	Survey	Parent interview	quarterly	QoC Child[34]	48	41	46
5	Birth notification/ registration in civil registration and vital statistics system after facility birth	Outcome (patient report)	Focus newborn unit	Survey	Parent interview	quarterly	[SSNB Standards[8]]	31	25	24
6	Satisfaction with accompaniment during care/ rooming in	Outcome (patient report)	Focus newborn unit	Survey	Parent interview	quarterly	QoC Child[34]	36	34	43
6	Satisfaction with decision-making process for care	Outcome (patient report)	Focus newborn unit	Survey	Parent interview	quarterly	QoC Child[34]	41	38	37
8	Adequate space for family centred care, KMC	Outcome (patient report)	Focus newborn unit	Survey	Parent interview	quarterly	[SSNB Standards[8]]	49	48	52
8	Hand hygiene and WASH amenities	Outcome (patient report)	Focus newborn unit	Survey	Parent interview	quarterly	[SSNB Standards[8]]	45	45	36
8	Privacy for expressing breast milk	Outcome (patient report)	Focus newborn unit	Survey	Parent interview	quarterly	[SSNB Standards[8]]	37	42	30

Proposed - included in online survey n = 8 (yellow), Proposed - not included in online survey n = 2 (orange), Adapted from already recommended core indicators n=6 (dark pink).

Supplemental Material 16: Proposed provider survey indicators to include in a person-centred provider care score for neonatal unit, covering standards 1 to 8. Score requires defining and context validation (n=9)

Standard	Indicator Name	Classification (Paed QoC, otherwise MONITOR)	Service Level for measurement - ward	Proposed data source	Proposed measurement method	Proposed measurement frequency	CORE list summary and definition	Online survey ranking		
								Useful rank	Core rank	Measure 2y rank
**Provider report of quality domains**										
1	Skills/ knowledge: Evidence based practices including KMC, rationale use oxygen/ CPAP, Phototherapy, antibiotics/ medications, follow up etc	Process / Output	Focus newborn unit	Survey	Provider interview	quarterly	[SSNB Standards[8]]	-	-	-
2	Newborn data use literacy/ data quality	Process / Output	Focus newborn unit	Survey	Provider interview	quarterly	None?	-	-	-
3	Supported in referral decisions	Process / Output	Focus newborn unit	Survey	Provider interview	quarterly	[SSNB Standards[8]]	-	-	-
4	Skills/ knowledge: Effective communication	Process / Output	Focus newborn unit	Survey	Provider interview	quarterly	[SSNB Standards[8]]	-	-	-
5	Skills/ knowledge: Rights, bereavement respect	Process / Output	Focus newborn unit	Survey	Provider interview	quarterly	[SSNB Standards[8]]	-	-	-
6	Skills/ knowledge: Family-centred care	Process / Output	Focus newborn unit	Survey	Provider interview	quarterly	[SSNB Standards[8]]	-	-	-
7	Clinical Quality Improvement mentorship or training	Input	Focus newborn unit	Survey	Provider interview	quarterly	QoC Child[34]	32	28	23
8	Hand hygiene and WASH amenities	Input	Focus newborn unit	Survey	Provider interview	quarterly	[SSNB Standards[8]]	-	-	-
8	Teamwork, workplace trust	Process / Output	Focus newborn unit	Survey	Provider interview	quarterly	[SSNB Standards[8]]	-	-	-

Proposed - not included in online survey n = 8 (orange), Adapted from already recommended core indicators n=8 (dark pink).

Supplemental Material 17: Proposed Additional SSNB quality Indicators – covering standards 1 to 8 (n=8)

Standard	Indicator Name	Classification (Paed QoC, otherwise MONITOR)	Service Level for measurement - ward	Proposed data source	Proposed measurement method	Proposed measurement frequency	CORE list summary and definition	Online survey ranking		
								Useful rank	Core rank	Measure 2y rank
(1)	Availability of equipment and supplies - respiratory - CPAP functioning	Input	Focus newborn unit	HFA/ District supervisory survey	Observation	quarterly	ENAP coverage target[28],	12	19	11
(1)	Availability of equipment and supplies - Phototherapy functioning	Input	Focus newborn unit	HFA/ District supervisory survey	Observation	quarterly	None	25	28	9
1	Vision - Retinopathy of Prematurity proportion eligible screened	Process	Focus newborn unit	RHIS patient data	Patient Register, Case notes	monthly	[SSNB Standards[8] and Vermont Oxford Network Patient level[13,14] discussion with ROP experts LSHTM]]	23	30	39
1	Hearing – proportion of at-risk newborns screened	Process	Focus newborn unit	RHIS patient data	Patient Register, Case notes	monthly	[SSNB Standards[8]	Not in survey		
5	Availability of key care national protocols: Rights, Family-centred care, bereavement respect, CPAP, Phototherapy, rational use antibiotics/ medications	Input	Focus newborn unit	HFA/ District supervisory survey	Observation	quarterly	[SSNB Standards[8]]	26	29	33
7	Health worker density and distribution	Input	Focus newborn unit	Health worker records	RHIS facility data	quarterly	Health Facility Indicator[26]	14	13	18
8	No stock out of any essential newborn medicines from full list	Input	Focus newborn unit	Inventory	RHIS facility data	quarterly	QoC Child[34], Health Facility Indicator[26]	24	33	21
8	No stock out any essential newborn devices, equipment and supplies	Input	Focus newborn unit	Inventory	RHIS facility data	quarterly	Health Facility Indicator[26]	33	37	29
8	No "Stock out" of electrical power	Input	Focus newborn unit	Inventory	RHIS facility data	quarterly	[SSNB Standards[8] and Health Facility Indicator[26]]	42	44	35
	For Research to explore measurability and usability.: proposed output indicators specific for target group provision of care but measured at unit/ward level monthly include provision of "baby-days" for phototherapy, CPAP, and 'togetherness'	Output	unit/ward level	RHIS facility data	RHIS facility data	quarterly	To be developed			

Proposed - included in online survey n = 4 (yellow), adapted from already recommended core indicators n=1 (dark pink).

Supplemental Material 18: Proposed facility effective coverage cascade for SSNB

Illustrative coverage cascades using data elements captured in list of prioritised indicators for a) all small and/or sick newborns and b) preterm/ low-birth weight newborns.[21]

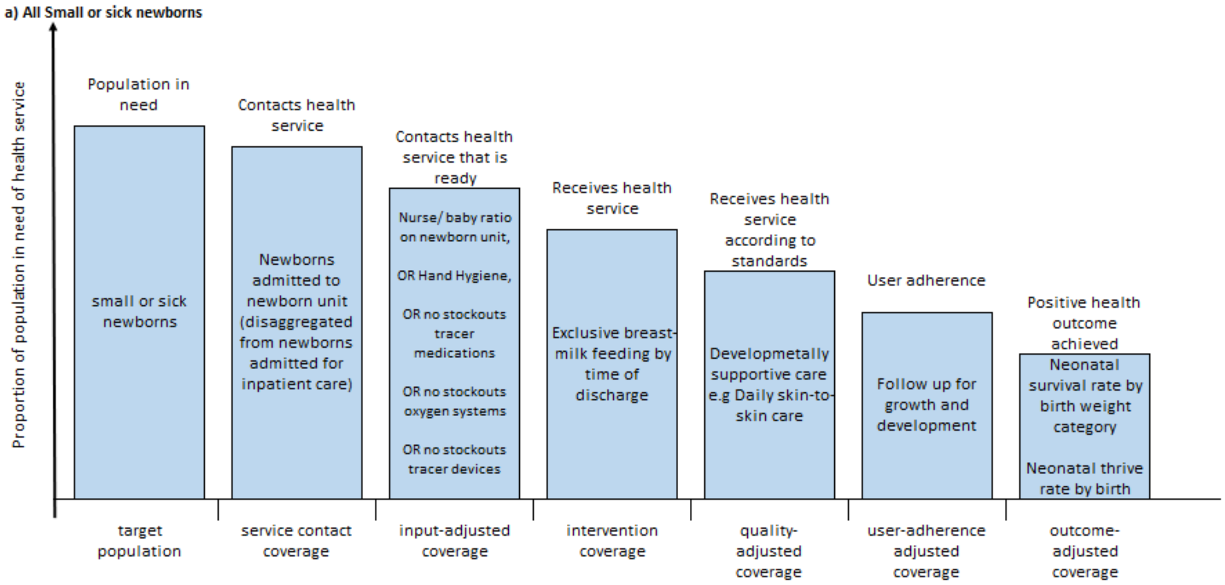

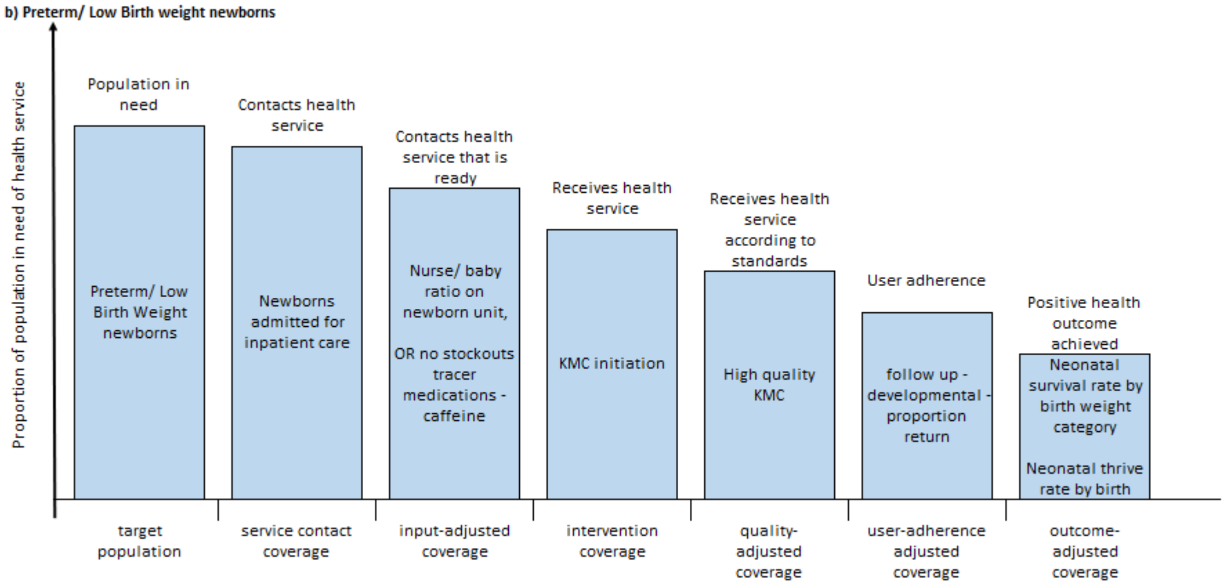

References

1. Mason E, McDougall L, Lawn JE, et al. From evidence to action to deliver a healthy start for the next generation. *The Lancet* 2014; **384**(9941): 455-67.

2. WHO working group on coverage indicators for small and/or sick newborn care. Care for small and/or sick newborns: coverage indicators for measurement in routine health information systems. *[submitted to PLOS ONE]* 2024.

3. World Health Organization. Analysis and use of health facility data: guidance for maternal, newborn, child and adolescent health programme managers2023. https://iris.who.int/bitstream/handle/10665/373826/9789240080331-eng.pdf?sequence=1 (accessed 20 December 2023).

4. Blencowe H, Okwaraji Y, Hug L, You D. Stillbirth Definition and Data Quality Assessment for Health Management Information Systems (HMIS), a guideline.2022. https://data.unicef.org/resources/stillbirth-definition-and-data-quality-assessment-for-health-management-information-systems/ (accessed 13 May 2023).

5. World Health Organization. International Classification of Diseases 11th Revision2022. https://icd.who.int/en (accessed.

6. World Health Organization. Mother and Newborn Information for Tracking Outcomes and Results (MoNITOR) Database Recommended CORE indicators (3 March 2022 version). 2020. https://monitor.srhr.org/ (accessed 3 March 2022).

7. World Health Organization. Analysis and use of Health Facility Data Core Health Facility Indicators 2019. https://cdn.who.int/media/docs/default-source/world-health-data-platform/rhis-modules/facilityanalysisguidance-indicators-2021--01-21.pdf?sfvrsn=76b0be9b_5 (accessed 12 December 2021).

8. Maternal and newborn Health Quality of Care Metrics Technical Working Group. Quality of Care for Maternal and Newborn Health: A monitoring framework for network countries 2019. https://cdn.who.int/media/docs/default-source/mca-documents/qoc/qed-quality-of-care-for-maternal-and-newborn-health-a-monitoring-framework-for-network-countries.pdf?sfvrsn=19a9f7d0_1&download=true (accessed 23 March 2021).

9. Muzigaba M, Chitashvili T, Choudhury A, et al. Global core indicators for measuring WHO's paediatric quality-of-care standards in health facilities: development and expert consensus. *BMC Health Serv Res* 2022; **22**(1): 887.

10. UNICEF, World Health Organization. Ending preventable newborn and stillbirths by 2030: moving faster towards high-quality universal health coverage in 2020–20252020. https://www.unicef.org/media/77166/file/Ending-preventable-newborn-deaths-and-stillbirths-by-2030-universal-health-coverage-in-2020%E2%80%932025.pdf (accessed 24 March 2021).

11. World Health Organization. Analysing and using routine data to monitor the effects of COVID-19 on essential health services: practical guide for national and subnational decision-makers: interim guidance2021. https://apps.who.int/iris/bitstream/handle/10665/338689/WHO-2019-nCoV-essential_health_services-monitoring-2021.1-eng.pdf (accessed 24 March 2021).

12. World Health Organization. Standards for improving the quality of care for small and sick newborns in health facilities 2020. https://apps.who.int/iris/bitstream/handle/10665/334126/9789240010765-eng.pdf (accessed 6 Oct 2024).

13. Edwards EM, Ehret DEY, Soll RF, Horbar JD. Vermont Oxford Network: a worldwide learning community. *Transl Pediatr* 2019; **8**(3): 182-92.

14. Vermont Oxford Network. Manual of Operations: Part 2. Part 1. Guidelines for Database Participation version 4.12022. https://vtoxford.zendesk.com/hc/article_attachments/6539565329299/Manual_of_Operations_Part_1_v4.1.pdf (accessed 26 February 2023).

15. World Health Organization, United Nations Children's Fund, World Bank Group. Nurturing care for early childhood development: a framework for helping children survive and thrive to transform health and human potential2018. https://apps.who.int/iris/bitstream/handle/10665/272603/9789241514064-eng.pdf?ua=1&ua=1 (accessed 28 February 2021).

16. World Health Organization. Global reference list of 100 core health indicators (plus health-related SDGs)2018. https://apps.who.int/iris/bitstream/handle/10665/259951/WHO-HIS-IER-GPM-2018.1-eng.pdf (accessed 28 October 2022).

17. Requejo J, Strong K, Agweyu A, et al. Measuring and monitoring child health and wellbeing: recommendations for tracking progress with a core set of indicators in the Sustainable Development Goals era. *Lancet Child Adolesc Health* 2022; **6**(5): 345-52.

18. World Health Organization. WHO recommendations for care of the preterm or low birth weight infant.2023. https://www.who.int/publications/i/item/9789240058262 (accessed 6 Oct 2024).

19. World Health Organization. Essential Newborn Care Course. https://www.who.int/tools/essential-newborn-care-course (accessed 6 Oct 2024).

20. Nothacker M, Stokes T, Shaw B, et al. Reporting standards for guideline-based performance measures. *Implement Sci* 2016; **11**: 6.

21. Marsh AD, Muzigaba M, Diaz T, et al. Effective coverage measurement in maternal, newborn, child, and adolescent health and nutrition: progress, future prospects, and implications for quality health systems. *The Lancet Global Health* 2020; **8**(5): e730-e6.
