# Supplementary material for: Global prioritised indicators for measuring WHO’s quality-of-care standards for small and/or sick newborns in health facilities: development, global consultation and expert consensus
Source: BMJ Open. 2025 Oct 20;15(10):e100338. doi: 10.1136/bmjopen-2025-100338 (PMC12542562; doi:10.1136/bmjopen-2025-100338)
Supplement: Supplementary file 2 [file bmjopen-2025-100338.reviewer_comments.pdf]

PEER REVIEW HISTORY

BMJ Open publishes all reviews undertaken for accepted manuscripts. Reviewers are asked to complete a checklist review form and are provided with free text boxes to elaborate on their assessment. These free text comments are reproduced below.

ARTICLE DETAILS

Title (Provisional)

Global prioritised indicators for measuring WHO's quality-of-care standards for small and/or sick newborns in health facilities: development, global consultation, and expert consensus

Authors

Day, Louise-Tina; Vaz, Lara; Semrau, Katherine EA; Moxon, Sarah; Niermeyer, Susan; Khadka, Neena; Chitashvili, Tamar; Valentine, Gregory C; Drake, Mary; Ehret, Danielle; Sheffel, Ashley; Sacks, Emma; Greenspan, Leah; Shaver, Theresa; Kak, Lily; Hailegebriel, Tedbabe; Gupta, Gagan; Hill, Kathleen; Jackson, Debra

---

VERSION 1 - REVIEW

**Reviewer**	**1**
**Name**	**Lachman, Peter**
**Affiliation**	**Royal College of Physicians of Ireland, Quality**
**Date**	**15-Apr-2025**
**COI**	**None**

This is an important paper that address an area of concern for those in who want to improve the quality care of newborn infants.

I am pleased to see the pragmatic approach has been adopted to ensure that the indicators are feasible to implement. Implementation is the key.

Now that they have been identified a guide on implementation is important i.e. how to train staff to collect the indicators and then to implement improvement initiatives.

**Reviewer**	**2**
**Name**	**Vallely, Lisa**
**Affiliation**	**UNSW, Kirby Institute -Faculty of Medicine**
**Date**	**01-May-2025**
**COI**	**None**

It was a pleasure to read this important paper.

**Reviewer**	**3**
**Name**	**Manalili, Kimberly**
**Affiliation**	**University of Calgary, Community Health Sciences**
**Date**	**08-May-2025**
**COI**	**None**

Thank you for this opportunity to review the manuscript "Global prioritised indicators for measuring WHO's qualityof-care standards for small and/or sick newborns in health facilities: development, global consultation, and expert consensus." The study is rigorous and consistent with standards for quality indicator development.

Overall, the manuscript is well written and concise. With responses obtained from stakeholders (including parents), it would be helpful to include a comment on ethical considerations, even if ethics approval is not needed for this type of study.

Additionally, some details regarding Phase 2 stakeholder consultations would provide clarity on how stakeholders were recruited. How were stakeholders identified and approached? What strategies were used (if any) to ensure representativeness from the different stakeholder groups, particularly as the study limitations indicate few responses from parents.

Can the authors provide additional details about the data analysis for any comments provided through the stakeholder consultations (comment boxes, qualitative data). How were these incorporated into revisions? Additionally, what was the process for making revisions - how was consensus attained through the working group for wording changes and any other revisions that may have been made?

Great discussion section. One consideration I have is to include some comments around the indicator implementation and potential considerations. There have been challenges with adoption and implementation of these types of global indicators. What are some considerations for local adaptation, particularly for resource-constrained settings, which may facilitate the uptake of these indicators to ensure equitable delivery in quality of care for SSNB.

VERSION 1 - AUTHOR RESPONSE

Reviewer 1: Dr. Peter Lachman, Royal College of Physicians of Ireland

**Comments to the author**<br>This is an important paper that address an area of concern for those in who want to improve the quality care of newborn infants.<br><br>I am pleased to see the pragmatic approach has been adopted to ensure that the indicators are feasible to implement. Implementation is the key.<br><br>Now that they have been identified a guide on implementation is important i.e. how to train staff to collect the indicators and then to implement improvement initiatives.	Thank you and we strongly agree, implementation is key. We are grateful for this suggestion and have added into the discussion in the section "path ahead": "Development of an implementation guide for the primary audience will be important including suggestions to overcome barriers to enable high quality data and using the indicators to improve SSNB quality of care." We also note that the WHO commentary under preparation and intended for publication as a complement addresses implementation.
**Reviewer 2 Dr. Lisa Vallely, UNSW**	
**Comments to the Author:**<br>It was a pleasure to read this important paper.	Thank you.
**Reviewer 3: Dr. Kimberly Manalili, University of Calgary**	
**Comments to the Author:** Thank you for this opportunity to<br>review the manuscript "Global prioritised indicators for measuring WHO's quality-of-care standards for small and/or sick newborns in health facilities: development, global consultation, and expert<br>consensus." The study is rigorous and consistent with standards for quality indicator development.	Thank you.

Overall, the manuscript is well written and concise. With responses obtained from stakeholders (including parents), it would be helpful to include a comment on ethical considerations, even if ethics approval is not needed for this type of study.	Thank you for your review. We have included in the ethics section "Respondents to the online stakeholder consultation gave voluntary informed written consent prior to recruitment for observation and again for exit survey. This study was granted ethical approval by the London School of Hygiene & Tropical Medicine Observational research ethics committee, reference 26438." We have added in the link to Supplementary Material 8.
Additionally, some details regarding Phase 2 stakeholder consultations would provide clarity on how stakeholders were recruited. How were stakeholders identified and approached? What strategies were used (if any) to ensure representativeness from the different stakeholder groups, particularly as the study limitations indicate few responses from parents.	Many thanks. We have added to the Phase 2 methods "Email survey invitations were sent purposively to WHO and UNICEF regional and country offices for circulation to ministries of health, health workers/ professional organizations, implementing partners and linked WHO technical advisory groups. The TWG also circulated the survey to researchers and independents experts. All recipients were requested to forward the link to any partners they considered would be interested<br>in providing feedback (Supplementary material 8)".

Can the authors provide additional details about the data analysis for any comments provided through the stakeholder consultations (comment boxes, qualitative data). How were these incorporated into revisions? Additionally, what was the process for making revisions - how was consensus attained through the working group for wording changes and any other revisions that may have been made?	Many thanks for this review. We considered the text responses from respondents as part of our iterative discussions; our rationale is detailed in Table 1. We have added these details to the description of the Phase 2 methodology.
Great discussion section. One consideration I have is to include some comments around the indicator implementation and potential considerations. There have been challenges with adoption and implementation of these types of global indicators. What are some considerations for local adaptation, particularly for resource-constrained settings, which may facilitate the uptake of these indicators to ensure equitable delivery in quality of care for SSNB.	Many thanks for this comment. We agree and have added into the discussion in the section "Path Ahead": " The development of an implementation guide for the primary audience (subnational and facility level programme managers) will be important; it should include suggestions to overcome barriers to enable high quality data and using the indicators to improve SSNB quality of care" In the strengths and limitations section, we updated a sentence to read "We acknowledge this complexity and the importance of testing and experience in use to understand enablers to data collection and use. This information will contribute to revising these prioritised indicators as it becomes available."
